# Hinge‐Like Mechanochromic Mechanophores Based on [2.2]Paracyclophane

**DOI:** 10.1002/anie.202510114

**Published:** 2025-06-30

**Authors:** Shohei Shimizu, Jess M. Clough, Christoph Weder, Yoshimitsu Sagara

**Affiliations:** ^1^ Department of Materials Science and Engineering Institute of Science Tokyo 2‐12‐1 Ookayama, Meguro‐ku Tokyo 152–8550 Japan; ^2^ Adolphe Merkle Institute University of Fribourg Chemin des Verdiers 4 Fribourg CH‐1700 Switzerland; ^3^ NCCR Bio‐inspired Materials University of Fribourg Chemin des Verdiers 4 Fribourg CH‐1700 Switzerland; ^4^ Research Center for Autonomous Systems Materialogy (ASMat) Institute of Integrated Research, Institute of Science Tokyo 4259 Nagatsuta‐cho, Midori‐ku Yokohama Kanagawa 226–8501 Japan

**Keywords:** Cyclophanes, Fluorescence, Mechanochromic luminescence, Polyurethane, Supramolecular mechanophore

## Abstract

A hinge‐like supramolecular mechanophore based on a [2.2]paracyclophane core and two excimer‐forming 1,6‐bis(phenylethynyl)pyrene luminophores is presented. Each luminophore shares one phenyl group with the [2.2]paracyclophane, resulting in a rigid and strained structure that forces the two luminophores into close proximity. As a consequence, the photoluminescence of the mechanophore in THF solution and in solid films of a polyurethane containing the new motif is dominated by excimer emission. Stretching the polymer films causes an easily discernible change from bright yellow excimer to blue–green monomer‐dominated emission. The ratio of excimer to monomer emission intensities traces the nonlinear stress–strain curves of the polymer well and is a good indicator for the macroscopically applied force. The reversibility of the mechanoresponse, theoretical analyses, and reference experiments with a similar mechanophore in which the emitters and the [2.2]paracyclophane core are connected by flexible linkers support the conclusion that the mechanoactivation is caused by distorting the molecule into a bent, more open conformation and not the scission of covalent bonds. The operating principle was further confirmed by investigating a second hinge‐like mechanophore based on a [2.2]paracyclophane core and 1,4‐bis(phenylethynyl)benzene emitters.

## Graphical abstract

Hinge‐like supramolecular mechanophores utilizing [2.2]paracyclophane have been developed. In the force‐free state, the rigid [2.2]paracyclophane forces two luminophores into proximity, leading to excimer‐dominant photoluminescence. Stretching the polyurethane films into which the mechanophores are covalently incorporated results in the structures of the mechanophores changing to more open forms, which leads to monomer‐dominant emission being observed.

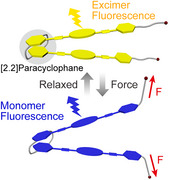

## Introduction

Molecular entities that change their photophysical properties in response to mechanical stimuli in a polymer matrix are useful to evaluate and visualize force‐induced damages that polymer materials may experience. The earliest studies on mechanochromic materials based on this approach involved dye‐doped polymers.^[^ ^1^, ^2^, ^3^, ^4^, ^5^ ^]^ In the as‐prepared materials, the dyes form aggregates that show excimer photoluminescence, but these aggregates are dispersed upon deformation, thereby increasing the intensity of monomer emission. This approach does not require complex preparation procedures, as the dyes are simply doped into the polymer, and mechanochromic materials can be inexpensively produced. However, the strategy is not universally applicable, because the aggregation of the dyes and the mechanoresponsive behavior depend strongly on the nature of the polymer and processing conditions. Furthermore, the mechanochromic response is not directly coupled to the forces experienced by individual polymer chains.

As an alternative, mechanochromic mechanophores that respond to mechanical forces at the single‐molecule level have been widely studied.^[^ ^6^, ^7^, ^8^, ^9^, ^10^, ^11^ ^]^ These motifs are covalently introduced into the backbone or as cross‐links in polymer materials and are activated if the applied force exceeds a given threshold. Most mechanochromic mechanophores including Diels–Alder adducts,^[^ ^12^, ^13^, ^14^, ^15^, ^16^ ^]^ spiro‐cyclic compounds,^[^ ^17^, ^18^, ^19^, ^20^, ^21^ ^]^ 1,2‐dioxetane mechanophores,^[^ ^22^, ^23^, ^24^ ^]^ ladder‐type mechanophores,^[^ ^25^, ^26^ ^]^ persistent radical‐generating mechanophores,^[^ ^27^, ^28^, ^29^ ^]^ and others^[^ ^30^, ^31^, ^32^, ^33^, ^34^ ^]^ are activated by the cleavage of covalent bonds. Moreover, numerous mechanophores that operate without covalent bond scission have been reported.^[^ ^6^ ^]^ In this case, mechanochromic behavior is achieved by force‐induced changes in molecular conformation,^[^ ^35^, ^36^, ^37^, ^38^, ^39^, ^40^, ^41^ ^]^ tuning of fluorescence resonance energy transfer,^[^ ^42^ ^]^ changes in ligand exchange rate,^[^ ^43^, ^44^ ^]^ or alternation in the arrangement of multiple fluorophores or fluorophore‐quencher pairs.^[^ ^45^, ^46^, ^47^, ^48^, ^49^, ^50^, ^51^, ^52^, ^53^, ^54^, ^55^, ^56^, ^57^, ^58^ ^]^ Many of these motifs show mechanochromic responses that are fully reversible because their activation mechanism does not rely on covalent bond cleavage.

Our groups have developed several supramolecular mechanophores based on rotaxane,^[^ ^45^, ^46^, ^47^, ^48^, ^49^ ^]^ cyclophane,^[^ ^50^, ^51^, ^52^, ^53^ ^]^ and loop‐forming^[^ ^54^, ^55^ ^]^ structures, whose photoluminescence characteristics change instantaneously and reversibly in response to mechanical activation. Aside from quencher–emitter pairs that are separated by the applied force and lead to turn‐on fluorescence,^[^ ^45^, ^46^, ^47^, ^48^, ^49^ ^]^ we employed excimer‐forming dyes,^[^ ^50^, ^51^, ^54^, ^55^ ^]^ as well as chromophore pairs forming charge‐transfer complexes,^[^ ^52^, ^53^ ^]^ to create ratiometric force sensors whose emission spectrum changes upon activation. Monitoring the ratio of the emission intensities associated with two characteristic processes, e.g. monomer and excimer emission, makes the quantitative assessment of activation rather straightforward. However, it proved difficult to realize ratiometric, supramolecular mechanophores that display vivid color changes. For example, the perylene diimide emitters used in the loop‐forming mechanophores exhibit a very low quantum efficiency in the excimer state.^[^ ^54^, ^55^, ^56^ ^]^ In the case of a cyclophane‐based mechanophore containing two 1,6‐bis(phenylethynyl)pyrene groups, the π–π interactions between the fluorophores are weak and the formation of the associated (excimer‐forming) state is inefficient in polymers.^[^ ^50^ ^]^

**Figure 1:**
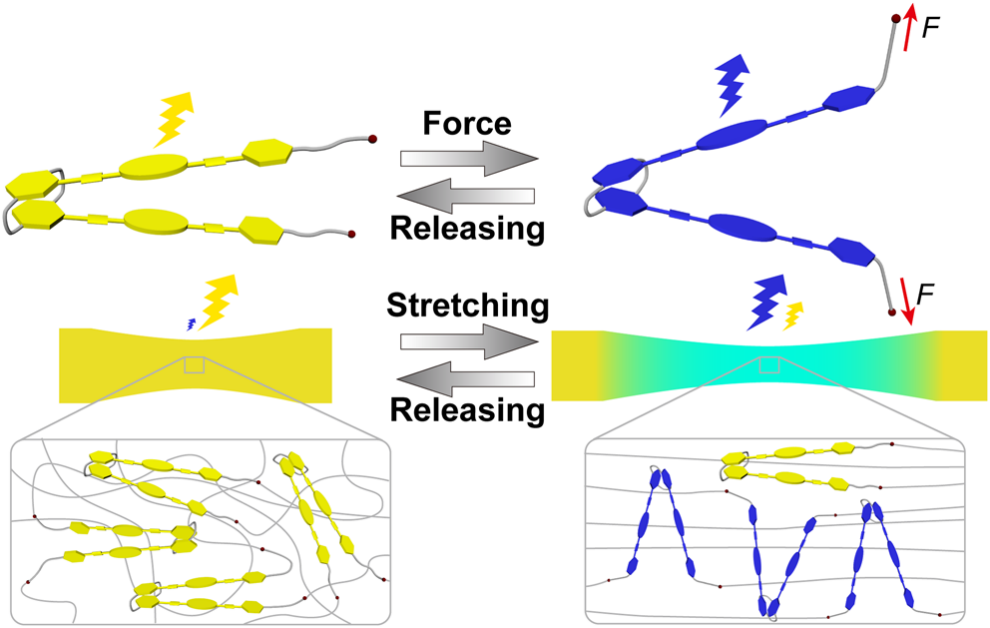
Schematic illustration of mechanochromic luminescence shown by a hinge‐like mechanochromic mechanophore **PC‐Py1**.

Here, we report a novel strategy to achieve highly associated but mechanically switchable intramolecular assemblies between highly emissive, excimer‐forming emitters whose inherent attractive interactions are weak. Our approach involves a hinge‐like supramolecular mechanophore based on a [2.2]paracyclophane core and two rigid, highly emissive, excimer‐forming 1,6‐bis(phenylethynyl)pyrenes (**PC‐Py1**, Figure 1). The [2.2]paracyclophane, in which two benzene rings are linked by two ethylene chains at the para positions, is one of the most well‐known cyclophanes.^[^ ^59^, ^60^ ^]^ Since its first report by Brown and Farthing in 1949,^[^ ^61^ ^]^ extensive research has been conducted regarding π–π interactions,^[^ ^62^, ^63^ ^]^ synthetic chemistry,^[^ ^64^ ^]^ photochemistry,^[^ ^65^, ^66^, ^67^, ^68^ ^]^ polymer chemistry,^[^ ^69^, ^70^, ^71^ ^]^ and other aspects.^[^ ^72^ ^]^ The Abe group reported a *pseudogem*‐bis(diphenylimidazole)[2.2]paracyclophane (*pseudogem*‐bisDPI[2.2]PC) containing one hexaarylbiimidazole (HABI), which exhibits fascinating photochromism.^[^ ^65^ ^]^ The HABI framework is known to generate a pair of 2,4,5‐triphenylimidazolyl radicals upon optical excitation, which can thermally recombine to restore the initial imidazole dimer. Compared to other HABI derivatives, *pseudogem*‐bisDPI[2.2]PC shows fast thermal bleaching, as the [2.2]paracyclophane framework forces the photogenerated imidazolyl radicals into close proximity. Based on these studies, we surmised that substituting the [2.2]paracyclophane core at the *pseudogem* position with excimer‐forming fluorophores might be a viable approach to force strong electronic interactions between the two emitters, even if their intrinsic attractive interactions are weak. We further speculated that the hinged geometry would allow the force‐activated separation of the fluorophores. As we show here, **PC‐Py1**, the first embodiment of this design, indeed displays a change between two highly emissive states and offers all the features of a supramolecular mechanophore.

## Results and Discussion

**Figure 2:**
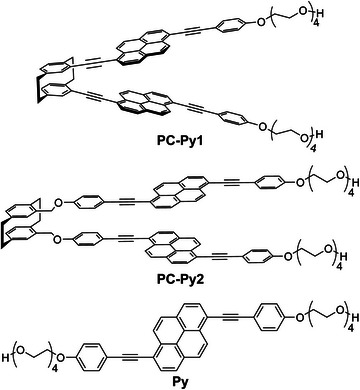
Molecular structures of the hinge‐like mechanophore **PC‐Py1** and reference compounds **PC‐Py2**, and **Py**.

The new hinge‐like mechanophore **PC‐Py1** features two 1,6‐bis(phenylethynyl)pyrene moieties that each share one phenyl group with the [2.2]paracyclophane (Figure 2). The 1,6‐bis(phenylethynyl)pyrene emitter was selected because it is known to display a high photoluminescence quantum efficiency in both monomer and excimer states.^[^ ^50^, ^73^ ^]^ Each fluorophore is equipped with a peripheral tetra(ethylene glycol) handle terminated with a hydroxyl group to enhance the solubility of **PC‐Py1** and allow its covalent incorporation into macromolecules. **PC‐Py1** was prepared via the Sonogashira coupling between *pseudogem*‐diethynyl[2.2]paracyclophane and two equivalents of a pyrene derivative having one tetraethylene glycol terminated with a triisopropylsilyl protection group and subsequent deprotection (Scheme S1).

To elucidate the effect of the rigidity imparted by attaching the emitters to the [2.2]paracyclophane, we also prepared the reference compound **PC‐Py2**, in which two 1,6‐bis(phenylethynyl)pyrenes are connected to the [2.2]paracyclophane via flexible methyl ether spacers. **PC‐Py2** was synthesized through the Williamson ether synthesis between *pseudogem*‐bis(bromomethyl)[2.2]paracyclophane and two equivalents of a 1,6‐bis(phenylethynyl)pyrene derivative featuring one hydroxyl group and one tetraethylene glycol (Scheme S2). Finally, we also synthesized **Py**, another reference compound containing only one 1,6‐bis(phenylethynyl)pyrene. **PC‐Py1**, **PC‐Py2**, and **Py** were characterized to satisfaction by ^1^H, ^13^C NMR, and electrospray ionization mass spectroscopy (see Supporting Information).

The ^1^H NMR spectra reveal that the pyrene peaks in **PC‐Py1** experience upfield shifts compared to those of **Py**, which we attribute to intramolecular interactions between the fluorophores (Figure S1). Although **PC‐Py1** can, in principle, adopt four different conformations (two parallel and two antiparallel ones, Figure S2), the peaks in the ^1^H NMR spectrum of **PC‐Py1** are neither split nor broadened. All protons in the aromatic region of **PC‐Py1** were fully assigned on the basis of ^1^H‐^1^H COSY, ^1^H‐^1^H ROESY, and ^1^H‐^13^C HMBC spectra of **PC‐Py1‐TIPS** (see Supporting Information). Correlations in the ROESY NMR spectrum indicate that **PC‐Py1** adopts a slightly open conformation that allows fast, partial rotational motion of the pyrene moieties. The smaller upfield shifts of the pyrene peaks in the ^1^H NMR spectrum of **PC‐Py2** compared to those of **PC‐Py1** further support the conclusion that the pyrene moieties in **PC‐Py1** remain, as designed, in close proximity, due to the rigidity of the [2.2]paracyclophane framework, regardless of their rotational freedom.

**Figure 3:**
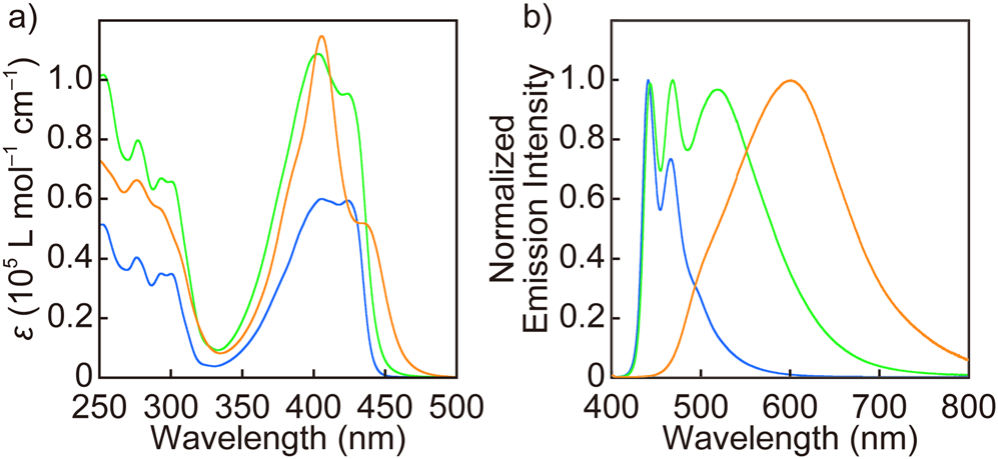
a) UV–vis absorption and b) photoluminescence spectra of **PC‐Py1** (orange), **PC‐Py2** (green), and **Py** (blue) in THF (*c* = 1.0 × 10^−5^ M). *λ*_ex_ = 400 nm.

UV–vis absorption and photoluminescence spectra of **PC‐Py1**, **PC‐Py2**, and **Py** were recorded in dilute tetrahydrofuran (THF) solutions (*c* = 1.0 × 10^−5^ M) (Figure 3). The photophysical characteristics of **Py** are comparable to the previously reported properties of the 1,6‐bis(phenylethynyl)pyrene monomer.^[^ ^73^ ^]^ The UV–vis absorption spectrum displays a split band with similarly strong maxima at 406 and 424 nm, while the photoluminescence spectrum shows narrow peaks at 441 and 466 nm. **Py** displays a quantum yield of 90% and an emission lifetime of 1.2 ns (Figure S5a and Table S1).

Since **PC‐Py1** and **PC‐Py2** contain two chromophores, their molar extinction coefficients are higher than that of **Py**, and their absorption bands adopt a rather different shape, due to intramolecular ground state interactions between the fluorophores (Figure 3a). The absorption bands feature again two maxima at 406/434 nm (**PC‐Py1**) and 404/423 nm (**PC‐Py2**), but in each case, the longer‐wavelength peak displays a lower extinction coefficient than its shorter‐wavelength counterpart. This effect is more pronounced for **PC‐Py1**, which suggests that the intramolecular ground state interactions are more pronounced than in **PC‐Py2**. Both spectra are broadened in comparison to that of **Py**, and especially in the case of **PC‐Py1**, a low‐energy tail is seen, which indicates through‐space conjugation facilitated by the [2.2]paracyclophane^[^ ^68^ ^]^ and exciton coupling.

The photoluminescence spectrum of **PC‐Py1** is completely void of peaks associated with the 1,6‐bis(phenylethynyl) pyrene monomer but instead displays a broad excimer peak at 601 nm. **PC‐Py1** displays a quantum yield of 27%, which is appreciable for an excimer. The emission spectrum of **PC‐Py2** shows a vibronic structure with peaks at 442 and 468 nm that are diagnostic for monomer emission, and an additional broad peak at 518 nm that we attribute to excimer emission (Figure 3b). The photoluminescence quantum efficiency, measured over both bands, is 68%. The photophysical characteristics of **PC‐Py2** reflect that the two methyl ether linkers allow the two fluorophores to adopt more varied conformations than are possible in **PC‐Py1**, resulting in a mix of strong monomer and excimer emission as well as a less pronounced red‐shift of the excimer band. The photoluminescence spectra of THF solutions of **PC‐Py1** and **PC‐Py2** are concentration‐independent (Figure S6), which confirms that in both cases excimer formation is an intramolecular, not intermolecular, process. Excimer formation in **PC‐Py1** and **PC‐Py2** is confirmed by emission lifetime measurements (THF, *c* = 1.0 × 10^−6^ M, Figure S5 and Table S1). In the case of **PC‐Py1**, two different excimer emission lifetimes of 17 and 24 ns are observed, presumably because **PC‐Py1** can only form two different excimers in which the luminophores adopt parallel or antiparallel arrangements (Figure S2). By contrast, emission lifetime measurements for **PC‐Py2** reveal three distinct decay processes with lifetimes of 0.93, 5.8, and 23 ns, which we relate to monomer, partial excimers, and excimers, respectively.^[^ ^74^ ^]^

To explore the mechanochromic behavior of **PC‐Py1** and **PC‐Py2**, we prepared segmented polyurethane elastomers,^[^ ^75^ ^]^ which combine high strength and high extensibility with reversible deformation behavior and are particularly suitable for evaluating the response of supramolecular and other reversible mechanophores.^[^ ^20^, ^28^, ^37^, ^38^, ^39^, ^43^, ^44^, ^45^, ^46^, ^47^, ^48^, ^49^, ^50^, ^52^, ^53^, ^55^, ^58^ ^]^ Thus, the mechanophores **PC‐Py1** and **PC‐Py2**, and the reference monomer **Py** were individually incorporated into the backbone of polyurethanes made by the polyaddition of a poly(tetrahydrofuran)diol (PTHF), 4,4′‐methylenebis(phenyl isocyanate), and 1,4‐butanediol (see Supporting Information). The resulting polymers, referred to as **PC‐Py1‐PU**, **PC‐Py2‐PU**, and **Py‐PU**, display number‐average molecular weights, *M*_n_, of 125000, 123000, and 142000, respectively, and contain the respective mechanophore/reference fluorophore in a concentration of 0.005, 0.006, and 0.007 wt%, respectively. Due to the low concentration, the fluorophores are not detectable in the ^1^H NMR spectra of the polyurethanes (Figure S8), but their introduction is confirmed by absorption and photoluminescence spectra (THF, *c* = 5.0 mg mL^−1^) (Figure S9). In solution, the three polymers display photophysical properties that are virtually identical to those of the parent mechanophores/reference compound.

Films of **PC‐Py1‐PU**, **PC‐Py2‐PU**, and **Py‐PU** with a thickness of 80–100 µm were prepared by solvent‐casting the polymers from THF solutions and subsequent drying (see Supporting Information). Due to the similar molecular weight and the low concentration of the mechanophores/reference fluorophore, the thermal and mechanical properties of the three polymers are virtually identical. Differential scanning calorimetry scans show endothermal peaks around 10 and 180 °C, which are associated with the melting of crystalline PTHF domains and the dissociation of urethane hard segments, respectively (Figure S10), while thermogravimetric analysis traces show a 5% weight loss at ca. 305 °C (Figure S11). Tensile tests reveal a stress at break of ca. 40 MPa, an elongation at break of ca. 700%, and a Young's modulus of ca. 5.5 MPa (Figure S12 and Table S2).

**Figure 4:**
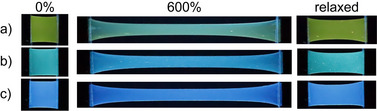
Images of a) **PC‐Py1‐PU**, b) **PC‐Py2‐PU**, and c) **Py‐PU** films in the unstretched state (left), stretched to 600% (middle), and after removing the applied stress (right). The images were taken in the dark with excitation light at 365 nm.

Figures 4a, S13a, and Supporting Video S1 show strikingly that the **PC‐Py1‐PU** films display a clearly discernible mechanochromic response; the photoluminescence color changes from bright yellow to turquoise upon deformation. The response is immediately reversed when the applied force is removed and the film is allowed to relax. A much more subtle change in the emission color is observed for **PC‐Py2‐PU** films, for which different hues of blue are observed, while **Py‐PU** only shows blue fluorescence, irrespective of the strain (Figures 4 and S13, and supporting Videos S2 and S3).

**Figure 5:**
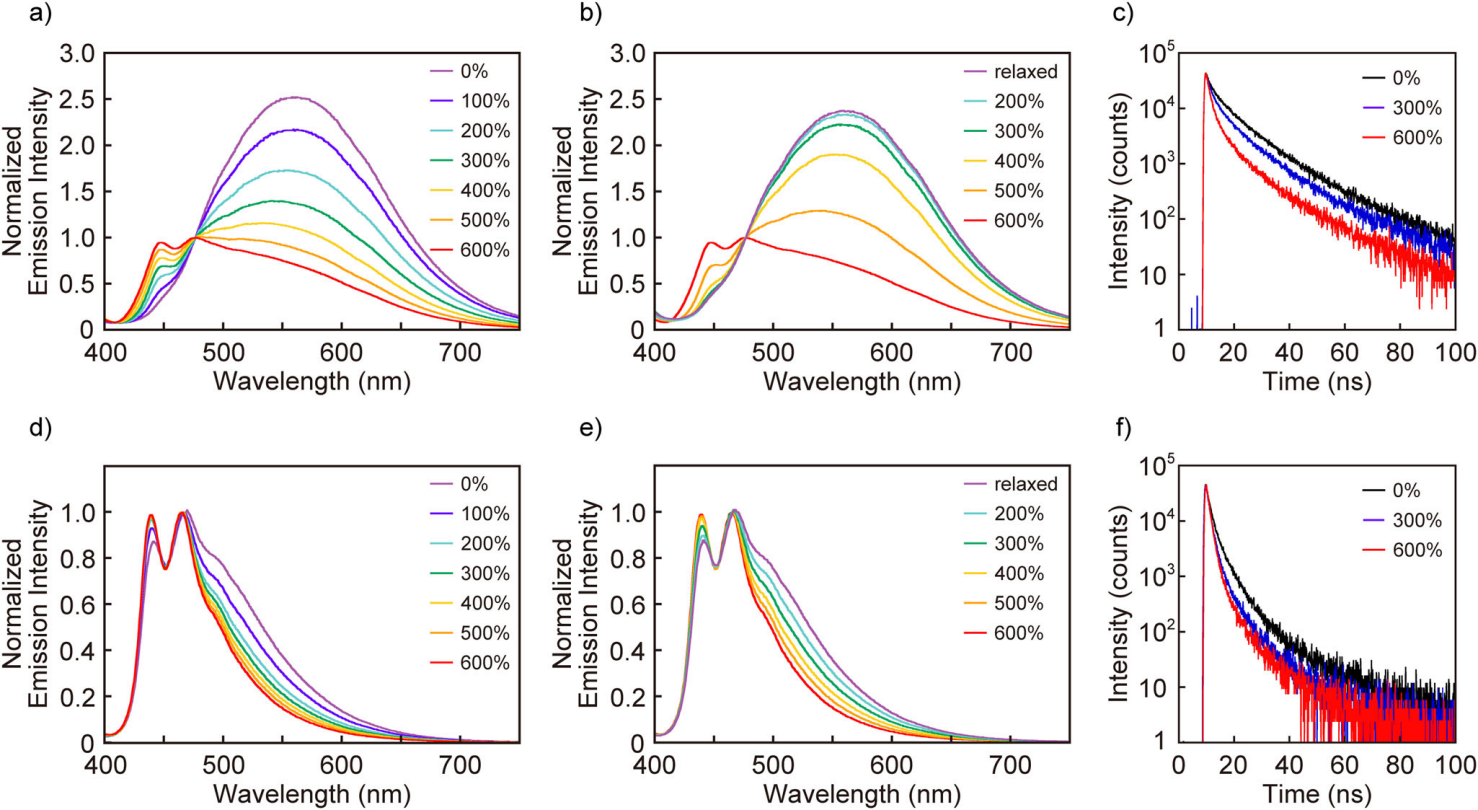
a) and b) Photoluminescence spectra of a **PC‐Py1‐PU** film upon a) stretching and b) relaxing the film to the indicated strains. The spectra were normalized to the intensity at 476 nm. c) Emission decay profiles of the **PC‐Py1‐PU** film upon stretching. d) and e) Photoluminescence spectra of a **PC‐Py2‐PU** film upon d) stretching and e) relaxing the film to the indicated strains. The spectra were normalized to the intensity at 465 nm. f) Emission decay profiles of the **PC‐Py2‐PU** film upon stretching. Excitation light of 365 nm was used for measuring the photoluminescence spectra. Emission decays were recorded with excitation light of 405 nm and monitored at 500 nm.

Tensile tests coupled with in situ photoluminescence spectroscopy were employed to investigate the mechanochromic response of **PC‐Py1‐PU**, **PC‐Py2‐PU**, and **Py‐PU** films in more detail (Figures 5 and S14a). As expected, the **Py‐PU** reference polymer displays exclusively monomer emission, and the changes observed in the emission spectra and the emission decay profiles upon deformation are negligible (Figure S14). By contrast, the emission spectrum of **PC‐Py1‐PU** in the stress‐free state exhibits only a broad, structureless excimer emission band with a maximum of 560 nm (Figure 5a). The maximum of this band is blue‐shifted compared to the spectrum recorded in THF (Figure S15a), likely on account of the reduced mobility of the mechanophores in the solid polymers, which allows the fluorophores to emit before full structural relaxation is possible.^[^ ^74^ ^]^ Upon stretching the **PC‐Py1‐PU** film, the excimer emission intensity gradually decreases, while the characteristic monomer emission bands with maxima at 447 and 476 nm develop (Figure 5a). Their intensity increases with the applied strain and eventually exceeds that of the excimer band. The effect is reversible, i.e., the monomer bands decrease as the stress is released (Figure 5b). Thus, as intended, the macroscopically applied force causes the spatial separation of the two luminophores so that intramolecular excimer formation is suppressed. The fact that the effect is reversible supports that it is caused by distorting the molecule into a bent, more open conformation (Figure 1), and not the covalent bond scission. This interpretation is supported by photoluminescence lifetime measurements (Figures 5c, S16a, and S17). The emission decay of the **PC‐Py1‐PU** films in the force‐free state can be fitted with a tri‐exponential decay function, and the longest emission lifetime observed (23 ns) corresponds to excimer fluorescence. Upon deformation, the relative contribution of the most rapid decay process, associated with monomer emission, increases, and the overall emission decay is accelerated (Figures 5c and S17). Conversely, the original decay kinetics are restored when the force is released (Figures S16a and S17).

The emission spectra of **PC‐Py2‐PU** films in the force‐free state are dominated by monomer emission with peaks at 441 and 469 nm (Figure 5d). Intramolecular excimer formation is significantly suppressed compared to the THF solutions of **PC‐Py2‐PU** and **PC‐Py2** (Figures S15b and 3b) and appears only as a weak shoulder at around 520 nm. This indicates that during film formation, a large fraction of the fluorophores is kinetically trapped in conformations that do not promote excimer formation. Nevertheless, the relative intensity of the excimer emission band gradually decreases when the films are deformed (Figure 5d), and reappears once the stress is released (Figure 5e). Also in this case, stretching the films leads to a (reversible) change in the emission lifetime (Figures 5f and S16b). However, this effect, which is consistent with the minor changes observed in the emission spectra, is less pronounced than for **PC‐Py1‐PU**. The mechanochromic response of **PC‐Py2‐PU** is similar to that of a previously reported polyurethane containing a cyclophane‐based mechanophore in which two similar luminophores are bridged by flexible triethylene glycol chains.^[^ ^50^ ^]^ The comparison of these materials strikingly reflects the impact of the rigid architecture of **PC‐Py1**. While the extent of electronic interactions between the emitters is limited in the more flexible mechanophore **PC‐Py2**, the conformational constraints at play in **PC‐Py1** ensure the close proximity of the two luminophores, as long as no external mechanical force is applied.

**Figure 6:**
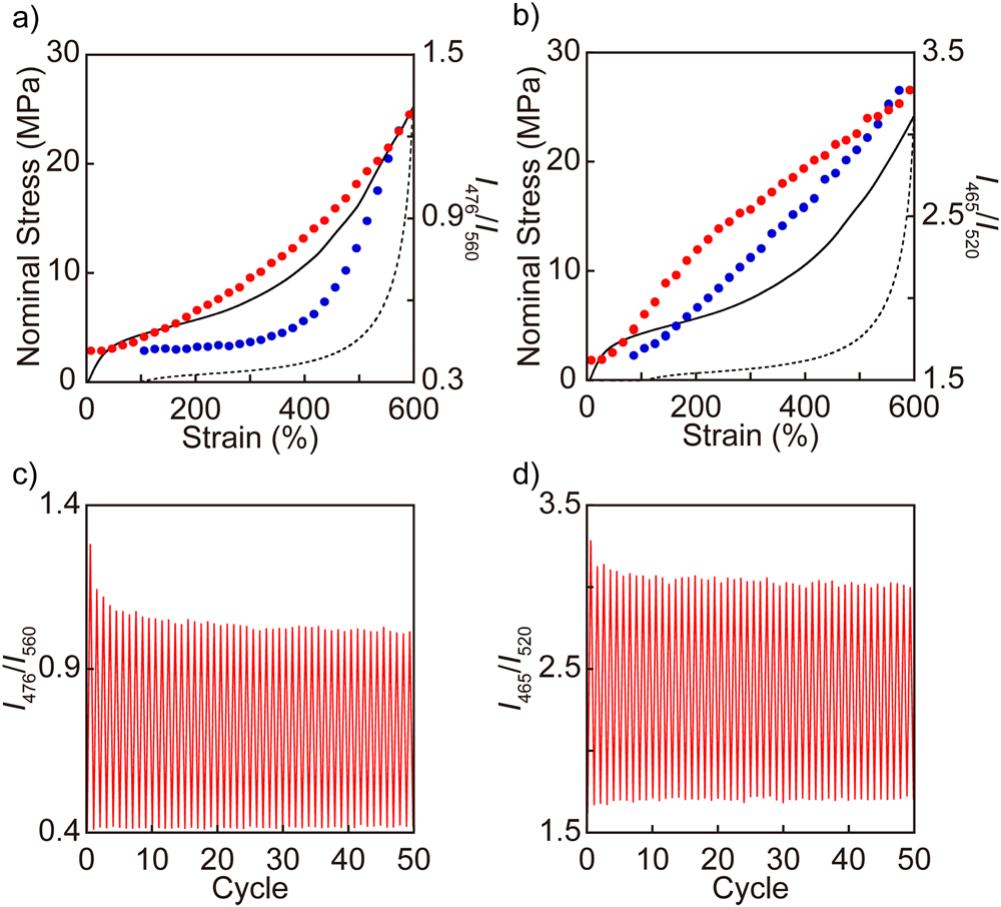
a) and b) Overlays of stress–strain curves (stretching: solid lines, relaxing: dotted lines) and relative monomer to excimer emission intensity *I*_476_/*I*_560_ for a) a **PC‐Py1‐PU** film and *I*_465_/*I*_520_ for b) a **PC‐Py2‐PU** film (stretching: red circles, relaxing: blue circles). c) and d) Plots of the *I*_476_/*I*_560_ and *I*_465_/*I*_520_ ratios upon stretching and relaxing c) a **PC‐Py1‐PU** and d) a **PC‐Py2‐PU** films over 50 cycles, respectively. Data were recorded after each step in the relaxed state and at a strain of 600%.

Overlays of the stress–strain curves and the ratio of monomer‐to‐excimer emission intensities recorded at 476/560 nm for **PC‐Py1‐PU** and 465/520 nm for **PC‐Py2‐PU**, respectively (*I*_476_/*I*_560_ and *I*_465_/*I*_520_, “ratiometric” signals that are largely independent of acquisition conditions and reliably expresses the fluorescence color of the materials) reveal another difference in the mechanochromic behavior between **PC‐Py1‐PU** and **PC‐Py2‐PU** (Figures 6a,b, S18, and S19). In the case of **PC‐Py1‐PU** films, the *I*_476_/*I*_560_ trace mirrors the nonlinear stress–strain curve acquired upon applying an increasing level of stress rather well. Upon force removal, the stress–strain curve shows a large hysteresis, which is typical for this type of polymer.^[^ ^75^ ^]^ This effect is clearly captured by the *I*_476_/*I*_560_ trace and reflects that upon force removal, the induced strain decrease provides a considerable driving force for the mechanophore to return to its stress‐free conformation. Figures 6a and S18a show that the *I*_476_/*I*_560_ value does not linearly depend on the applied strain, while plots of the applied stress versus *I*_476_/*I*_560_ show more linear dependencies (Figure S19a). The corresponding experiment with **PC‐Py2‐PU** films paints a rather different picture (Figures 6b, S18b, and S19b). In this case, the *I*_465_/*I*_520_ value scales in a linear manner with the applied strain, and little hysteresis is observed in the *I*_465_/*I*_520_ signal when the stress is removed. This behavior is attributed to the reduced rigidity of **PC‐Py2**, which in turn reduces the driving force to return to the initial conformation in comparison to **PC‐Py1**.

In order to investigate the reversibility of the mechanochromic response, **PC‐Py1‐PU** and **PC‐Py2‐PU** films were subjected to 50 stretch and release cycles, and the *I*_476_/*I*_560_ for **PC‐Py1‐PU** film and the *I*_465_/*I*_520_ for **PC‐Py2‐PU** film values were recorded after each step in the relaxed state and at 600% strain (Figure 6c,d). In both cases, the mechanochromic response demonstrates excellent reversibility over 50 cycles, confirming that no significant covalent bond breakage or force‐induced rotational conversion to a [2.2]paracyclophane derivative substituted at the *pseudometa* position occurs. Moreover, we confirmed that in the 51st stretch and release cycle, the *I*_476_/*I*_560_ and *I*_465_/*I*_520_ ratios of **PC‐Py1‐PU** and **PC‐Py2‐PU** films continue to show correlations with stress and strain, respectively (Figure S20).

To confirm that working mechanism of **PC‐Py1** is indeed derived from the rigidity of the [2.2]paracyclophane moiety and not simply related to a change of the dihedral angle between the pyrene rings and the phenyl groups of the [2.2]paracyclophane, the new hinge‐like mechanophore **PC‐Be**, in which the pyrene groups are replaced with benzene rings, along with a corresponding reference monomer **Be** (Scheme S3) was synthesized. **PC‐Be** only shows a hinge‐like motion upon mechanical stress for activation. The fluorescence spectrum of **PC‐Be** in THF solution shows a broad excimer emission that is red‐shifted relative to the emission band of the monomer **Be** (Figure S21b), and the fluorescence lifetime is also distinct from that of the monomer species (Figure S22). Subsequently, **PC‐Be** was covalently incorporated into a polyurethane, and **PC‐Be‐PU** films were prepared in the same manner as **PC‐Py1‐PU** films (see Supporting Information). Upon uniaxial stretching of the **PC‐Be‐PU** films, the monomer emission intensity in the shorter wavelength region increased relative to the excimer emission, and the fluorescence spectrum recovered to its original state upon releasing the force (Figure S23). Furthermore, as shown in Figure S25, the ratio of monomer to excimer fluorescence intensity changes in correlation with the applied stress. Given that the fluorescence response of the **PC‐Py1‐PU** films is similar to that of the **PC‐Be‐PU** films, we conclude that the working mechanism of **PC‐Py1** involves a hinge‐like motion.

To demonstrate that covalent bond incorporation of the mechanophores is vital for their activation, physically doped **PC‐Py1inPU** and **PC‐Py2inPU** films were prepared by solvent‐casting mixtures of a chromophore‐free reference polyurethane **PU** and small amounts of **PC‐Py1** or **PC‐Py2** (see Supporting Information). As before, photoluminescence spectra were recorded for each film upon deformation and subsequent relaxation (Figures S26 and S27, Supporting Videos S4 and S5). In the case of the **PC‐Py1inPU** film, no spectral changes are observed, indicating that direct force transduction through covalent bonds is required to activate **PC‐Py1**. By contrast, the emission spectra acquired for the **PC‐Py2inPU** film show a gradual decrease of the shoulder at 520 nm associated with excimer emission, although a plot of the *I*_465_/*I*_520_ ratio against the applied strain reveals a much smaller change than observed for **PC‐Py2‐PU**. In light of the widely accepted notion that a mechanophore must be covalently coupled to the polymer matrix in order to be activated,^[^ ^6^, ^7^, ^8^, ^9^, ^10^, ^11^ ^]^ this behavior is at first surprising. Nevertheless, numerous examples of mechanochromic blends based on various polymers and excimer‐forming additives have been documented,^[^ ^1^, ^2^, ^3^, ^4^, ^5^, ^76^, ^77^, ^78^ ^]^ whose mechanoresponse does not require such covalent connections. In the case of **PC‐Py2inPU**, the mechanoresponse is related to conformational changes, which appear to be enabled by entanglements of the polymer chains and the long and rigid emitters, and the small forces that required to change the conformation of **PC‐Py2**.

To gain further insight into the difference between the mechano‐responsive behavior of **PC‐Py1** and **PC‐Py2**, constrained geometries simulate external force (CoGEF) calculations (DFT CAM‐B3LYP/6–31+G(d,p)) were performed on simplified molecular structures **PC‐Ref1** and **PC‐Ref2** (Figure S28). Relative potential energies of the optimized geometry were plotted with an extended distance between carbon atoms [a_1_,b_1_] and [a_1_’,b_1_’], respectively. The relative potential energy calculated for **PC‐Ref1** gradually increases upon the separation between the carbon atoms. Simultaneously, the [2.2]paracyclophane structure is gradually distorted because the carbon distance between [c_1_,d_1_] on [2.2]paracyclophane in **PC‐Ref1** is simultaneously extended. In contrast, when the distance between [a_1_’,b_1_’] in **PC‐Ref2** increases, the ether chain rearranges the conformation and the carbon distance between [c_1_’,d_1_’] is almost constant within 3.17–3.25 Å, suggesting the suppression of the distortion in [2.2]paracyclophane structure of **PC‐Ref2**. Once the distortion of [2.2]paracyclophane starts, the potential energy significantly starts increasing. Based on these results, we speculate that change in relative monomer to excimer of **PC‐Py1** would occur during the gradual distortion of the [2.2]paracyclophane, while the activation of **PC‐Py2** can occur before distortion of the [2.2]paracyclophane. Therefore, the two supramolecular mechanophores were expected to show different mechanochromic behavior.

## Conclusion

In summary, hinge‐like supramolecular mechanophores utilizing a rigid [2.2]paracyclophane scaffold were developed. The rigid connection with the [2.2]paracyclophane forces the attached luminophores into a conformation in which they exhibit exclusively excimer photoluminescence, even if their intrinsic attractive interactions are weak. The comparison of different motifs reveals that the activation mechanism is governed by the high molecular rigidity and involves a hinge‐like motion. This behavior contrasts with that of other mechanophores based on similar luminophores, in which excimer formation does not rely on the geometric prearrangement but is largely driven by attractive interactions among the emitters, which generally leads to less pronounced excimer formation.^[^ ^50^, ^51^ ^]^ Gratifyingly, mechanical activation distorts the molecule into a bent, more open conformation, in which monomer emission is dominant. As a consequence, films of polyurethanes that contain the new mechanophore display a clearly discernible mechanochromic response that is instantly reversible and scales in a linear manner with the applied stress rather than strain. We speculate that the strategy of using [2.2]paracyclophane to design hinge‐like mechanophores can be exploited with any pair of electronically interacting fluorophores and fluorophore/quencher pairs, as the proximity of these motifs in the idle state is geometrically controlled and independent of the intrinsic association constant.

## Supporting Information

Supporting Information includes Videos S1–S5, Figures S1–S28, and Tables S1 and S2, as well as a detailed description of the materials, experimental methods, synthetic procedures, and analytical data for all compounds. The authors have cited additional references within the Supporting Information.^[^ ^79^, ^80^, ^81^ ^]^

## Conflict of Interests

The authors declare no conflict of interest.

## Supporting information



Supporting Information

Supporting Information

Supporting Information

Supporting Information

Supporting Information

Supporting Information

## Data Availability

The data that support the findings of this study are available from the corresponding author upon reasonable request.
