# Supplementary material for: Hinge‐Like Mechanochromic Mechanophores Based on [2.2]Paracyclophane
Source: Angew Chem Int Ed Engl. 2025 Jun 30;64(37):e202510114. doi: 10.1002/anie.202510114 (PMC12416447; doi:10.1002/anie.202510114)
Supplement: Supplementary file 1 — Supporting Information [file ANIE-64-e202510114-s003.pdf]

## Supporting Information

# Hinge-like Mechanochromic Mechanophores Based on [2.2]Paracyclophane

Shohei Shimizu, Jess M. Clough, Christoph Weder, and Yoshimitsu Sagara*

E-mail: sagara@mct.isct.ac.jp

## Table of Contents

| | |
|---|---|
| **General methods** | **S2** |
| **Synthesis of PC-Py1, PC-Py2, Py, PC-Be, and Be** | **S3** |
| **NMR analyses** | **S10** |
| **Emission decay curves of PC-Py1, PC-Py2, and Py in THF** | **S12** |
| **Photoluminescence spectra of PC-Py1 and PC-Py2 as a function of concentration** | **S12** |
| **Photophysical properties in different solvents** | **S13** |
| **Polymer synthesis** | **S14** |
| **Preparation of polyurethane films** | **S16** |
| **$^1$H NMR spectra of the polyurethanes** | **S17** |
| **Absorption and photoluminescence spectra of the polyurethane solutions** | **S18** |
| **Thermal properties of the polyurethanes** | **S18** |
| **Mechanical properties of the polyurethane films** | **S20** |
| **Images of the PC-Py1-PU, PC-Py2-PU, and Py-PU films upon uniaxial deformation** | **S21** |
| **Absence of mechanochromic behavior of the Py-PU film upon stretching** | **S21** |
| **Emission spectra of the polyurethane films and solutions** | **S21** |
| **Emission decay profiles of the PC-Py1-PU and PC-Py2-PU films** | **S22** |
| **Overlays of true stress-strain curves and emission ratios** | **S22** |
| **Plots of the emission ratio against the true stress** | **S23** |
| **Mechanochromic response in the 51st stretch and release cycle** | **S23** |
| **Photophysical properties of PC-Be and Be** | **S24** |
| **Mechanochromic behavior of PC-Be-PU and Be-PU films** | **S24** |
| **Mechanochromic behavior of PC-Py1inPU and PC-Py2inPU films** | **S26** |
| **DFT calculations** | **S27** |
| **NMR spectra** | **S28** |
| **Supporting references** | **S47** |

## General methods

All reagents and solvents were purchased from Merck, Tokyo Kasei, or FUJIFILM WAKO Pure Chemical Corporation. All reactions were conducted under nitrogen atmosphere. Flash silica gel column chromatography was carried out with a Biotage Isorela flash system using SHOKO-scientific Purif-Pack-EX, Yamazen UNIVERSAL COLUMN, or Yamazen Hi-Flash COLUMN cartridges. Recycling preparative gel permeation chromatography (GPC) was performed with a Japan Analytical Industry LaboACE. $^{1}$H, $^{13}$C, $^{1}$H-$^{1}$H COSY, and $^{1}$H-$^{13}$C HMBC NMR spectra were measured with a JEOL JNM-ECZ400S/L1, and a $^{1}$H-$^{1}$H ROESY NMR spectrum was measured with a Bruker Avance DPX 400 spectrometer at room temperature (r.t.) unless specified otherwise. All chemical shifts of $^{1}$H NMR spectra are reported on the $\delta$-scale in ppm relative to the signal of tetramethylsilane (TMS, at 0.00 ppm) or residual solvent protons (THF at 1.72 ppm) as an internal standard. Coupling constants (*J*) are quoted in Hz and relative intensities are reported. All chemical shifts of $^{13}$C NMR spectra are expressed in ppm using solvents as the internal standards ($CDCl_3$ at 77.16 or DMSO-$d_6$ at 39.52 ppm). Matrix Assisted Laser Desorption Ionization Time-of-Flight (MALDI-TOF) mass spectroscopy was performed with a SHIMADZU MALDI-8030. High-resolution electrospray ionization (ESI) mass spectroscopy was performed with a Bruker Daltonics micrOTOF II. For polymer preparation, inhibitor-free anhydrous THF (FUJIFILM Wako Pure Chemical Corporation) was used as the solvent. Hydroxy-terminated poly(tetrahydrofuran) (PTHF) ($M_n$ = 2,000) was dried in vacuo at 100 ºC over molecular sieves for 12 h before polymerization. 4,4'-Methylenebis(phenyl isocyanate) (MDI) and 1,4-butanediol (BDO) were distilled under reduced pressure and stored over molecular sieves in the fridge and at r.t., respectively. Molecular weights of polymers were measured by a SHIMADZU Nexera GPC system equipped with a GPC KF-805L column (ID = 8.0 mm, L = 300 mm, particle size = 10 µm). Samples were injected using THF as the eluent at 40 °C and a flow rate was 1.0 mL $min^{-1}$. Data was evaluated on LabSolutions software (SHIMADZU) and molecular weights were calculated based on standard polystyrene calibration (1,100–2,500,000). Differential scanning calorimetry (DSC) measurements were performed with a Hitachi DSC7020 under $N_2$ at heating and cooling rates of 10 °C/min. Thermogravimetric analyses (TGA) were also performed under $N_2$ with a SHIMADZU DTG-60. The heating rate was 10 °C/min. Stress-strain measurements were conducted under ambient conditions with a SHIMADZU AGS-100NX equipped with a 100 N load cell at a strain rate of 0.2 $s^{-1}$. The true stress was calculated under the assumption of constant volume. UV-vis absorption spectra were measured on a JASCO V-750. Steady-state fluorescence spectra of solutions were recorded with a JASCO FP-6500 and the spectra were corrected for the detector nonlinearity. Steady-state fluorescence spectra of polyurethane films were monitored with an Ocean Insight QEPro-FL equipped with an LLS-365 or LMS-310 LED light source and a Reflection/Backscattering Probe R400-7-UV-VIS. These spectra were not corrected. Time-resolved fluorescence microscopy was performed with a Hamamatsu Photonics Quantaurus-Tau. Measurements of fluorescence quantum yields were carried out with a Hamamatsu Photonics Quantaurus-QY. Photographs and movies were taken with a Canon EOS 9000D stabilized with a tripod. Density functional theory (DFT) calculations were carried out on the TSUBAME 4.0 supercomputer at Institute of Science Tokyo.

## Synthesis of PC-Py1, PC-Py2, Py, PC-Be, and Be

The procedures for the syntheses of **PC-Py1**, **PC-Py2**, **Py**, **PC-Be**, and **Be** are shown in Schemes S1, S2, and S3. Compounds **1**, **3**, *pseudogem*-diethynyl[2.2]paracyclophane, **6**, and *pseudogem*-bis(bromomethyl)[2.2]paracyclophane were prepared according to reported procedures.[S1–S5]

### Scheme S1

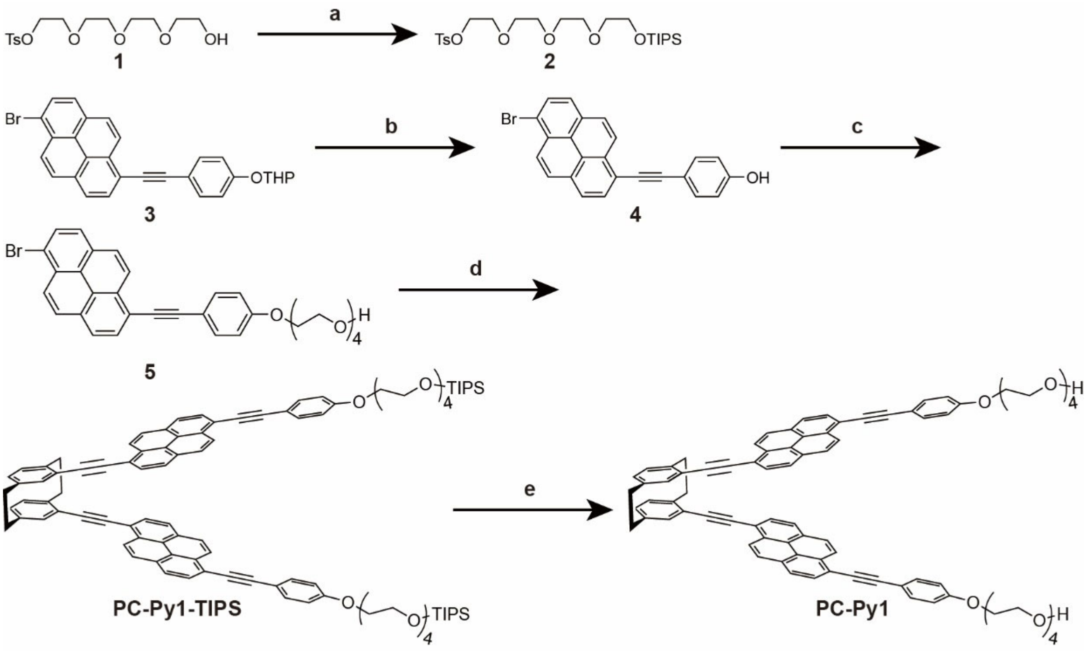


Conditions: (a) triisopropylsilyl chloride, imidazole, $CH_2Cl_2$, r.t., 2 h; (b) 5% aq. HCl, THF, reflux, 2 h; (c) compound **2**, $K_2CO_3$, DMF, 40 h, 80 ºC; (d) *pseudogem*-diethynyl[2.2]paracyclophane, $Pd(PPh_3)_4$, CuI, *i*-$Pr_2NH$, THF, 14 h, 80 ºC; (e) tetrabutylammonium fluoride, THF, 2 h, r.t.

**Compound 2**. Triisopropylsilyl chloride (3.77 g, 19.5 mmol) was added to a $CH_2Cl_2$ solution (50 mL) of compound **1** (5.67 g, 16.3 mmol) and imidazole (3.32 g, 48.8 mmol). The mixture was then stirred at r.t. for 2 h. The reaction mixture was poured into $CH_2Cl_2$ (100 mL), and the organic layer was washed with water (50 mL × 2) and brine (50 mL). The organic layer was dried over $MgSO_4$ and filtered before the solvent was evaporated under reduced pressure. The crude product thus isolated was purified by flash column chromatography on silica gel (eluent: from hexane/ethyl acetate = 19:1 v/v to hexane/ethyl acetate = 4:1 v/v) to yield compound **2** (6.70 g, 13.3 mmol, 81%) as a colorless liquid.

$^1$H NMR (400 MHz, $CDCl_3$): $\delta$ = 1.01–1.16 (m, 21H), 2.45 (s, 3H), 3.56–3.59 (m, 6H), 3.60–3.62 (m, 2H), 3.65–3.66 (m, 2H), 3.68 (t, $J$ = 4.8 Hz, 2H), 3.83 (t, $J$ = 5.6 Hz, 2H), 4.16 (t, $J$ = 4.8 Hz, 2H), 7.34 (d, $J$ = 8.0 Hz, 2H), 7.80 (d, $J$ = 8.4 Hz, 2H). $^{13}$C NMR (100 MHz, $CDCl_3$): $\delta$ = 11.97, 18.03, 21.72, 62.99, 68.71, 69.32, 70.58, 70.75, 70.79, 70.83, 72.78, 128.05, 129.89, 132.98, 144.87. MS (MALDI-TOF): m/z: 504.50 (calcd. $[M]^+$ = 504.26).

**Compound 4**. 5% aq. HCl (10 mL) was added to a THF solution (30 mL) of compound **3** (327 mg, 0.679 mmol). After the reaction mixture was stirred under reflux for 2 h, the mixture was poured into ethyl acetate (100 mL), and the organic layer was washed with water (50 mL), saturated aq. $NaHCO_3$ (50 mL), and brine (50 mL). The organic

layer was dried over $MgSO_4$ and filtered before the solvent was evaporated under reduced pressure. The crude product thus isolated was purified by reprecipitation from a mixture of hexane and ethyl acetate to yield compound **4** (213 mg, 0.536 mmol, 79%) as a yellow solid.

$^1$H NMR (400 MHz, $CDCl_3$): $\delta$ = 4.96 (s, 1H), 6.91 (d, $J$ = 8.8 Hz, 2H), 7.63 (d, $J$ = 8.4 Hz, 2H), 8.06 (d, $J$ = 8.0 Hz, 1H), 8.12–8.19 (m, 3H), 8.22 (d, $J$ = 8.0 Hz, 1H), 8.26 (d, $J$ = 8.4 Hz, 1H), 8.46 (d, $J$ = 9.2 Hz, 1H), 8.68 (d, $J$ = 9.2 Hz,1H). $^{13}$C NMR (100 MHz, DMSO-$d_6$): $\delta$ = 86.22, 96.77, 112.45, 115.98, 118.67, 119.86, 123.15, 124.79, 125.49, 125.74, 125.88, 126.82, 128.47, 129.04, 129.42, 130.07, 130.27, 130.30, 130.77, 130.81, 133.44, 158.54. MS (MALDI-TOF): m/z: 396.66 (calcd. $[M]^+$ = 396.01).

**Compound 5**. Compound **4** (182 mg, 0.458 mmol) was added to a mixture of compound **2** (462 mg, 0.916 mmol) and $K_2CO_3$ (316 mg, 2.29 mmol) in DMF (20 mL). After the mixture was stirred for 40 h at 80 ºC, the reaction mixture was poured into ethyl acetate (50 mL), and the organic layer was washed with saturated aq. $NH_4Cl$ (4 × 50 mL) and brine (50 mL). The organic layer was dried over $MgSO_4$ and filtered before the solvent was evaporated under reduced pressure. The crude product thus isolated was purified by flash column chromatography on silica gel (eluent: from hexane/ethyl acetate = 9:1 v/v to hexane/ethyl acetate = 3:2 v/v) to yield compound **5** (299 mg, 0.410 mmol, 90%) as a yellow solid.

$^1$H NMR (400 MHz, $CDCl_3$): $\delta$ = 0.98–1.08 (m, 21H), 3.56 (t, $J$ = 5.6 Hz, 2H), 3.61–3.67 (m, 6H), 3.68–3.71 (m, 2H), 3.79–3.82 (m, 4H), 4.07 (t, $J$ = 4.8 Hz, 2H), 6.89 (d, $J$ = 8.8 Hz, 2H), 7.58 (d, $J$ = 8.8 Hz, 2H), 7.72 (d, $J$ = 8.4 Hz, 1H), 7.76–7.81 (m, 2H), 7.87 (d, $J$ = 8.0 Hz, 1H), 8.00 (d, $J$ = 8.0 Hz, 1H), 8.01 (d, $J$ = 8.0 Hz, 1H), 8.12 (d, $J$ = 8.8 Hz, 1H), 8.37 (d, $J$ = 9.2 Hz, 1H). $^{13}$C NMR (100 MHz, $CDCl_3$): $\delta$ = 11.91, 17.99, 62.94, 67.41, 69.60, 70.62, 70.71, 70,80, 70.84, 72.73, 87.28, 95.65, 114.71, 115.65, 118.82, 120.18, 123.40, 124.80, 125.04, 125.54, 125.67, 126.05, 127.48, 128.43, 129.37, 129.72, 130.03, 130.17, 130.36, 131.30, 133.10, 158.98. MS (MALDI-TOF): m/z: 730.13 (calcd. $[M]^+$ = 730.25).

**PC-Py1-TIPS**. A mixture of compound **5** (299 mg, 0.410 mmol), *pseudogem*-diethynyl[2.2]paracyclophane (30.0 mg, 0.117 mmol), $Pd(PPh_3)_4$ (60.0 mg, 5.19 × $10^{-2}$ mmol), CuI (10.0 mg, 5.25 × $10^{-2}$ mmol), and *i*-$Pr_2NH$ (8 mL) in THF (20 mL) was stirred for 14 h at 80 ºC. The reaction mixture was poured into $CH_2Cl_2$ (50 mL), and the organic layer was washed with 5% aq. HCl (80 mL), saturated aq. $NaHCO_3$ (50 mL), and brine (50 mL). The organic layer was dried over $MgSO_4$ and filtered before the solvent was evaporated under reduced pressure. The crude product thus isolated was purified by flash column chromatography on silica gel (eluent: from hexane/ethyl acetate = 17:3 v/v to hexane/ethyl acetate = 3:2 v/v) and recycling GPC (eluent: $CHCl_3$) to yield **PC-Py1-TIPS** (92.7 mg, 5.96 × $10^{-2}$ mmol, 51%) as an orange solid.

$^1$H NMR (400 MHz, $CDCl_3$): $\delta$ = 1.06–1.14 (m, 42H), 3.14–3.25 (m, 4H), 3.33–3.37 (m, 2H), 3.60 (t, $J$ = 5.6 Hz, 4H), 3.67–3.72 (m, 12H), 3.75–3.77 (m, 4H), 3.85 (t, $J$ = 5.6 Hz, 4H), 3.89 (t, $J$ = 4.4 Hz, 4H), 4.12 (t, $J$ = 4.4 Hz, 4H), 4.33–4.38 (m, 2H), 6.60 (d, $J$ = 7.6 Hz, 2H), 6.70 (d, $J$ = 7.6 Hz, 2H), 6.80 (d, $J$ = 8.4 Hz, 4H), 7.05 (s, 2H), 7.18 (d, $J$ = 8.0 Hz, 2H), 7.29 (d, $J$ = 9.2 Hz, 2H), 7.47–7.51 (m, 4H), 7.56 (d, $J$ = 8.4 Hz, 4H), 7.59 (d, $J$ = 8.0 Hz, 2H), 7.87 (d, $J$ = 7.6 Hz, 2H), 8.07 (d, $J$ = 8.8 Hz, 2H), 8.23 (d, $J$ = 8.8 Hz, 2H). $^{13}$C NMR (100 MHz, $CDCl_3$): $\delta$ = 12.03, 18.10, 34.35, 35.16, 63.05, 67.51, 69.79, 70.76, 70.85, 70.93, 70.98, 72.85, 87.79, 93.04, 95.22, 95.92, 114.68,

116.13, 118.23, 118.60, 122.77, 123.01, 123.89, 124.00, 124.44, 125.46, 125.61, 126.81, 127.06, 129.08, 129.09, 129.80, 130.12, 130.63, 131.21, 133.24, 133.32, 134.38, 136.26, 139.64, 142.02, 158.80. MS (MALDI-TOF): m/z: 1552.58 (calcd. $[M]^+$ = 1552.78).

**PC-Py1**. A THF solution of tetrabutylammonium fluoride (ca. 1 mol/L, 0.10 mL, 0.10 mol) was added to **PC-Py1-TIPS** (46.0 mg, $2.96 \times 10^{-2}$ mmol) in THF (40 mL). The mixture was then stirred for 2 h at r.t. The reaction mixture was poured into $CHCl_3$ (100 mL), and the organic layer was washed with water (2 × 50 mL). The organic layer was dried over $MgSO_4$ and filtered before the solvent was evaporated under reduced pressure. The crude product thus isolated was purified by reprecipitation from a mixture of $CHCl_3$ and ethyl acetate three times to yield **PC-Py1** (27.2 mg, $2.19 \times 10^{-2}$ mmol, 74%) as an orange solid.
$^1$H NMR (400 MHz, $CDCl_3$, 45 ºC): $\delta$ = 2.67 (br, 2H), 3.17–3.27 (m, 4H), 3.35–3.39 (m, 2H), 3.63 (t, $J$ = 4.4 Hz, 4H), 3.70–3.79 (m, 20H), 3.91 (t, $J$ = 4.8 Hz, 4H), 4.16 (t, $J$ = 4.8 Hz, 4H), 4.35–4.40 (m, 2H), 6.62 (dd, $J$ = 1.6, 8.0 Hz, 2H), 6.72 (d, $J$ = 7.6 Hz, 2H), 6.83 (d, $J$ = 8.8 Hz, 4H), 7.05 (d, $J$ = 2.0 Hz, 2H), 7.19 (d, $J$ = 8.0 Hz, 2H), 7.30 (d, $J$ = 8.8 Hz, 2H), 7.51 (d, $J$ = 8.0 Hz, 2H), 7.52 (d, $J$ = 9.2 Hz, 2H), 7.57 (d, $J$ = 8.8 Hz, 4H), 7.59 (d, $J$ = 7.6 Hz, 2H), 7.89 (d, $J$ = 8.0 Hz, 2H), 8.09 (d, $J$ = 9.2 Hz, 2H), 8.26 (d, $J$ = 9.2 Hz, 2H). $^{13}$C NMR (100 MHz, $CDCl_3$, 45 ºC): $\delta$ = 34.47, 35.03, 61.99, 67.78, 69.95, 70.63, 70.88, 70.93, 71.09, 72.79, 87.95, 93.17, 95.32, 96.09, 114.92, 116.44, 118.40, 118.82, 122.99, 123.24, 123.97, 124.09, 124.63, 125.59, 125.78, 126.94, 127.15, 129.23, 129.97, 130.33, 130.83, 131.41, 133.31, 133.42, 134.45, 136.39, 139.71, 142.11, 158.97. HRMS (ESI-TOF): m/z: 1263.5032 (calcd. $[M+Na]^+$ = 1263.5018).

**Scheme S2**

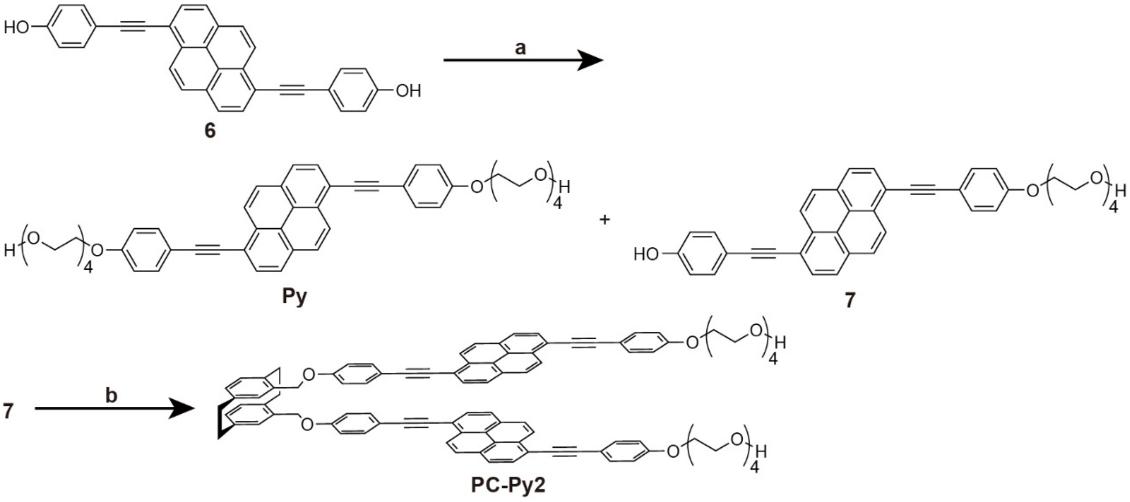


Conditions: (a) compound **1**, $K_2CO_3$, DMF, 13 h, 80 ºC; (b) *pseudogem*-bis(bromomethyl)[2.2]paracyclophane, $K_2CO_3$, DMF, 13 h, 80 ºC.

**Py and compound 7**. Compound **6** (300 mg, 0.690 mmol) was added to a mixture of compound **1** (241 mg, 0.690 mmol) and $K_2CO_3$ (477mg, 3.45 mmol) in DMF (20 mL). The mixture was then stirred for 13 h at 80 ºC. After evaporating the solvent under reduced pressure, the mixture was poured into ethyl acetate (50 mL), and the organic layer was washed with saturated aq. $NH_4Cl$ (4 × 30 mL) and brine (30 mL). The organic layer was dried over $MgSO_4$

and filtered before the solvent was evaporated under reduced pressure. The crude product was purified by flash column chromatography on silica gel (eluent: gradient from $CH_2Cl_2$/acetone = 9:1 v/v to acetone) to afford **Py** (70.2 mg, 8.90 × $10^{-2}$ mmol, 13%) and compound **7** (160 mg, 0.262 mmol, 38%) as yellow solids.

**Py**: $^1$H NMR (400 MHz, $CDCl_3$): $\delta$ = 2.59 (br, 2H), 3.62–3.64 (m, 4H), 3.69–3.78 (m, 20H), 3.91 (t, $J$ = 4.8 Hz, 4H), 4.21 (t, $J$ = 4.8 Hz, 4H), 6.99 (d, $J$ = 8.8 Hz, 4H), 7.65 (d, $J$ = 8.8 Hz, 4H), 8.14–8.17 (m, 4H), 8.20 (d, $J$ = 8.0 Hz, 2H), 8.67 (d, $J$ = 8.8 Hz, 2H). $^{13}$C NMR (100 MHz, $CDCl_3$): $\delta$ = 61.87, 67.57, 69.79, 70.44, 70.71, 70.78, 70.96, 72.59, 87.47, 95.65, 114.88, 115.80, 118.83, 124.36, 125.18, 126.29, 128.13, 129.86, 131.03, 131.95, 133.28, 159.10. HRMS (ESI-TOF): m/z: 809.3294 (calcd. $[M+Na]^+$ = 809.3296).

**Compound 7**: $^1$H NMR (400 MHz, $CDCl_3$): $\delta$ = 2.18 (s, 1H), 3.63–3.65 (m, 2H), 3.73–3.81 (m, 10H), 3.89 (t, $J$ = 4.4 Hz, 2H), 4.07 (t, $J$ = 4.4 Hz, 2H), 6.81 (d, $J$ = 8.8 Hz, 2H), 6.89 (d, $J$ = 8.8 Hz, 2H), 7.14 (s, 1H), 7.50 (d, $J$ = 8.8 Hz, 2H), 7.56 (d, $J$ = 9.2 Hz, 1H), 7.59 (d, $J$ = 8.8 Hz, 2H), 7.75 (d, $J$ = 8.4 Hz, 1H), 7.78–7.82 (m, 2H), 7.86 (d, $J$ = 9.2 Hz, 1H), 8.04 (d, $J$ = 7.6 Hz, 1H), 8.10 (d, $J$ = 8.8 Hz, 1H), 8.49 (d, $J$ = 9.2 Hz, 1H). $^{13}$C NMR (100 MHz, DMSO-$d_6$): $\delta$ = 60.26, 67.39, 68.88, 69.82, 69.86, 69.89, 70.00, 72.41, 86.35, 86.97, 96.00, 96.63, 112.56, 114.36, 115.04, 115.97, 118.36, 123.48, 125.63, 125.70, 128.50, 128.58, 129.79, 129.88, 130.45, 130.60, 130.97, 131.08, 133.28, 133.41, 158.48, 159.10. MS (MALDI-TOF): m/z: 609.40 (calcd. $[M]^+$ = 610.24).

**PC-Py2**. Compound **7** (160 mg, 0.262 mmol) was added to a mixture of *pseudogem*-bis(bromomethyl)[2.2]paracyclophane (34.4 mg, 8.73 × $10^{-2}$ mmol) and $K_2CO_3$ (200 mg, 1.44 mmol) in DMF (20 mL). The mixture was then stirred for 13 h at 80 ºC. After evaporating the solvent under reduced pressure, the mixture was poured into $CHCl_3$ (40 mL), and the organic layer was washed with saturated aq. $NH_4Cl$ (4 × 30 mL) and brine (30 mL). The organic layer was dried over $MgSO_4$ and filtered before the solvent was evaporated under reduced pressure. The crude product was purified by flash column chromatography on silica gel (eluent: gradient from $CH_2Cl_2$ to $CH_2Cl_2$/MeOH = 4:1 v/v), recycling GPC (eluent: chloroform), and reprecipitation from a mixture of $CHCl_3$ and hexane to afford **PC-Py2** (45.4 mg, 3.12 × $10^{-2}$ mmol, 36%) as a yellow solid.

$^1$H NMR (400 MHz, $CDCl_3$): $\delta$ = 2.84 (br, 2H), 3.10–3.14 (m, 2H), 3.17 (s, 4H), 3.46–3.51 (m, 2H), 3.63 (t. $J$ = 4.4 Hz, 4H), 3.71–3.80 (m, 20H), 3.91 (t, $J$ = 4.4 Hz, 4H), 4.17 (t, $J$ = 4.4 Hz, 4H), 4.83 (d, $J$ = 11.6 Hz, 2H), 4.94 (d, $J$ = 11.2 Hz, 2H), 6.62–6.66 (m, 4H), 6.77 (s, 2H), 6.83 (d, $J$ = 8.8 Hz, 4H), 6.88 (d, $J$ = 8.8 Hz, 4H), 7.54 (d, $J$ = 9.2 Hz, 2H), 7.57 (d, $J$ = 8.0 Hz, 2H), 7.61 (d, $J$ = 8.8 Hz, 4H), 7.68–7.70 (m, 8H), 7.87 (d, $J$ = 8.0 Hz, 2H), 7.92 (d, $J$ = 8.0 Hz, 2H), 8.31 (d, $J$ = 9.2 Hz, 2H), 8.32 (d, $J$ = 9.2 Hz, 2H). $^{13}$C NMR (100 MHz, $CDCl_3$): $\delta$ = 31.90, 35.22, 61.84, 67.54, 68.48, 69.79, 70.44, 70.71, 70.79, 70.94, 72.67, 87.69, 87.85, 95.19, 95.35, 114.77, 116.04, 116.18, 118.21, 118.32, 123.51, 123.58, 124.64, 125.58, 125.78, 127.48, 127.53, 129.29, 129.34, 130.35, 130.53, 131.45, 131.61, 131.67, 133.31, 133.39, 133.48, 134.75, 135.22, 136.78, 140.09, 158.89, 158.95. HRMS (ESI-TOF): m/z: 1475.5878 (calcd. $[M+Na]^+$ = 1475.5855).

**Scheme S3**

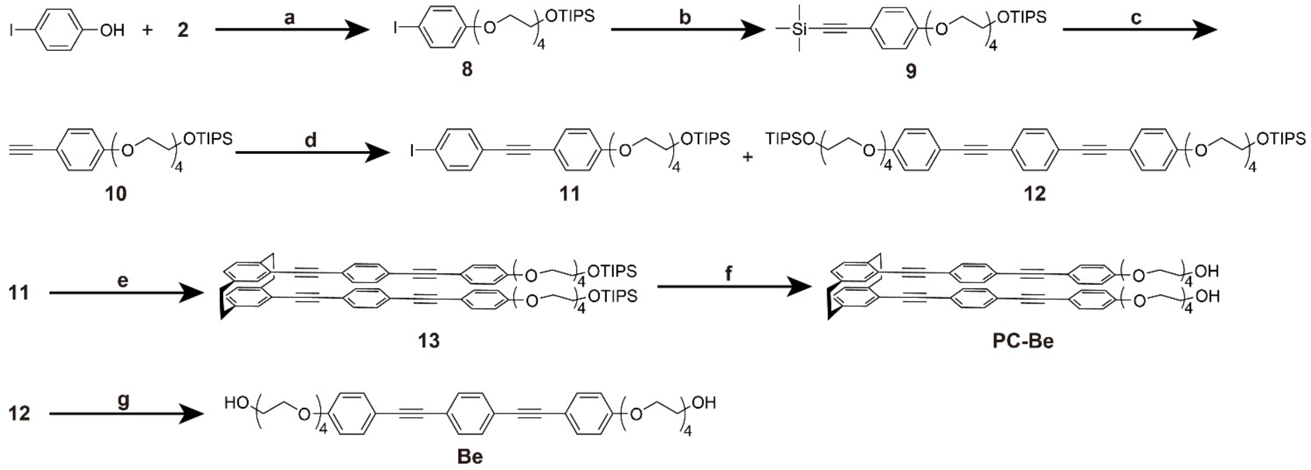


Conditions: (a) $K_2CO_3$, DMF, 15 h, 80 ºC; (b) trimethylsilylacetylene, CuI, $Pd(PPh_3)_4$, $Et_2NH$, THF, 18 h, reflux; (c) $K_2CO_3$, MeOH, 2 h, r.t.; (d) 1,4-diiodobenzene, CuI, $Pd(PPh_3)_4$, *i*-$Pr_2NH$, THF, 13 h, reflux; (e) *pseudogem*-diethynyl[2.2]paracyclophane, $Pd(PPh_3)_4$, CuI, *i*-$Pr_2NH$, THF, 10 h, reflux; (f) tetrabutylammonium fluoride, THF, 1 h, r.t.; (g) tetrabutylammonium fluoride, THF, 1 h, r.t.

**Compound 8**. 4-Iodophenol (3.00 g, 13.6 mmol) was added to a mixture of compound **2** (8.95 g, 17.7 mmol) and $K_2CO_3$ (5.65 g, 40.9 mmol) in DMF (40 mL). After the mixture was stirred for 15 h at 80 ºC, the reaction mixture was poured into ethyl acetate (50 mL), and the organic layer was washed with saturated aq. $NH_4Cl$ (4 × 50 mL) and brine (50 mL). The organic layer was dried over $MgSO_4$ and filtered before the solvent was evaporated under reduced pressure. The crude product thus isolated was purified by flash column chromatography on silica gel (eluent: from hexane/ethyl acetate = 9:1 v/v to hexane/ethyl acetate = 4:1 v/v) to yield compound **8** (5.63 g, 10.2 mmol, 75%) as a colorless liquid.

$^1$H NMR (400 MHz, $CDCl_3$): $\delta$ = 1.03–1.12 (m, 21H), 3.59 (t, $J$ = 5.6 Hz, 2H), 3.63–3.69 (m, 6H), 3.71–3.73 (m, 2H), 3.82–3.86 (m, 4H), 4.09 (t, $J$ = 4.8 Hz, 2H), 6.70 (d, $J$ = 8.8 Hz, 2H), 7.54 (d, $J$ = 8.8 Hz, 2H). $^{13}$C NMR (100 MHz, $CDCl_3$): $\delta$ = 12.00, 18.06, 63.02, 67.57, 69.68, 70.73, 70.81, 70.89, 70.94, 72.81, 83.01, 117.09, 138.23, 158.74. MS (MALDI-TOF): m/z: 574.27 (calcd. $[M+Na]^+$ = 575.17).

**Compound 9.** A mixture of compound **8** (5.63 g, 10.2 mmol), trimethylsilylacetylene (5.00 g, 50.94 mmol), $Pd(PPh_3)_4$ (294 mg, 0.255 mmol), CuI (48.6 mg, 0.255 mmol), and $Et_2NH$ (10 mL) in THF (20 mL) was stirred under reflux for 18 h. The reaction mixture was poured into ethyl acetate (30 mL), and the organic layer was washed with 5% aq. HCl (50 mL), saturated aq. $NaHCO_3$ (50 mL), and brine (50 mL). The organic layer was dried over $MgSO_4$ and filtered before the solvent was evaporated under reduced pressure. The crude product thus isolated was purified by flash column chromatography on silica gel (eluent: from hexane/ethyl acetate = 9:1 v/v to hexane/ethyl acetate = 7:3 v/v) to yield compound **9** (5.32 g, 10.2 mmol, 100 %) as a brown liquid.

$^1$H NMR (400 MHz, $CDCl_3$): $\delta$ = 0.24 (s, 9H), 1.03–1.11 (m, 21H), 3.59 (t, $J$ = 5.6 Hz, 2H), 3.64–3.69 (m, 6H), 3.71–3.74 (m, 2H), 3.82–3.86 (m, 4H), 4.11–4.13 (m, 2H), 6.83 (d, $J$ = 8.8 Hz, 2H), 7.39 (d, $J$ = 8.8 Hz, 2H). $^{13}$C NMR (100 MHz, $CDCl_3$): $\delta$ = 0.18, 12.05, 18.08, 63.06, 67.52, 69.73, 70.76, 70.84, 70.92, 70.98, 72.85, 92.55, 105.28,

114.55, 115.49, 133.54, 159.06. MS (MALDI-TOF): m/z: 544.53 (calcd. $[M+Na]^+$ = 545.31).

**Compound 10**. Compound **9** (5.32 g, 10.2 mmol) was added to a mixture of $K_2CO_3$ (7.16 g, 51.8 mmol) in MeOH (40 mL). After the mixture was stirred for 2 h at r.t., the reaction mixture was poured into ethyl acetate (50 mL). The organic layer was washed with saturated aq. $NH_4Cl$ (2 × 50 mL) and brine (50 mL). The organic layer was dried over $MgSO_4$ and filtered before the solvent was evaporated under reduced pressure. The crude product thus isolated was purified by flash column chromatography on silica gel (eluent: from hexane/ethyl acetate = 19:1 v/v to hexane/ethyl acetate = 2:3 v/v) to yield compound **10** (3.41 g, 7.57 mmol, 73%) as a colorless liquid.

$^1$H NMR (400 MHz, $CDCl_3$): $\delta$ = 1.02–1.14 (m, 21H), 3.00 (s, 1H), 3.59 (t, $J$ = 5.6 Hz, 2H), 3.64–3.70 (m, 6H), 3.72–3.74 (m, 2H), 3.82–3.87 (m 4H), 4.13 (t, $J$ = 4.8 Hz, 2H), 6.85 (d, $J$ = 8.8 Hz, 2H), 7.42 (d, $J$ = 8.8 Hz, 2H). $^{13}$C NMR (100 MHz, $CDCl_3$): $\delta$ = 12.05, 18.10, 63.06, 67.55, 69.74, 70.78, 70.86, 70.95, 71.01, 72.86, 75.95, 83.77, 114.38, 114.68, 133.69, 159.28. MS (MALDI-TOF): m/z: 473.25 (calcd. $[M+Na]^+$ = 473.27).

**Compounds 11 and 12**. A mixture of compound **10** (2.00 g, 4.44 mmol), 1,4-diiodobenzene (1.46 g, 4.44 mmol), $Pd(PPh_3)_4$ (256 mg, 0.222 mmol), CuI (42.0 mg, 0.222 mmol), and *i*-$Pr_2NH$ (10 mL) in THF (30 mL) was stirred under reflux for 13 h. The reaction mixture was poured into ethyl acetate (30 mL), and the organic layer was washed with 5% aq. HCl (50 mL), saturated aq. $NaHCO_3$ (50 mL), and brine (50 mL). The organic layer was dried over $MgSO_4$ and filtered before the solvent was evaporated under reduced pressure. The crude product thus isolated was purified by flash column chromatography on silica gel (eluent: from dichloromethane to dichloromethane/ethyl acetate = 1:1 v/v) to yield compound **11** (994 mg, 1.52 mmol, 34 %) and compound **12** (908 mg, 0.931 mmol, 21%) as pale yellow solids.

**Compound 11**: $^1$H NMR (400 MHz, $CDCl_3$): $\delta$ = 1.01–1.14 (m, 21H), 3.59 (t, $J$ = 5.6 Hz, 2H), 3.64–3.70 (m, 6H), 3.72–3.75 (m, 2H), 3.84 (t, $J$ = 5.6 Hz, 2H), 3.87 (t, $J$ = 4.8 Hz, 2H), 4.15 (t, $J$ = 4.8 Hz, 2H), 6.89 (d, $J$ = 8.8 Hz, 2H), 7.23 (d, $J$ = 8.4 Hz, 2H), 7.44 (d, $J$ = 8.8 Hz, 2H), 7.67 (d, $J$ = 8.4 Hz, 2H). $^{13}$C NMR (100 MHz, $CDCl_3$): $\delta$ = 12.02, 18.08, 63.04, 67.55, 69.72, 70.76, 70.84, 70.92, 70.98, 72.83, 87.33, 90.95, 93.77, 114.77, 115.18, 123.22, 133.05, 133.14, 137.55, 159.13. MS (MALDI-TOF): m/z: 674.73 (calcd. $[M+Na]^+$ = 675.20).

**Compound 12**: $^1$H NMR (400 MHz, $CDCl_3$): $\delta$ = 1.02–1.14 (m, 42H), 3.59 (t, $J$ = 5.6 Hz, 4H), 3.64–3.70 (m, 12H), 3.72–3.75 (m, 4H), 3.82–3.88 (m, 8H), 4.15 (t, $J$ = 4.8 Hz, 4H), 6.89 (d, $J$ = 9.2 Hz, 4H), 7.44–7.46 (m, 8H). $^{13}$C NMR (100 MHz, $CDCl_3$): $\delta$ = 12.03, 18.08, 63.05, 67.56, 69.73, 70.76, 70.83, 70.91, 70.98, 72.84, 88.08, 91.26, 114.78, 115.40, 123.16, 131.42, 133.15, 159.07. MS (MALDI-TOF): m/z: 997.72 (calcd. $[M+Na]^+$ = 997.57).

**Compound 13**. A mixture of compound **11** (229 mg, 0.351 mmol), *pseudogem*-diethynyl[2.2]paracyclophane (30.0 mg, 0.117 mmol), $Pd(PPh_3)_4$ (60.0 mg, $5.19 \times 10^{-2}$ mmol), CuI (10.5 mg, $5.51 \times 10^{-2}$ mmol), and *i*-$Pr_2NH$ (10 mL) in THF (20 mL) was stirred under reflux for 10 h. The reaction mixture was poured into ethyl acetate (30 mL), and the organic layer was washed with 5% aq. HCl (50 mL), saturated aq. $NaHCO_3$ (50 mL), and brine (50 mL). The organic layer was dried over $MgSO_4$ and filtered before the solvent was evaporated under reduced pressure. The crude product thus isolated was purified by flash column chromatography on silica gel (eluent: from dichloromethane to dichloromethane/ethyl acetate = 4:1 v/v) and recycling GPC (eluent: chloroform) to yield compound **13** (81.2 mg,

$6.22 \times 10^{-2}$ mmol, 53 %) as a pale yellow solid.
$^{1}$H NMR (400 MHz, $CDCl_3$): $\delta$ = 1.01–1.14 (m, 42H), 3.06–3.15 (m, 6H), 3.59 (t, $J$ = 5.6 Hz, 4H), 3.64–3.70 (m, 12H), 3.72–3.75 (m, 4H), 3.83–3.87 (m, 8H), 3.96 (dd, $J$ = 12.8, 4.0 Hz, 2H), 4.11 (t, $J$ = 4.8 Hz, 4H), 6.54 (dd, $J$ = 7.6, 1.6 Hz, 2H), 6.60 (d, $J$ = 7.6 Hz, 2H), 6.76 (d, $J$ = 8.8 Hz, 4H), 6.79 (d, $J$ = 1.6 Hz, 2H), 7.18 (d, $J$ = 8.4 Hz, 4H), 7.22 (d, $J$ = 8.4 Hz, 4H), 7.36 (d, $J$ = 8.8 Hz, 4H). $^{13}$C NMR (100 MHz, $CDCl_3$): $\delta$ = 12.00, 18.07, 33.88, 35.01, 63.02, 67.46, 69.72, 70.72, 70.81, 70.89, 70.94, 72.81, 88.26, 90.95, 91.42, 92.98, 114.57, 115.53, 122.76, 123.30, 123.75, 131.04, 131.20, 133.17, 133.23, 134.13, 135.74, 139.37, 142.02, 158.81. MS (MALDI-TOF): m/z: 1305.79 (calcd. $[M]^+$ = 1305.72).

**PC-Be.** A THF solution of tetrabutylammonium fluoride (ca. 1 mol/L, 0.1 mL, 0.1 mmol) was added to compound **13** (39.2 mg, $3.00 \times 10^{-2}$ mmol) in THF (4 mL) and the mixture was stirred for 1 h at r.t. The reaction mixture was poured into ethyl acetate (30 mL), and the organic layer was washed with water (20 mL), and brine (20 mL). The organic layer was dried over $MgSO_4$ and filtered before the solvent was evaporated under reduced pressure. The crude product thus isolated was purified by flash column chromatography on silica gel (eluent: from dichloromethane/acetone = 7:3 v/v to dichloromethane/acetone = 2:3 v/v) and recycling GPC (eluent: chloroform) to yield **PC-Be** (7.21 mg, $7.26 \times 10^{-3}$ mmol, 24 %) as a pale yellow solid.
$^{1}$H NMR (400 MHz, $CDCl_3$): $\delta$ = 2.76 (br, 2H), 3.08–3.15 (m, 6H), 3.61 (t, $J$ = 4.4 Hz, 4H), 3.68–3.76 (m, 20H), 3.87 (t, $J$ = 4.4 Hz, 4H), 3.95 (dd, $J$ = 12.8, 4.0 Hz, 2H), 4.11 (t, $J$ = 4.8 Hz, 4H), 6.54 (dd, $J$ = 8.0, 2.0 Hz, 2H), 6.60 (d, $J$ = 8.0 Hz, 2H), 6.75 (d, $J$ = 8.8 Hz, 4H), 6.79 (d, $J$ = 2.0 Hz, 2H), 7.17 (d, $J$ = 8.8 Hz, 4H), 7.21 (d, $J$ = 8.4 Hz, 4H), 7.35 (d, $J$ = 8.8 Hz, 4H). $^{13}$C NMR (100 MHz, $CDCl_3$): $\delta$ = 33.91, 35.03, 61.80, 67.48, 69.74, 70.39, 70.67, 70.75, 70.89, 72.70, 88.31, 90.93, 91.44, 93.01, 114.59, 115.64, 122.75, 123.33, 123.78, 131.06, 131.22, 133.21, 133.25, 134.15, 135.77, 139.39, 142.05, 158.75. HRMS (ESI-TOF): m/z: 1015.4382 (calcd. $[M+Na]^+$ = 1015.4392).

**Be.** A THF solution of tetrabutylammonium fluoride (ca. 1 mol/L, 0.2 mL, 0.2 mmol) was added to compound **12** (53.2 mg, $5.45 \times 10^{-2}$ mmol) in THF (5 mL) and the mixture was stirred for 1 h at r.t. The reaction mixture was poured into ethyl acetate (30 mL), and the organic layer was washed with water (20 mL), and brine (20 mL). The organic layer was dried over $MgSO_4$ and filtered before the solvent was evaporated under reduced pressure. The crude product thus isolated was purified by flash column chromatography on silica gel (eluent: from dichloromethane/acetone = 1:4 v/v) and recycling GPC (eluent: chloroform) to yield **Be** (9.23 mg, $1.39 \times 10^{-2}$ mmol, 25 %) as a pale yellow solid.
$^{1}$H NMR (400 MHz, $CDCl_3$): $\delta$ = 2.63 (br, 2H), 3.60–3.63 (m, 4H), 3.65–3.76 (m, 20H), 3.87 (t, $J$ = 4.8 Hz, 4H), 4.16 (t, $J$ = 4.8 Hz, 4H), 6.90 (d, $J$ = 8.8 Hz, 4H), 7.44–7.48 (m, 8H). $^{13}$C NMR (100 MHz, $CDCl_3$): $\delta$ = 61.88, 67.54, 69.76, 70.43, 70.70, 70.78, 70.95, 72.63, 88.12, 91.25, 114.78, 115.45, 123.15, 131.45, 133.19, 159.02. HRMS (ESI-TOF): m/z: 685.2974 (calcd. $[M+Na]^+$ = 685.2983).

## NMR analyses

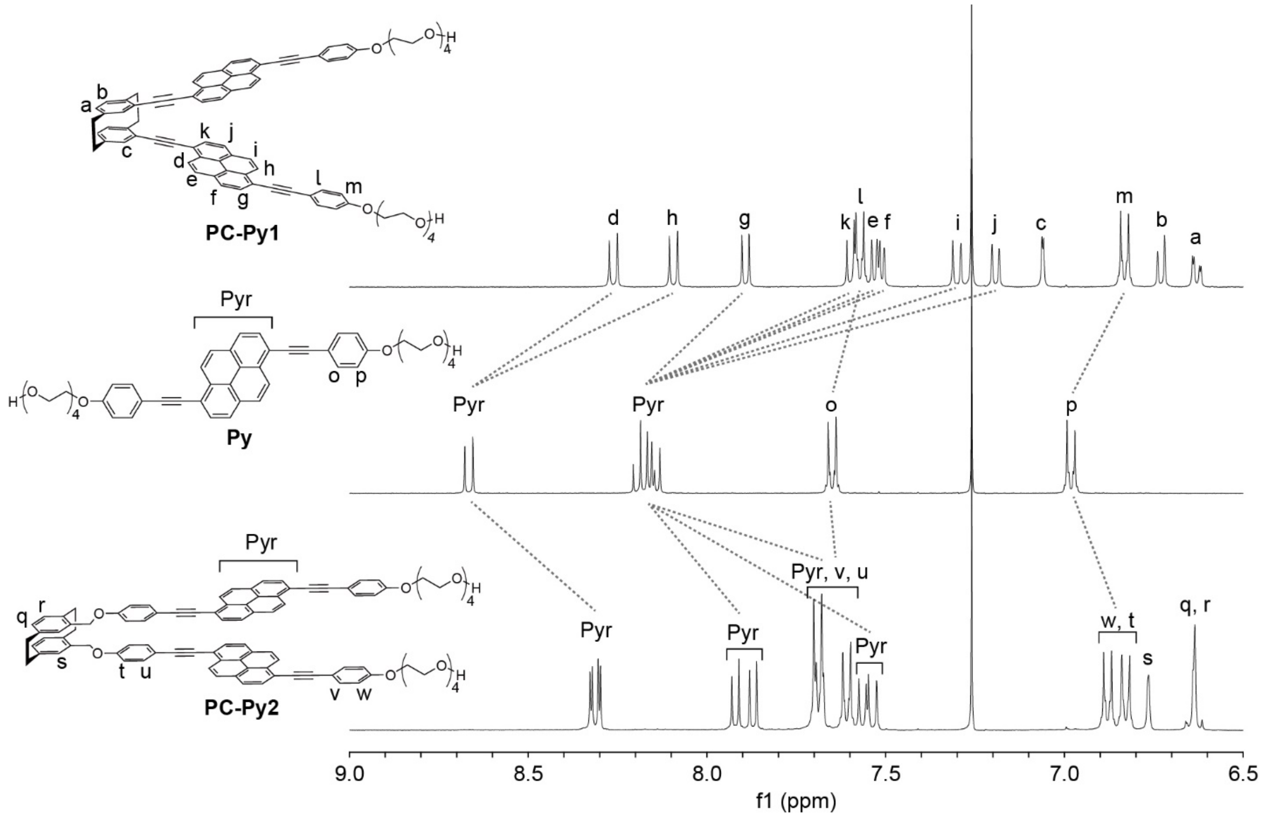


**Figure S1.** Partial $^{1}$H NMR spectra of **PC-Py1** (top), **Py** (middle), and **PC-Py2** (bottom). The spectra were measured in $CDCl_3$ at r.t.

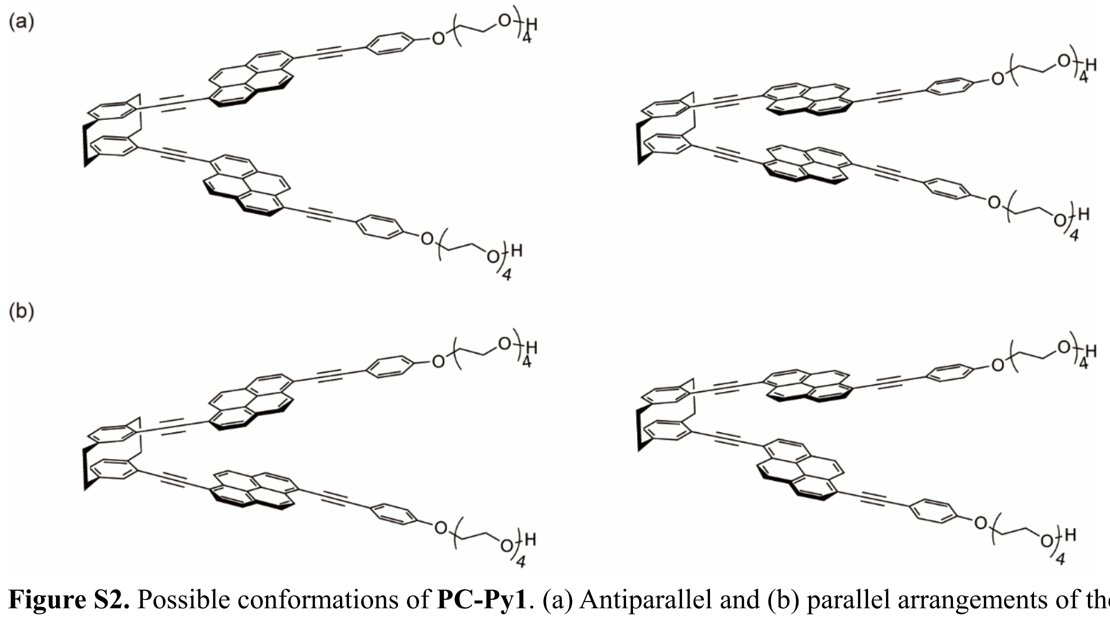


**Figure S2.** Possible conformations of **PC-Py1**. (a) Antiparallel and (b) parallel arrangements of the fluorophores.

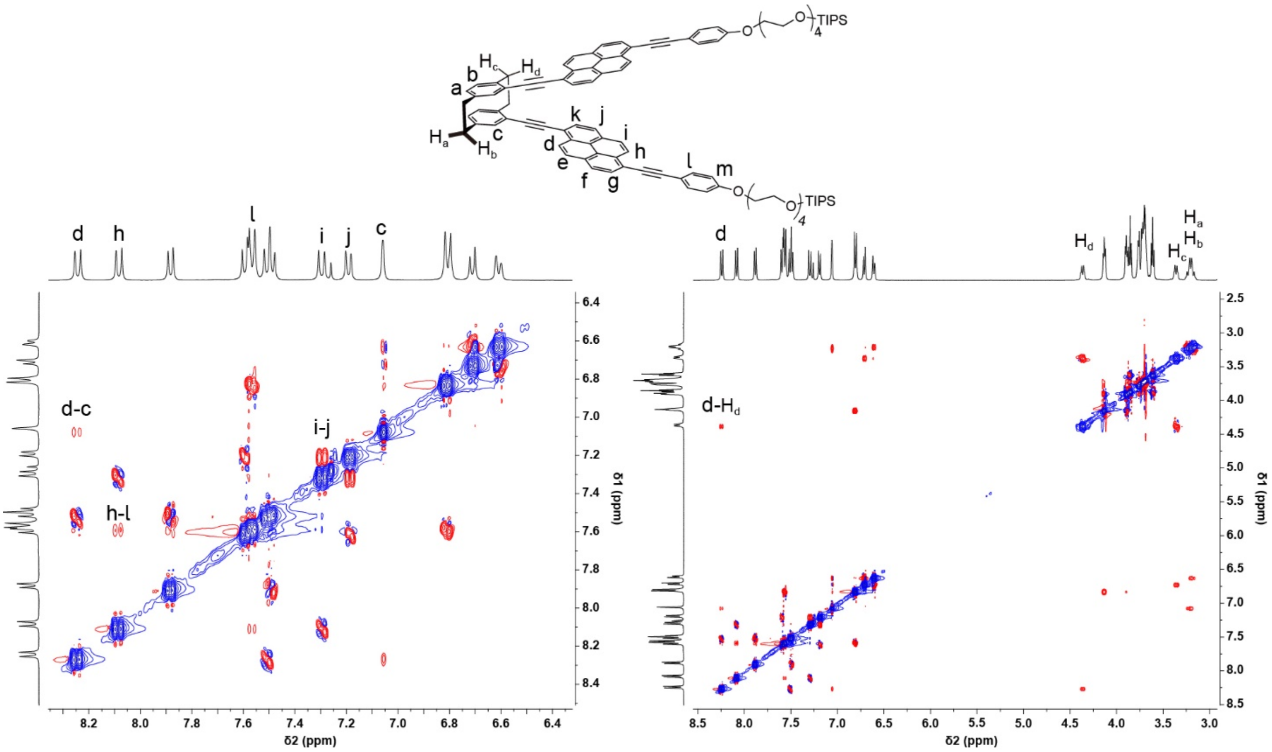


**Figure S3.** Partial $^1$H-$^1$H ROESY NMR spectra of **PC-Py1-TIPS**.

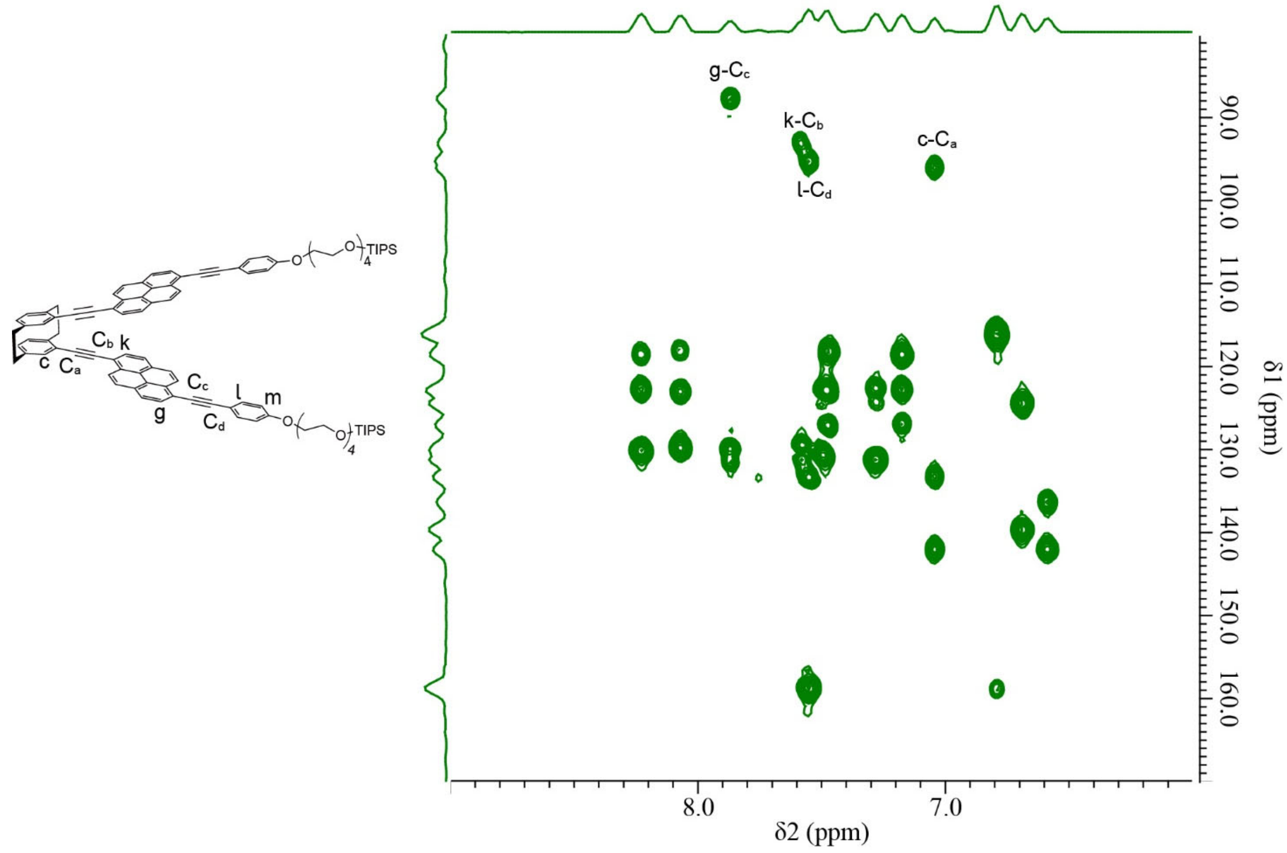


**Figure S4.** Partial $^1$H-$^{13}$C HMBC spectrum of **PC-Py1-TIPS**.

## Emission decay curves of PC-Py1, PC-Py2, and Py in THF

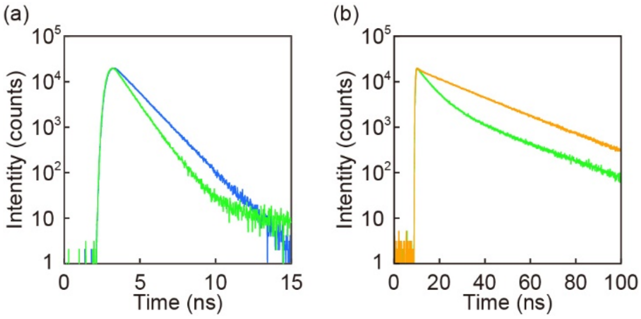


**Figure S5.** (a) Emission decay profiles of **Py** (blue) and **PC-Py2** (green) in THF (monitored at 440 nm). (b) Emission decay profiles of **PC-Py1** (orange) and **PC-Py2** (green) in THF (monitored at 600 nm). The concentrations of the solutions were 1.0 × $10^{-6}$ M and the excitation wavelength was 405 nm.

**Table S1.** Fluorescence lifetimes and relative amplitudes of **PC-Py1**, **PC-Py2**, and **Py** in THF.

| Sample | $\lambda_{mon}$ (nm) | <τ> (ns) | $\tau_1$ (ns) | $\tau_2$ (ns) | $\tau_3$ (ns) | $f_1$ | $f_2$ | $f_3$ | $\chi^2$ |
|---|---|---|---|---|---|---|---|---|---|
| **PC-Py1** | 600 | 22 | 17 | 24 | – | 0.31 | 0.69 | – | 1.01 |
| **PC-Py2** | 600 | 14 | 5.8 | 23 | – | 0.54 | 0.46 | – | 1.09 |
| **PC-Py2** | 440 | 0.91 | 0.39 | 0.93 | 6.4 | 0.13 | 0.86 | 0.0089 | 1.09 |
| **Py** | 440 | 1.2 | 1.2 | – | – | 1.0 | – | – | 1.04 |

$f_n$ is a fraction emission contribution and <τ> is an average lifetime. The values were calculated by using equations (1) and (2), respectively.

$$f_n = \frac{\alpha_n \tau_n}{\alpha_1\tau_1 + \alpha_2\tau_2(+\alpha_3\tau_3)} \qquad (1)$$

$$<\tau> = \frac{\alpha_1\tau_1^2 + \alpha_2\tau_2^2\ (+\alpha_3\tau_3^2)}{\alpha_1\tau_1 + \alpha_2\tau_2\ (+\alpha_3\tau_3)} \qquad (2)$$

, where $\alpha_n$ is an absolute amplitude of the respective emission decaying species.

## Photoluminescence spectra of PC-Py1 and PC-Py2 as a function of concentration

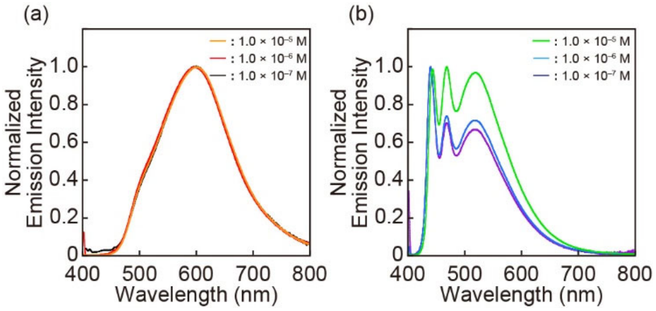


**Figure S6.** Photoluminescence spectra of (a) **PC-Py1** and (b) **PC-Py2** solutions at the indicated concentrations in THF. The spectra were normalized so that the maximum intensities match. $\lambda_{ex}$ = 400 nm.

## Photophysical properties in different solvents

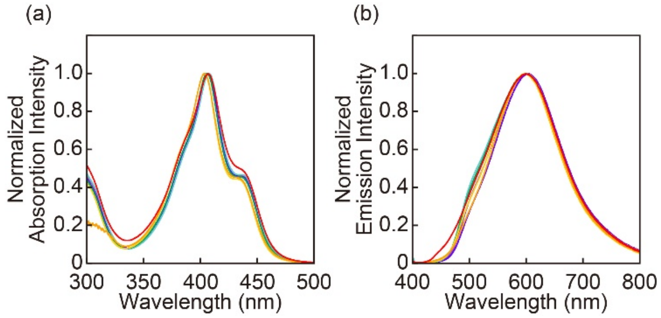


**Figure S7.** (a) UV-vis absorption and (b) photoluminescence spectra of **PC-Py1-TIPS** in chloroform (purple), dichloromethane (blue), toluene (sky blue), tetrahydrofuran (green), ethyl acetate (yellow), acetone (orange), *N*,*N*-dimethylformamide (red). The concentration of **PC-Py1-TIPS** was $1.0 \times 10^{-5}$ M in all solvents, and the excitation wavelength was 400 nm.

## Polymer synthesis

The *x:y* ratios were calculated by $^{1}$H NMR spectroscopy, and the fractions of the mechanophores or reference compounds (*x:z*) were determined based on their concentrations in the monomer feed.

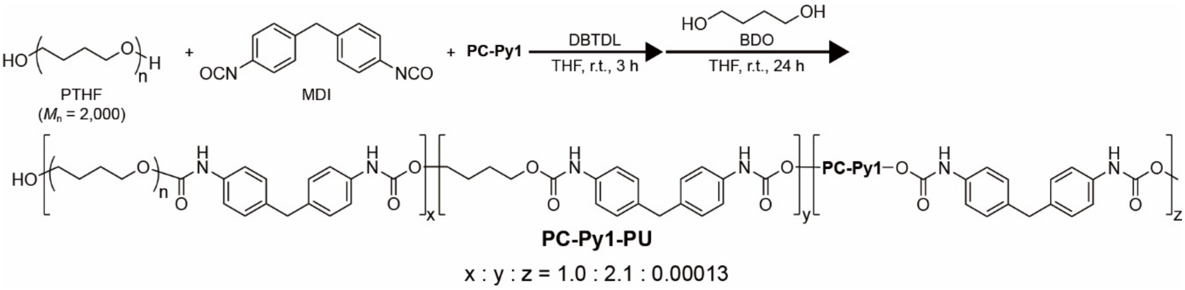


**Synthesis of polyurethane PC-Py1-PU.** Dibutyltin dilaurate (4 drops) was added to a stirred mixture of **PC-Py1** (0.25 mg, 2.0 × $10^{-4}$ mmol), PTHF ($M_n$ = 2,000, 3.00 g, 1.50 mmol), and MDI (1.26 g, 5.03 mmol) in THF (20 mL) and the mixture was stirred at r.t. for 3 h. A solution of BDO (297 mg, 3.30 mmol) in THF (10 mL) was then added, and the reaction mixture was stirred at r.t. for an additional 24 h. After MeOH (10 mL) was then added to the reaction mixture, the solution was stirred for another 30 min, and then poured into MeOH (1,000 mL). The yellow precipitate was collected by filtration and dissolved in THF (150 mL). The resulting solution was filtered through a cotton filter, and the polymer was precipitated into hexane (1,000 mL). The precipitate was filtered off and dried in vacuo for 24 h at r.t. to afford **PC-Py1-PU** as a yellow rubbery solid (3.92 g, 86%, $M_n$ = 125,000, $Đ$ = 2.10).

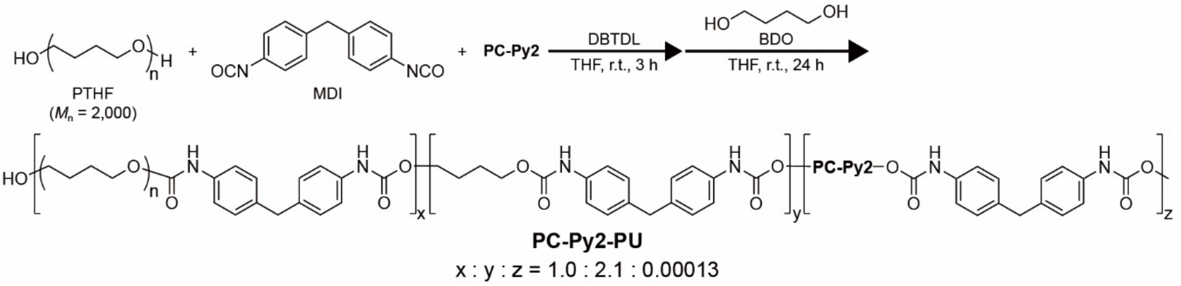


**Synthesis of polyurethane PC-Py2-PU.** Dibutyltin dilaurate (4 drops) was added to a stirred mixture of **PC-Py2** (0.29 mg, 2.0 × $10^{-4}$ mmol), PTHF ($M_n$ = 2,000, 3.00 g, 1.50 mmol), and MDI (1.26 g, 5.03 mmol) in THF (20 mL) and the mixture was stirred at r.t. for 3 h. A solution of BDO (297 mg, 3.30 mmol) in THF (10 mL) was then added, and the reaction mixture was stirred at r.t. for an additional 24 h. MeOH (10 mL) was then added to the reaction mixture, and after stirring for another 30 min, the reaction mixture was poured into MeOH (1,000 mL). The pale-yellow precipitate was collected by filtration and dissolved in THF (150 mL). The resulting solution was filtered through a cotton filter, and the polymer was precipitated into hexane (1,000 mL). The precipitate was filtered off and dried in vacuo for 24 h at r.t. to afford **PC-Py2-PU** as a pale-yellow rubbery solid (3.86 g, 86%, $M_n$ = 123,000, $Đ$ = 2.12).

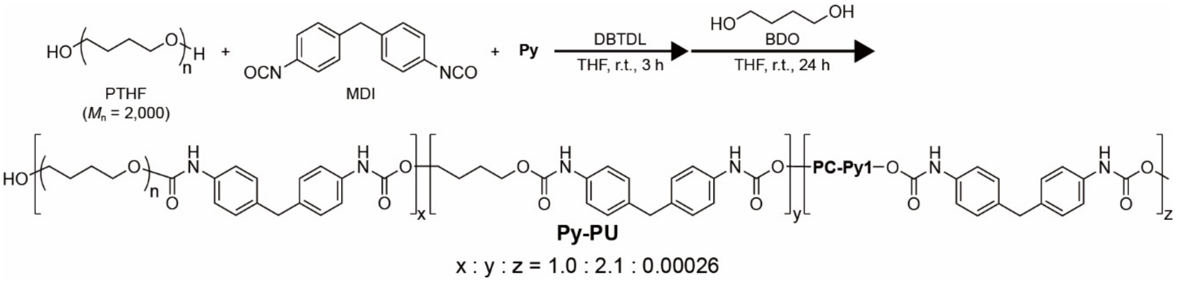

**Synthesis of polyurethane Py-PU.** Dibutyltin dilaurate (4 drops) was added to a stirred mixture of **Py** (0.31 mg, 4.0 × $10^{-4}$ mmol), PTHF ($M_n$ = 2,000, 3.00 g, 1.50 mmol), and MDI (1.26 g, 5.03 mmol) in THF (20 mL) and the mixture was stirred at r.t. for 3 h. A solution of BDO (297 mg, 3.30 mmol) in THF (10 mL) was then added, and the reaction mixture was stirred at r.t. for an additional 24 h. MeOH (10 mL) was then added to the reaction mixture, and after stirring for another 30 min, the reaction mixture was poured into MeOH (1,000 mL). The pale-yellow precipitate was collected by filtration and dissolved in THF (150 mL). The resulting solution was filtered through a cotton filter, and the polymer was precipitated into hexane (1,000 mL). The precipitate was filtered off and dried in vacuo for 24 h at r.t. to afford **Py-PU** as a pale-yellow rubbery solid (3.96 g, 87%, $M_n$ = 142,000, $Đ$ = 2.20).

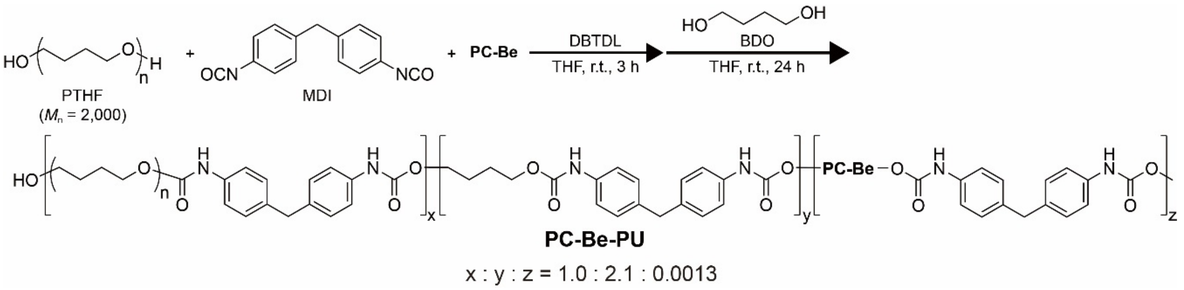

**Synthesis of polyurethane PC-Be-PU.** Dibutyltin dilaurate (4 drops) was added to a stirred mixture of **PC-Be** (2.00 mg, 2.00 × $10^{-3}$ mmol), PTHF ($M_n$ = 2,000, 3.00 g, 1.50 mmol), and MDI (1.29 g, 5.15 mmol) in THF (20 mL) and the mixture was stirred at r.t. for 3 h. A solution of BDO (297 mg, 3.30 mmol) in THF (10 mL) was then added, and the reaction mixture was stirred at r.t. for an additional 24 h. MeOH (10 mL) was then added to the reaction mixture, and after stirring for another 30 min, the reaction mixture was poured into MeOH (1,000 mL). The pale-yellow precipitate was collected by filtration and dissolved in THF (150 mL). The resulting solution was filtered through a cotton filter, and the polymer was precipitated into hexane (1,000 mL). The precipitate was filtered off and dried in vacuo for 24 h at r.t. to afford **PC-Be-PU** as a pale-yellow rubbery solid (3.92 g, 85%, $M_n$ = 96,900, $Đ$ = 2.04).

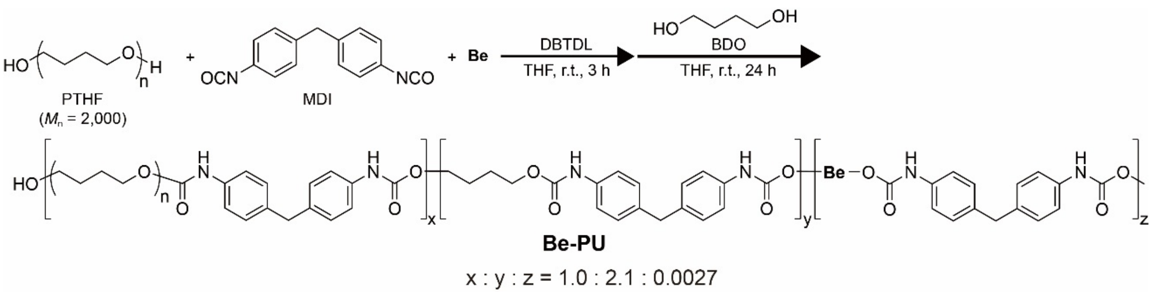

**Synthesis of polyurethane Be-PU.** Dibutyltin dilaurate (4 drops) was added to a stirred mixture of **Be** (2.67 mg, 4.0 × $10^{-3}$ mmol), PTHF ($M_n$ = 2,000, 3.00 g, 1.50 mmol), and MDI (1.29 g, 5.15 mmol) in THF (20 mL) and the mixture was stirred at r.t. for 3 h. A solution of BDO (297 mg, 3.30 mmol) in THF (10 mL) was then added, and the reaction

mixture was additionally stirred at r.t. for an additional 24 h. MeOH (10 mL) was then added to the reaction mixture and, after stirring for another 30 min, the reaction mixture was poured into MeOH (1,000 mL). The pale-yellow precipitate was collected by filtration and dissolved in THF (150 mL). The resulting solution was filtered through a cotton filter, and the polymer was precipitated into hexane (1,000 mL). The precipitate was filtered off and dried in vacuo for 24 h at r.t. to afford **Be-PU** as a pale-yellow rubbery solid (3.89 g, 85%, $M_n$ = 117,000, $Đ$ = 2.08).

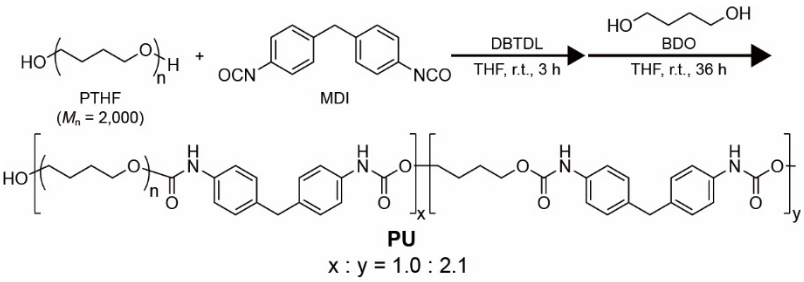


**Synthesis of polyurethane PU.** Dibutyltin dilaurate (4 drops) was added to a stirred mixture of PTHF ($M_n$ = 2,000, 3.00 g, 1.50 mmol), and MDI (1.26 g, 5.03 mmol) in THF (20 mL) and the mixture was stirred at r.t. for 3 h. A solution of BDO (297 mg, 3.30 mmol) in THF (10 mL) was then added, and the reaction mixture was stirred at r.t. for an additional 36 h. MeOH (10 mL) was then added to the reaction mixture, and after stirring for another 30 min, the reaction mixture was poured into MeOH (1,000 mL). The white precipitate was collected by filtration and dissolved in THF (200 mL). The resulting solution was filtered through a cotton filter, and the polymer was precipitated into hexane (900 mL). The precipitate was filtered off and dried in vacuo for 36 h at r.t. to afford **PU** as a white rubbery solid (3.89 g, 85%, $M_n$ = 148,000, $Đ$ = 2.12).

## Preparation of polyurethane films

**Preparation of PC-Py1-PU, PC-Py2-PU, Py-PU, PC-Be-PU, Be-PU, and PU films.** The polyurethanes (**PC-Py1-PU**, **PC-Py2-PU**, **Py-PU**, **PC-Be-PU, Be-PU,** or **PU**, 300 mg) were dissolved in THF (10 mL) and the solutions were poured onto poly(tetrafluoroethylene) molds (35 × 70 × 4 mm). The solutions covered with an inverted funnel were evaporated under ambient conditions for 12 h and in vacuo at r.t. for 6 h. The resulting films were smooth and slightly opaque. The thickness of the films was 70–100 μm.

**Preparation of PC-Py1inPU and PC-Py2inPU films. PC-Py1** (16 μg) or **PC-Py2** (19 μg) was dissolved in THF solution (10 mL) of **PU** (300 mg) and the solutions were poured onto poly(tetrafluoroethylene) molds (35 × 70 × 4 mm). The solutions covered with an inverted funnel were evaporated under ambient conditions for 12 h and in vacuo at r.t. for 6 h. The resulting films were smooth and slightly opaque. The thickness of the films was 80–100 μm.

## $^{1}$H NMR spectra of the polyurethanes

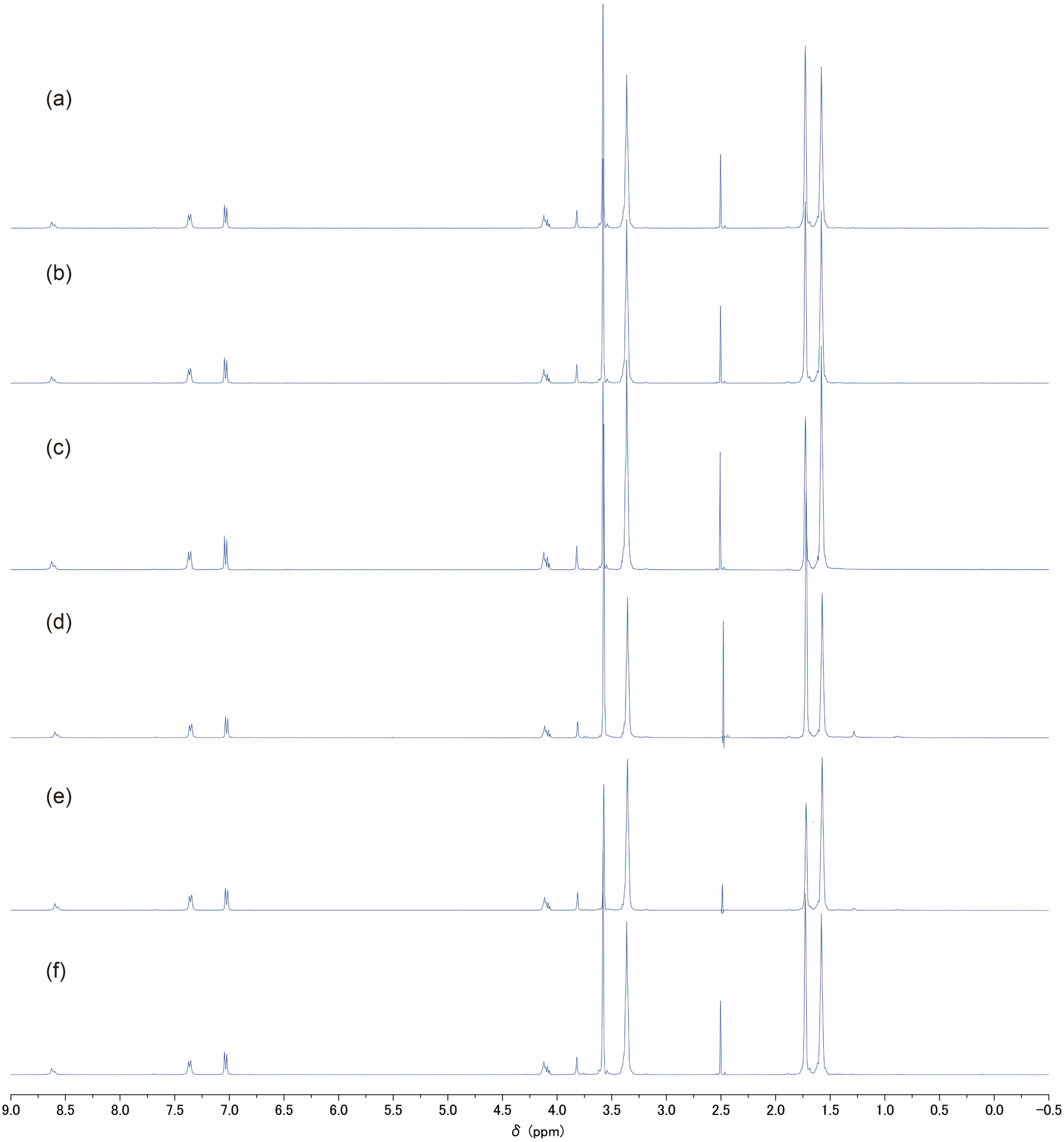


**Figure S8.** $^{1}$H NMR spectra of (a) **PC-Py1-PU**, (b) **PC-Py2-PU**, (c) **Py-PU**, (d) **PC-Be-PU**, (e) **Be-PU**, and (f) **PU** in THF-$d_8$. Signals were characterized as protons of the polyurethane chains. No signals ascribed to the residues of **PC-Py1**, **PC-Py2**, **Py**, **PC-Be**, and **Be** are observable due to their small concentration in **PC-Py1-PU**, **PC-Py2-PU**, **Py-PU**, **PC-Be-PU**, and **Be-PU**, respectively. All spectra were measured at r.t.

## Absorption and photoluminescence spectra of the polyurethane solutions

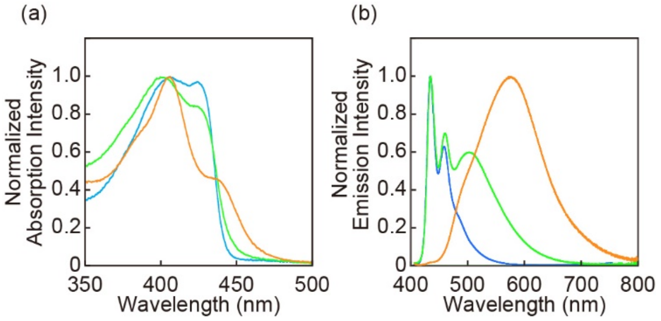


**Figure S9.** (a) UV-vis absorption and (b) photoluminescence spectra of **PC-Py1-PU** (orange), **PC-Py2-PU** (green), and **Py-PU** (blue) in THF ($c$ = 5.0 mg/mL). The spectra were normalized at the maximum intensity. $\lambda_{ex}$ = 400 nm.

## Thermal properties of the polyurethanes

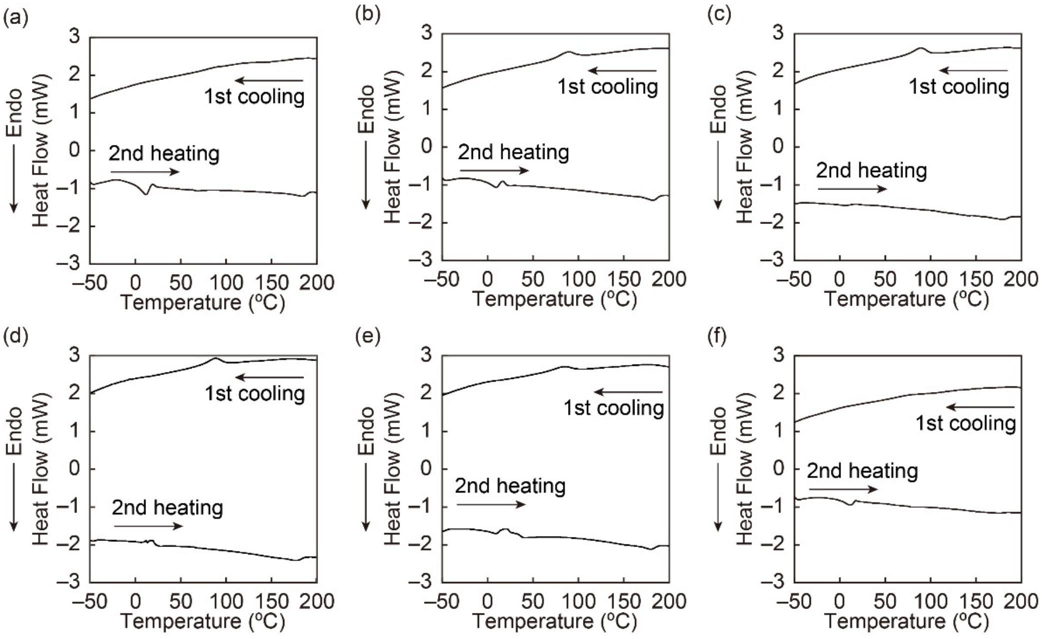


**Figure S10.** DSC traces of (a) **PC-Py1-PU**, (b) **PC-Py2-PU**, (c) **Py-PU**, (d) **PC-Be-PU**, (e) **Be-PU**, and (f) **PU**. Shown are the first cooling and second heating curves. The heating and cooling rates were 10 °C/min.

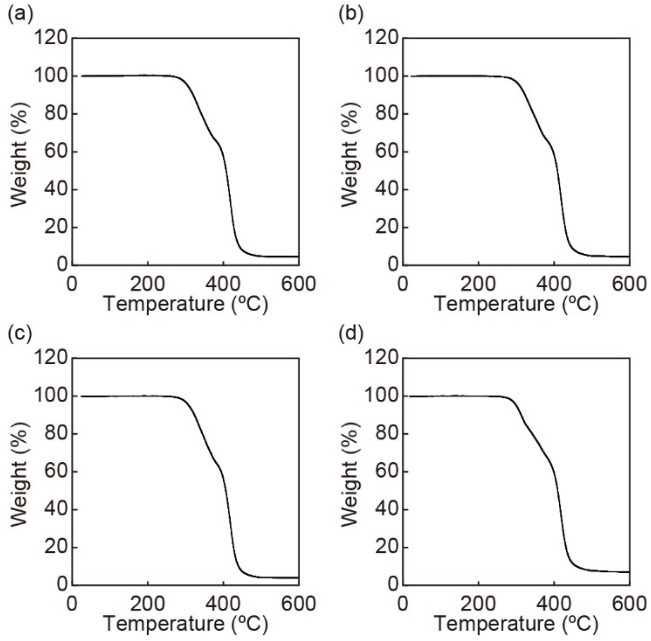


**Figure S11.** TGA traces of (a) **PC-Py1-PU**, (b) **PC-Py2-PU**, (c) **Py-PU**, and (d) **PU**. The heating rate was 10 °C/min.

## Mechanical properties of the polyurethane films

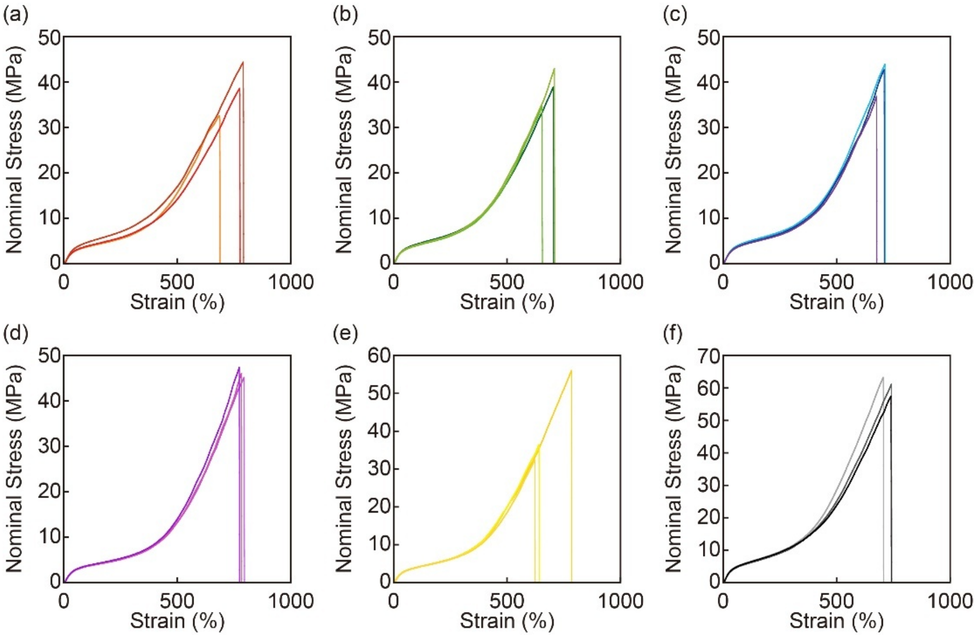


**Figure S12.** Stress-strain curves of (a) **PC-Py1-PU**, (b) **PC-Py2-PU**, (c) **Py-Pu**, (d) **PC-Be-PU**, (e) **Be-PU**, and (f) **PU** films. The tensile tests were carried out at a strain rate of 0.2/s.

**Table S2.** Mechanical data of the polyurethane films extracted from the tensile test data.[a)]

| Sample | Elongation at break (%) | Stress at break (MPa) | Young's modulus[b)] (MPa) |
|---|---|---|---|
| **PC-Py1-PU** | 752 ± 64 | 39 ± 6 | 4.7 ± 1.4 |
| **PC-Py2-PU** | 689 ± 35 | 39 ± 4 | 7.6 ± 2.7 |
| **Py-PU** | 760 ± 51 | 45 ± 2 | 5.8 ± 1.4 |
| **PC-Be-PU** | 783 ± 8.9 | 55 ± 1 | 4.0 ± 0.7 |
| **Be-PU** | 707 ± 107 | 51 ± 16 | 5.1 ± 2.3 |
| **PU** | 727 ± 25 | 43 ± 2 | 6.1 ± 1.6 |

[a)]All data were extracted from the stress-strain curves shown in Figure S11 and represent averages of 3 measurements ± standard deviation. [b)]The Young's moduli were derived from the slopes of the stress-strain curves in the strain regime of 0.5–5%.

## Images of the PC-Py1-PU, PC-Py2-PU, and Py-PU films upon uniaxial deformation

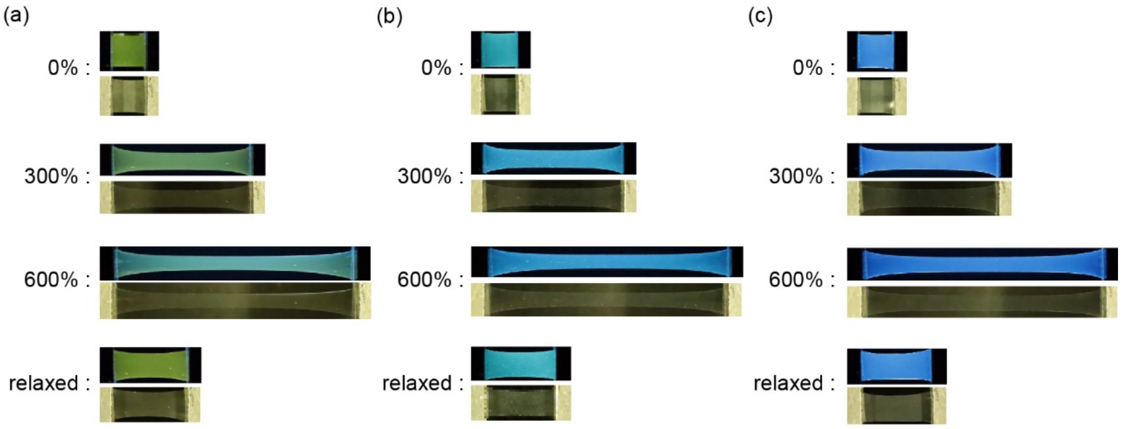


**Figure S13.** Pictures of (a) **PC-Py1-PU**, (b) **PC-Py2-PU**, and (c) **Py-PU** films taken before, during, and after first uniaxial deformation tests. In each photograph, the top images show the photoluminescence of the film taken in the dark with excitation light at 365 nm. The below images were taken under room light. All images were taken under ambient conditions at the indicated strains.

## Absence of mechanochromic behavior of the Py-PU film upon stretching

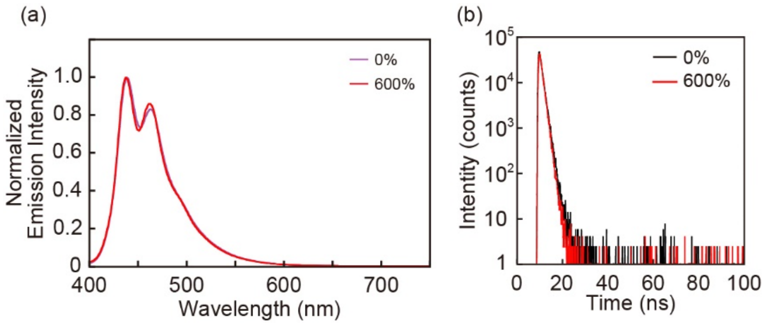


**Figure S14.** (a) Emission spectra and (b) emission decay profiles of a **Py-PU** film in the initial state and at 600% strain. The excitation wavelength was 365 and 405 nm, respectively. The decay profiles were monitored at 500 nm.

## Emission spectra of the polyurethane films and solutions

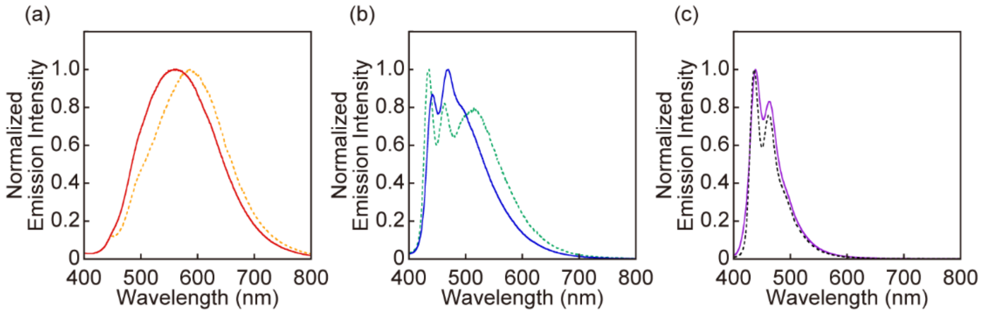


**Figure S15.** Emission spectra of the films (solid lines) and THF solutions ($c$ = 5.0 mg/mL) (dotted lines) of (a) **PC-Py1-PU**, (b) **PC-Py2-PU**, and (c) **Py-PU**. $\lambda_{ex}$ = 365 nm.

## Emission decay profiles of the PC-Py1-PU and PC-Py2-PU films

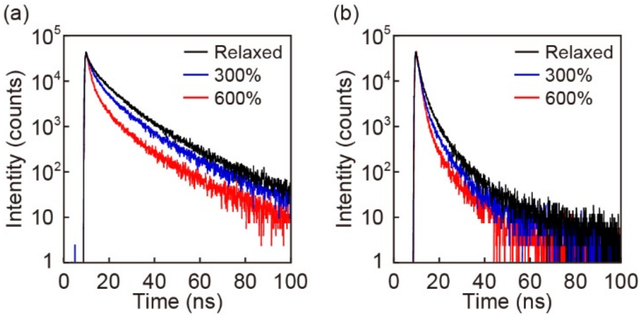


**Figure S16.** Emission decay profiles for (a) **PC-Py1-PU** and (b) **PC-Py2-PU** films on the first relaxation at the strain indicated in the panel. The excitation wavelength was 405 nm, and all profiles were monitored at 500 nm.

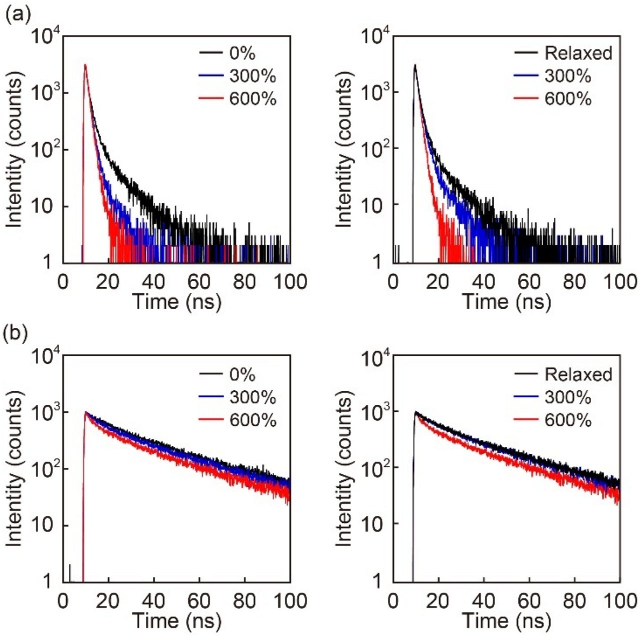


**Figure S17.** Emission decay profiles for **PC-Py1-PU** films on the first stretching (left) and relaxation (right) cycle. The profiles were monitored at (a) 440 and (b) 600 nm. $\lambda_{ex}$ = 405 nm.

## Overlays of true stress-strain curves and emission ratios

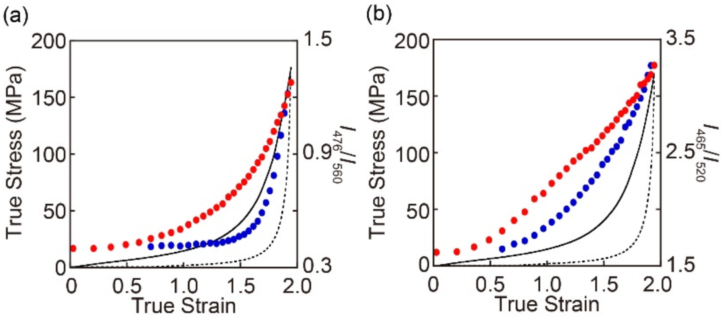


**Figure S18.** Overlays of true stress-true strain curves (stretching: solid lines, relaxing: dotted lines) and relative monomer to excimer emission intensity $I_{476}/I_{560}$ for (a) a **PC-Py1-PU** film and $I_{465}/I_{520}$ for (b) a **PC-Py2-PU** film (stretching: red circles, relaxing: blue circles).

## Plots of the emission ratio against the true stress

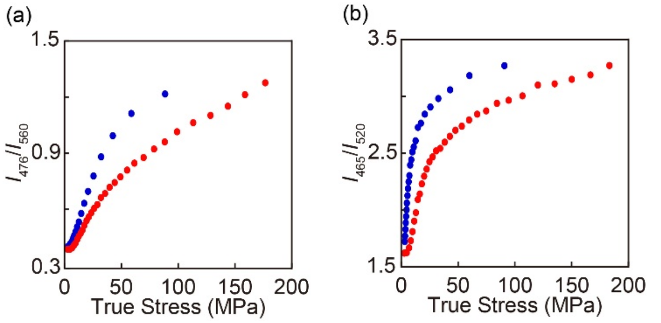


**Figure S19.** Plots of the $I_{476}/I_{560}$ and $I_{465}/I_{520}$ ratios upon the first stretching and relaxing (a) **PC-Py1-PU** and (b) **PC-Py2-PU** films, respectively (stretching: red circles, relaxing: blue circles). The data were taken from Figure 6 and re-plotted.

## Mechanochromic response in the 51st stretch and release cycle

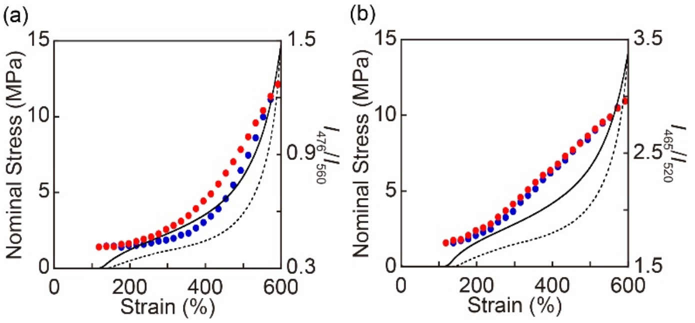


**Figure S20.** Overlays of stress-strain curves (stretching: solid lines, relaxing: dotted lines) and relative monomer to excimer emission intensity $I_{476}/I_{560}$ for (a) a **PC-Py1-PU** film and $I_{465}/I_{520}$ for (b) a **PC-Py2-PU** film (stretching: red circles, relaxing: blue circles) in the 51st stretching and relaxing cycle.

## Photophysical properties of PC-Be and Be

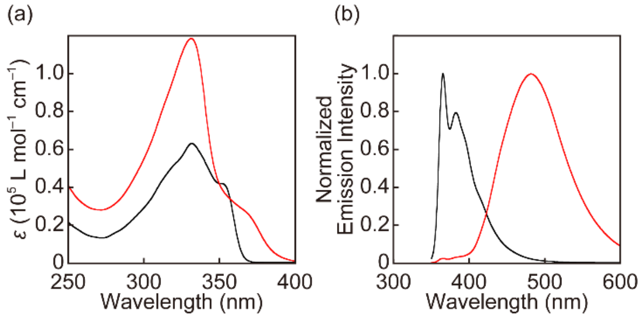


**Figure S21.** (a) UV-vis absorption and (b) photoluminescence spectra of **PC-Be** (red) and **Be** (black) in THF ($c$ = $1.0 \times 10^{-5}$ M). $\lambda_{ex}$ = 330 nm.

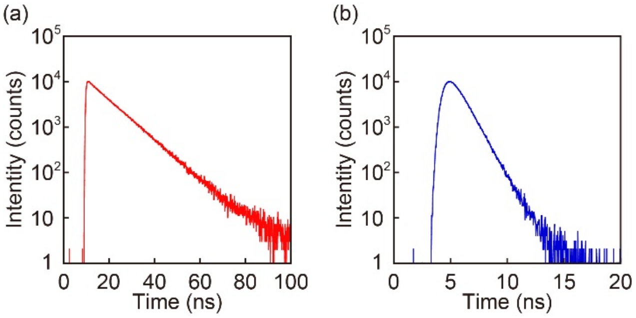


**Figure S22.** Emission decay profiles of (a) **PC-Be** (red) and (b) **Be** (blue) in THF monitored at 480 and 365 nm, respectively. The concentrations of the solutions were $1.0 \times 10^{-5}$ M and the excitation wavelength was 340 nm.

## Mechanochromic behavior of PC-Be-PU and Be-PU films

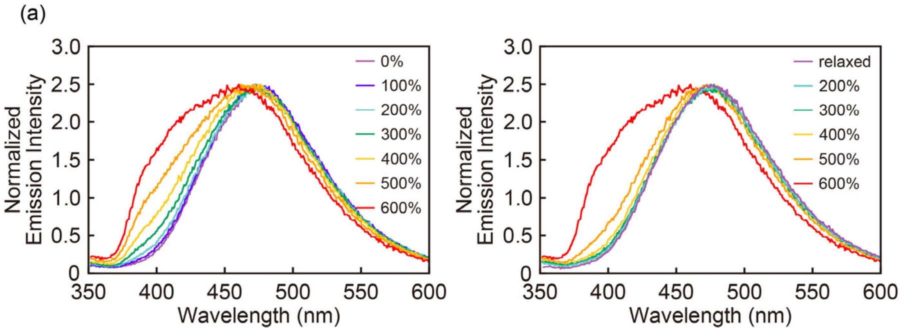


**Figure S23.** Photoluminescence spectra of a **PC-Be-PU** film acquired upon the first stretching (left) and relaxing (right). All spectra were normalized to the maximum intensity. $\lambda_{ex}$ = 310 nm.

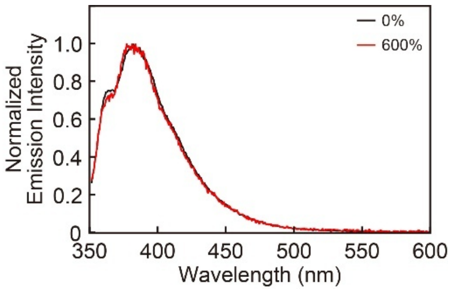


**Figure S24.** Photoluminescence spectra of a **Be-PU** film at 0% and 600% strain upon the first stretching. The two spectra were normalized to the maximum intensity. $\lambda_{ex}$ = 310 nm.

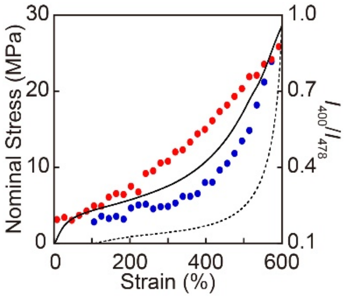


**Figure S25.** Overlays of stress-strain curves (stretching: solid lines, relaxing: dotted lines) and relative monomer to excimer emission intensity $I_{400}/I_{478}$ for a **PC-Be-PU** film (stretching: red circles, relaxing: blue circles) in the first stretching and relaxing cycle.

## Mechanochromic behavior of PC-Py1inPU and PC-Py2inPU films

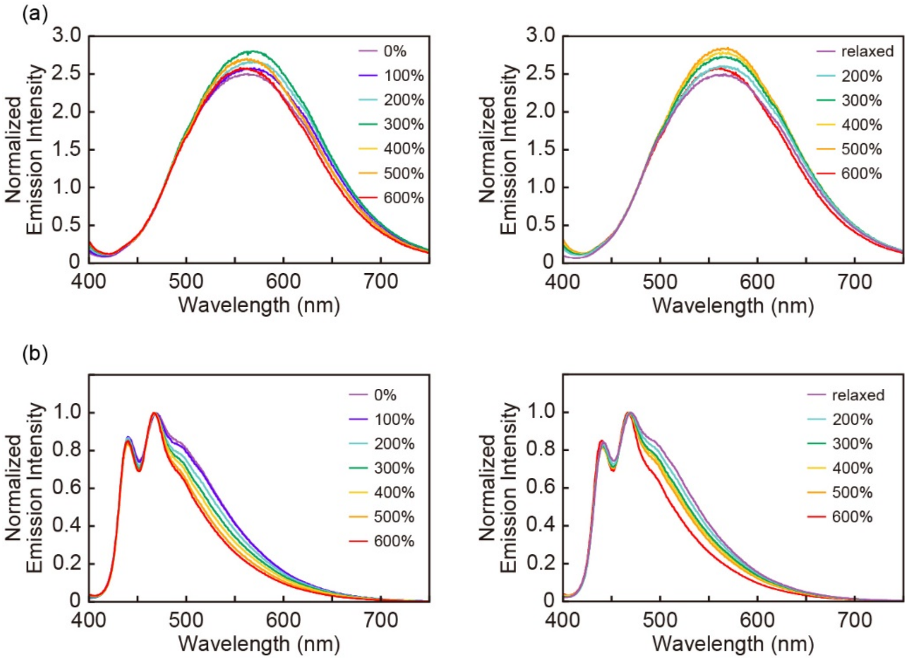


**Figure S26.** Photoluminescence spectra of (a) **PC-Py1inPU** and (b) **PC-Py2inPU** films acquired upon the first stretching (left) and relaxing (right) of the films. All spectra were normalized to the intensity at 476 and 466 nm, respectively. $\lambda_{ex}$ = 365 nm.

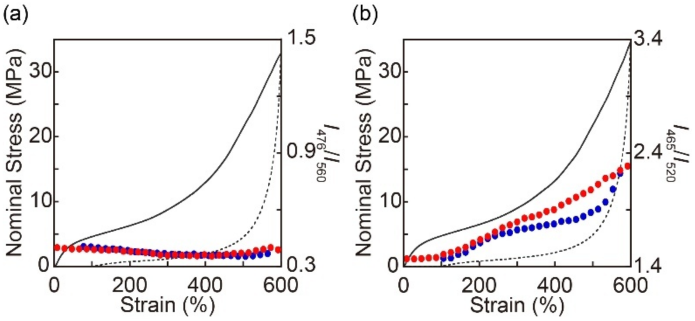


**Figure S27.** Overlays of stress-strain curves (stretching: solid lines, relaxing: dotted lines) and relative monomer to excimer emission intensity recorded for (a) a **PC-Py1inPU** film and for (b) a **PC-Py2inPU** film (stretching: red circles, relaxing: blue circles).

## DFT calculations

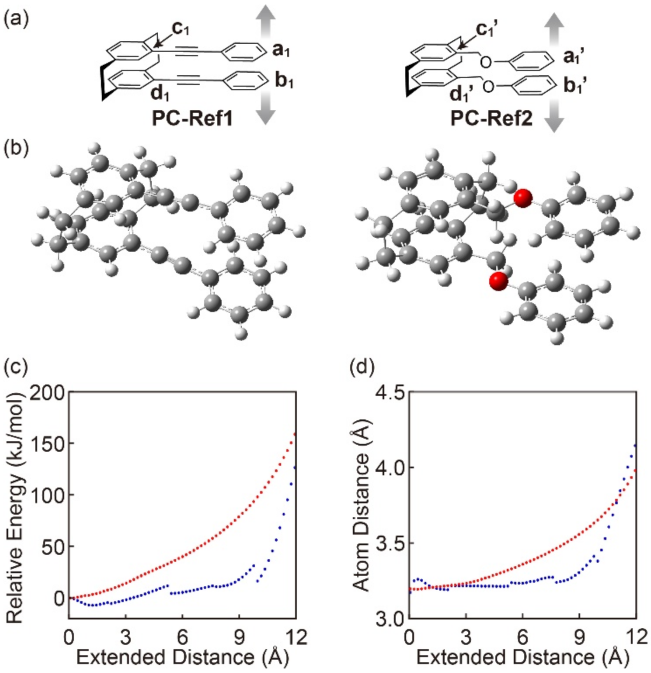


**Figure S28.** (a) Molecular structures of **PC-Ref1** and **PC-Ref2**. (b) Optimized structures of **PC-Ref1** and **PC-Ref2** model molecules. The calculation of **PC-Ref1** was performed under unconstrained condition. The calculation for **PC-Ref2** was conducted to ensure that the distance between carbons $a_1$' and $b_1$' is equal to the distance between carbons $a_1$ and $b_1$. Plots of (c) relative energy and (d) atom distance between carbon atoms [$c_1$,$d_1$] and [$c_1$',$d_1$'] against the extended distances between carbon atoms [$a_1$,$b_1$] and [$a_1$',$b_1$'], respectively (**PC-Ref1**: red dots, **PC-Ref2**: blue dots), when the distances between carbons [$a_1$,$b_1$] and [$a_1$',$b_1$'] are extended. These results were obtained from DFT calculations using CoGEF method at the level of theory with CAM-B3LYP/6-31+G(d,p).

**NMR spectra**

$^{1}$H NMR spectrum of compound **2**

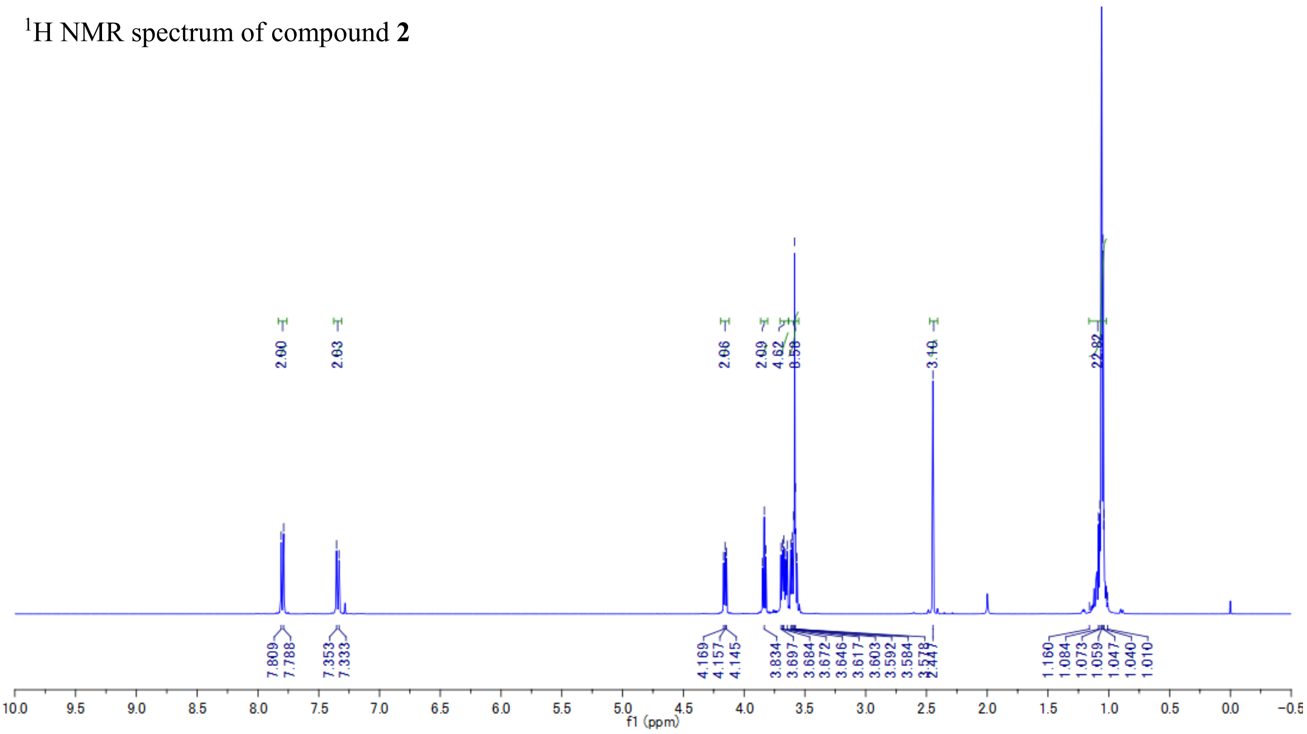

$^{13}$C NMR spectrum of compound **2**

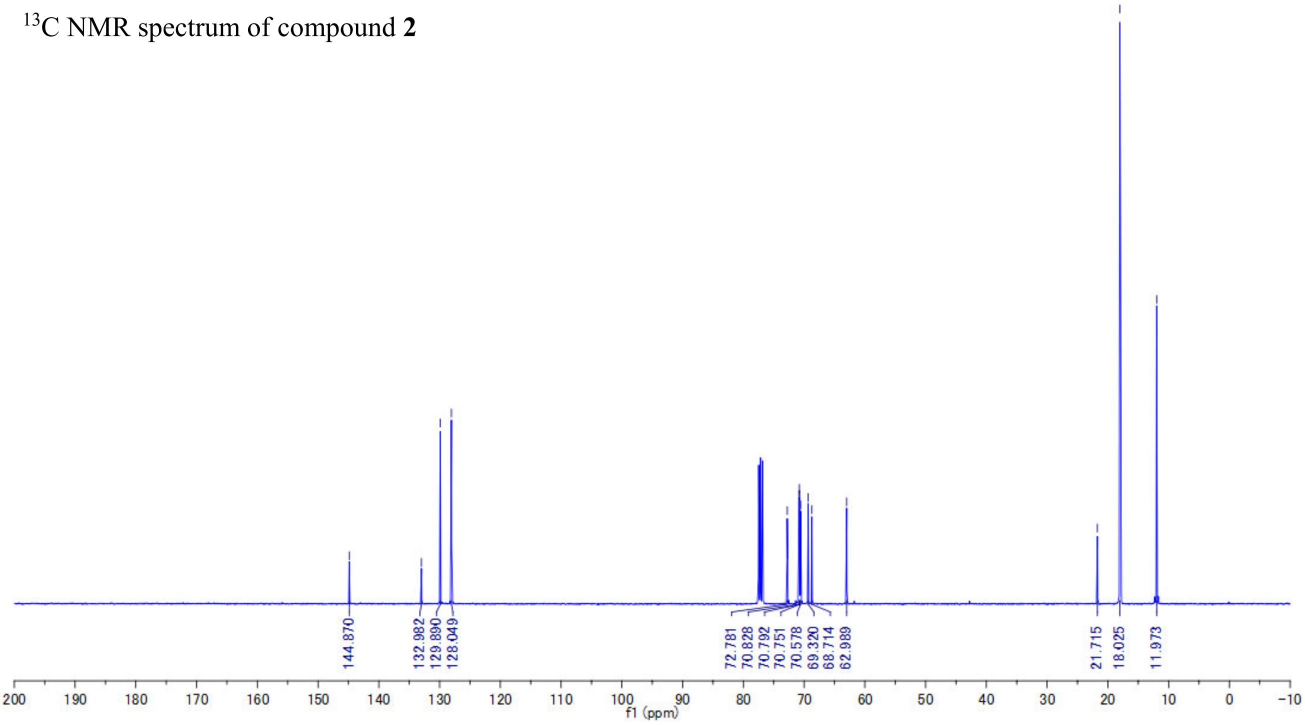

$^{1}$H NMR spectrum of compound **4**

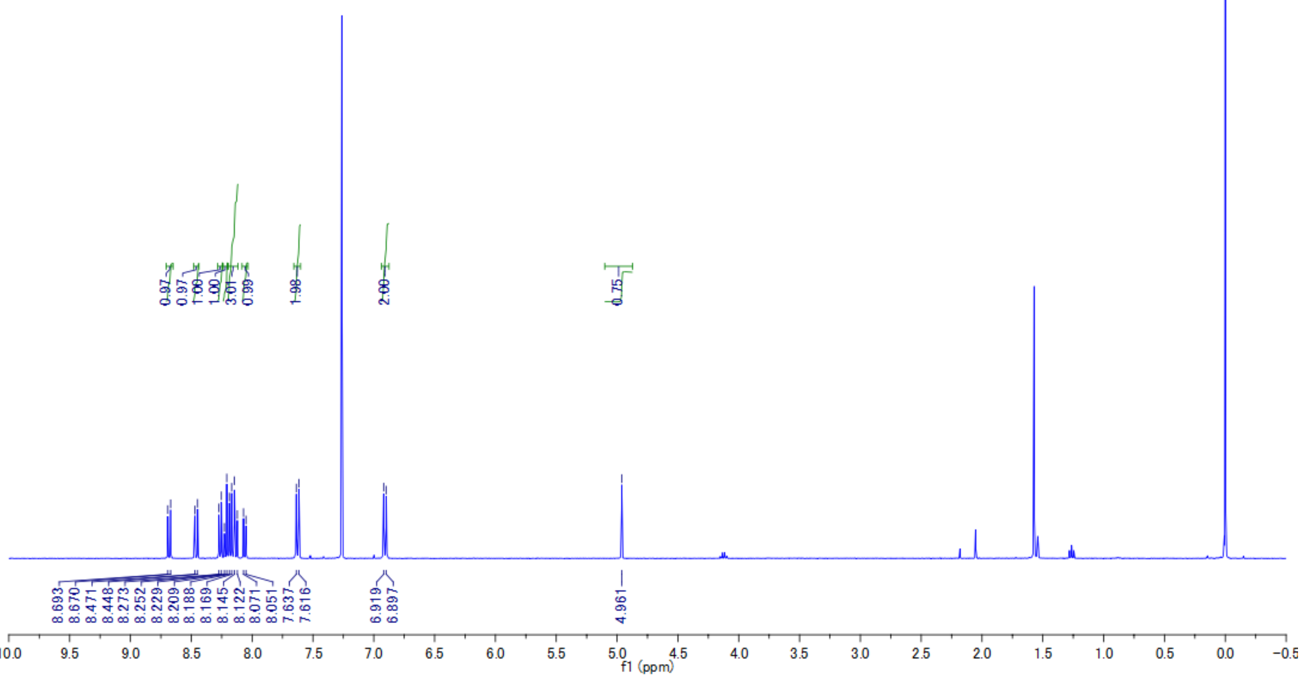


$^{13}$C NMR spectrum of compound **4**

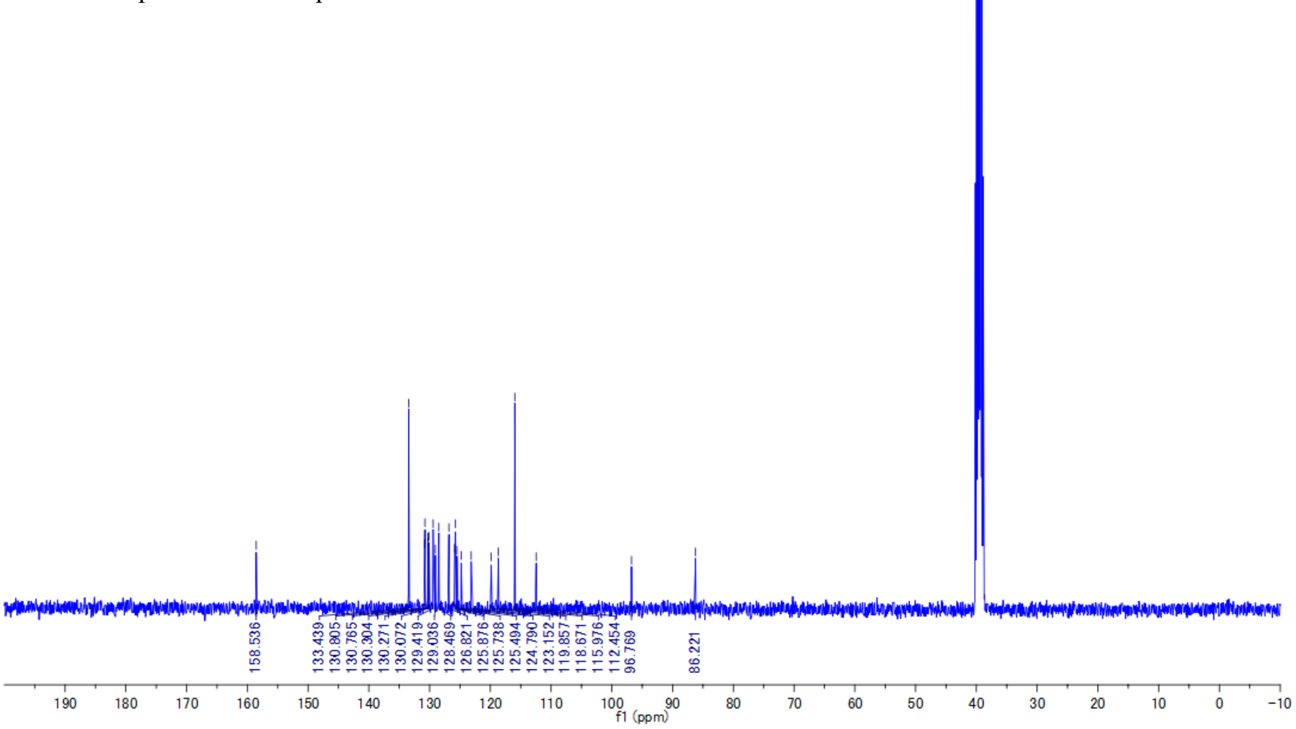

$^{1}$H NMR spectrum of compound **5**

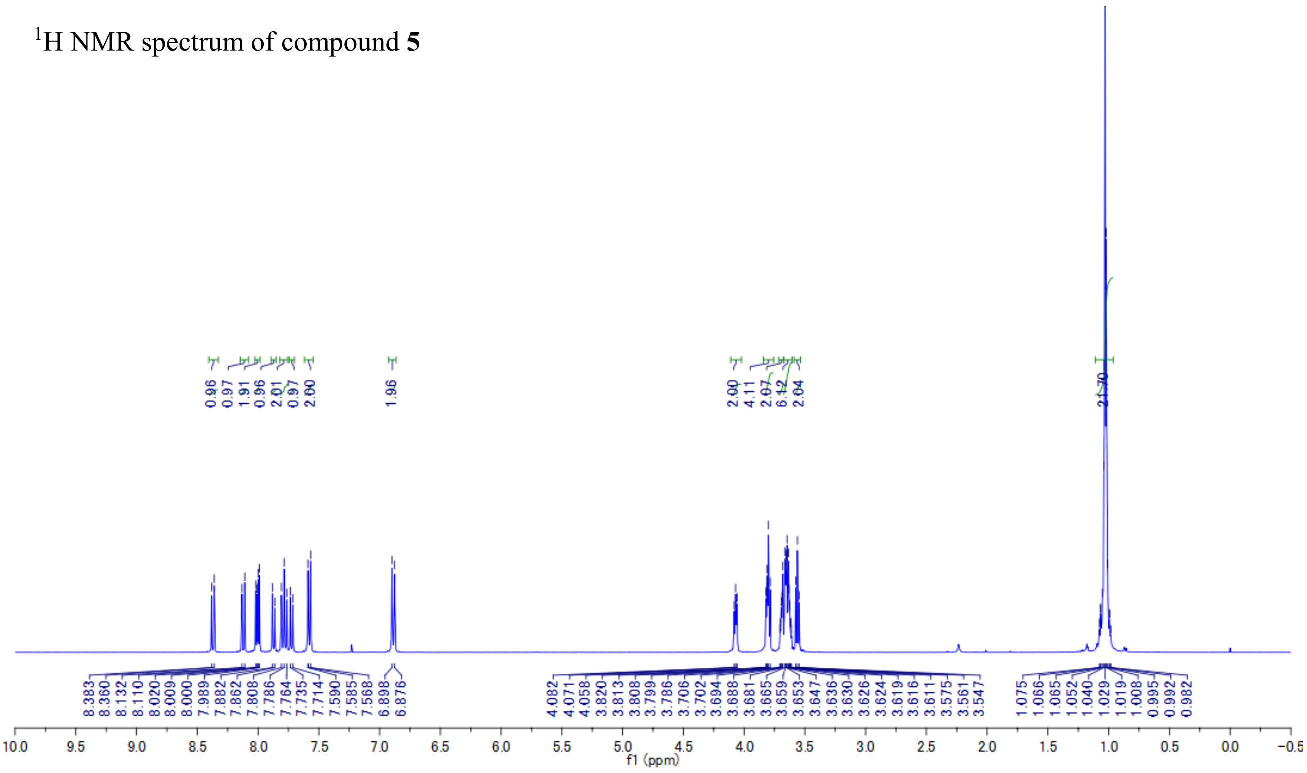


$^{13}$C NMR spectrum of compound **5**

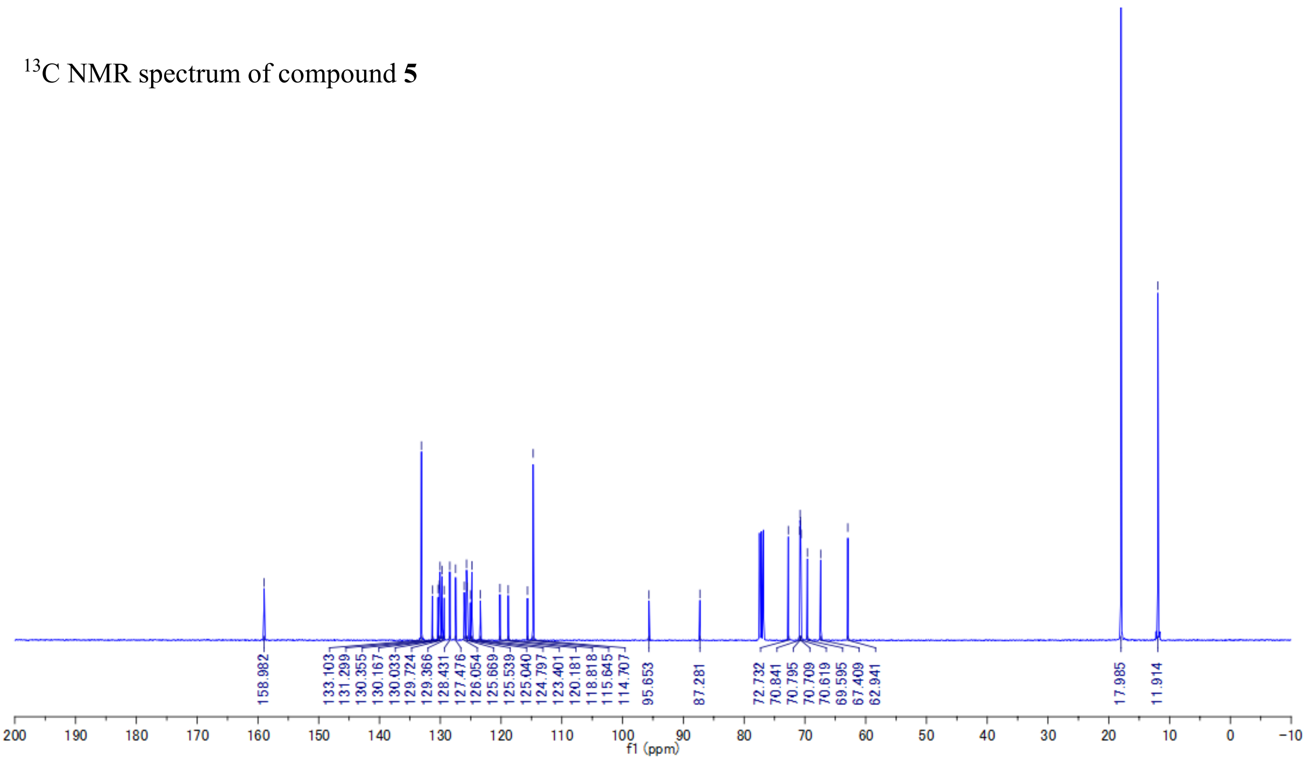

$^{1}$H NMR spectrum of **PC-Py1-TIPS**

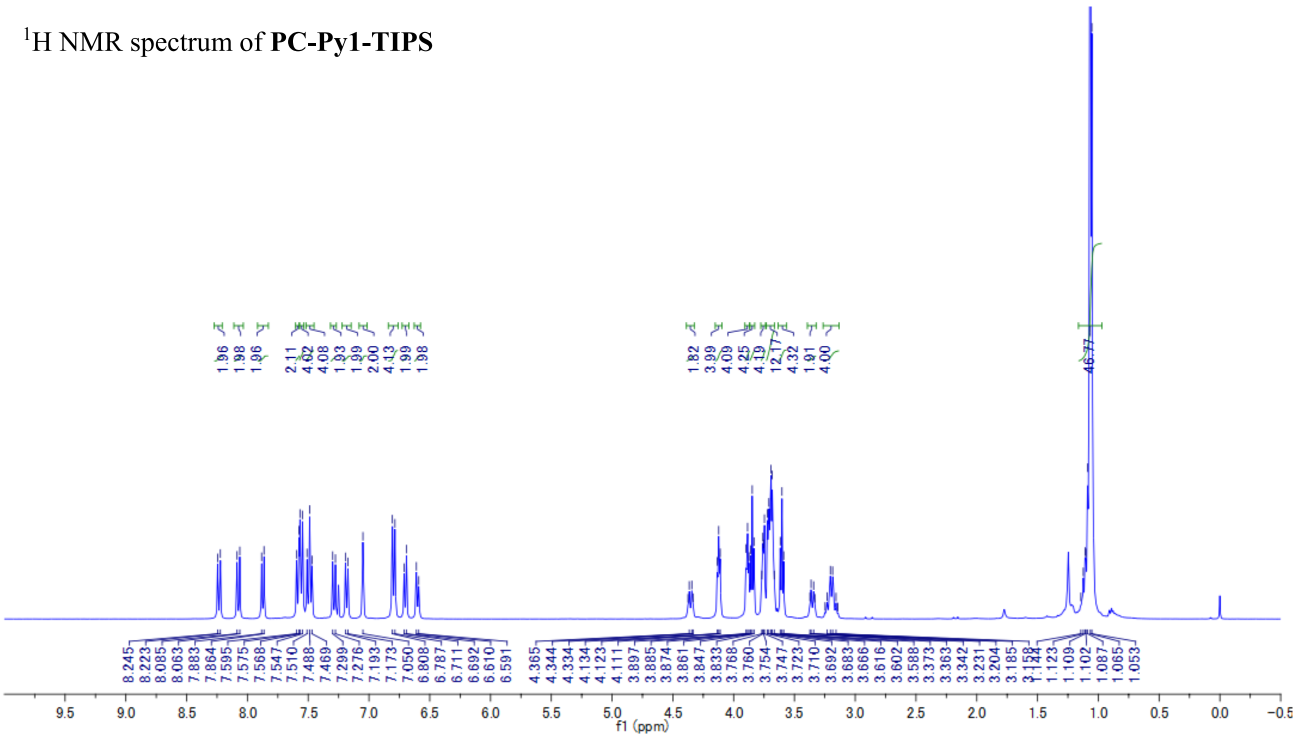

$^{13}$C NMR spectrum of **PC-Py1-TIPS**

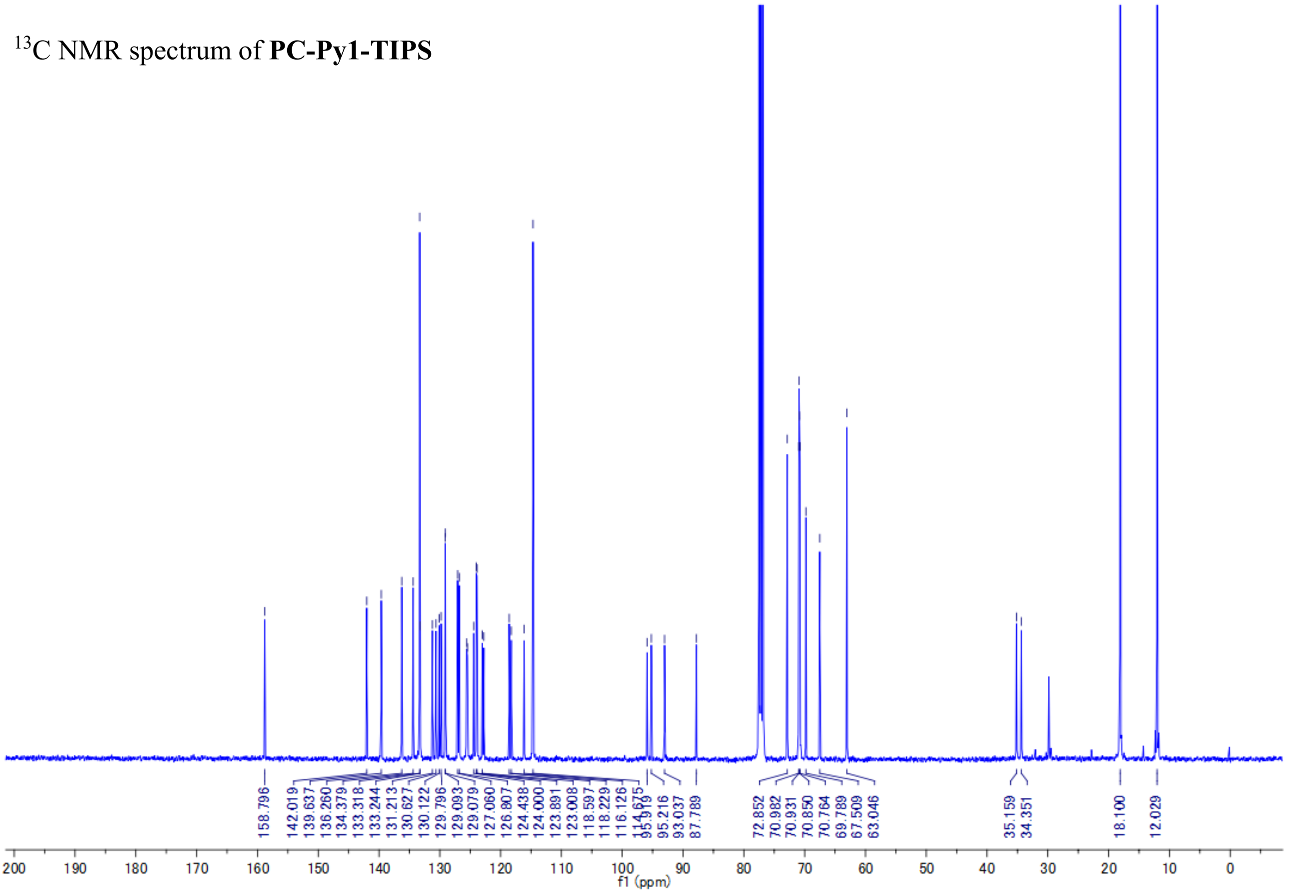

$^{1}$H-$^{1}$H COSY NMR spectrum of **PC-Py1-TIPS**

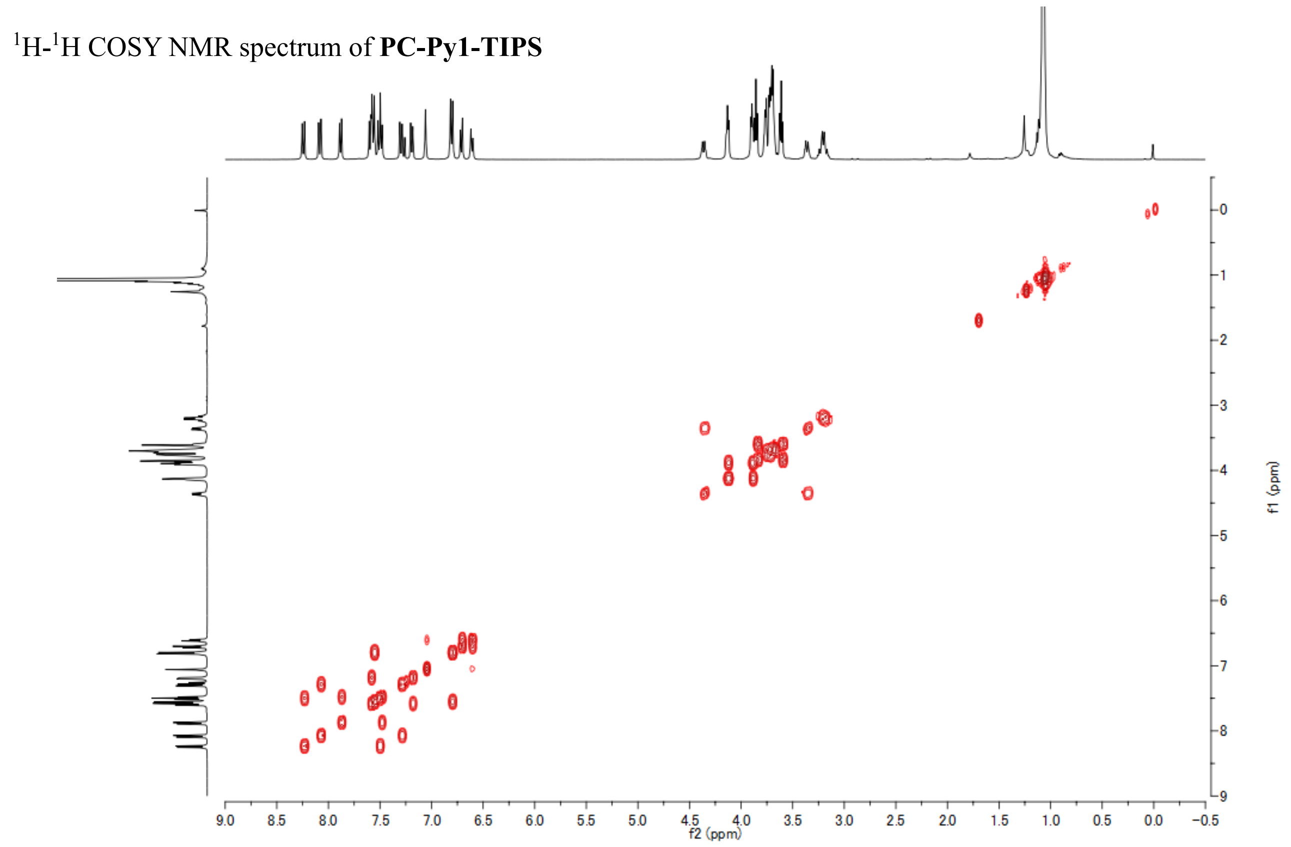


$^{1}$H-$^{1}$H ROESY NMR spectrum of **PC-Py1-TIPS**

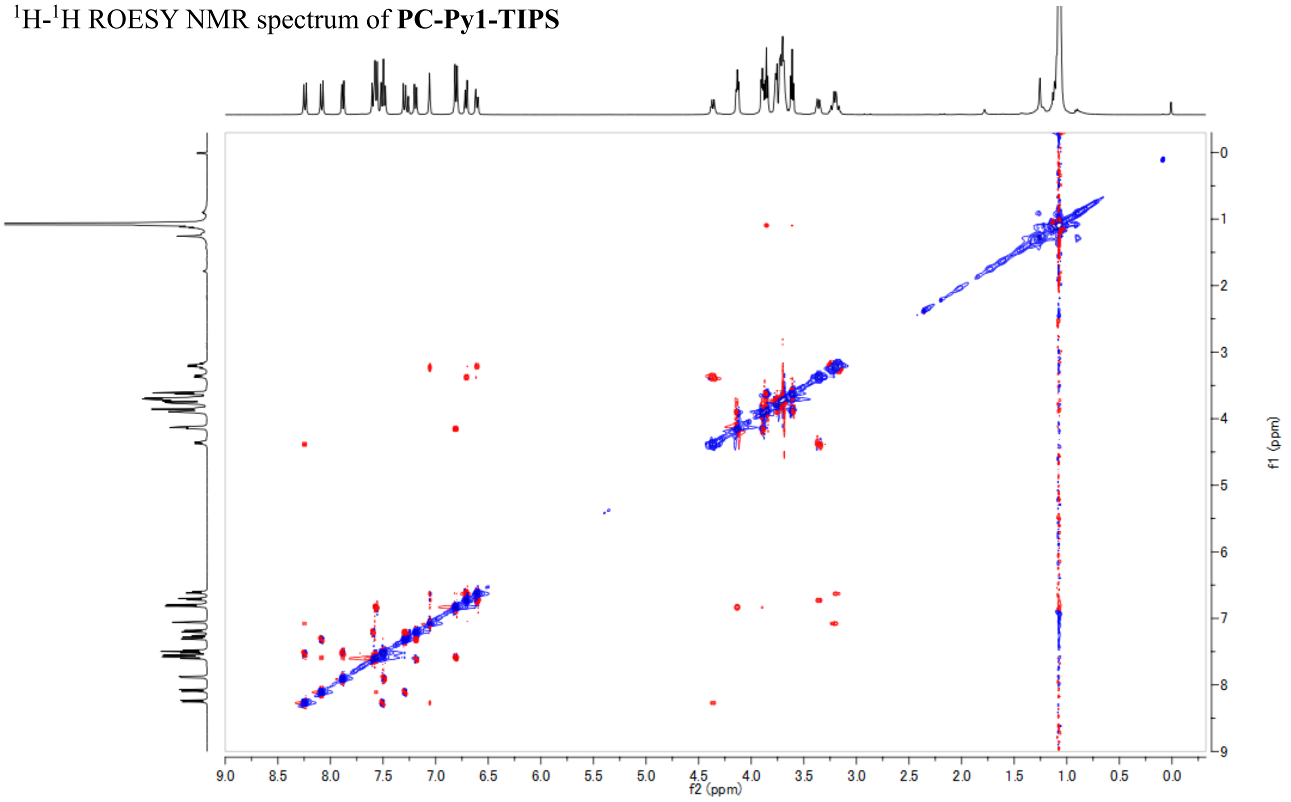

$^{1}H$-$^{13}C$ HMBC spectrum of **PC-Py1-TIPS**

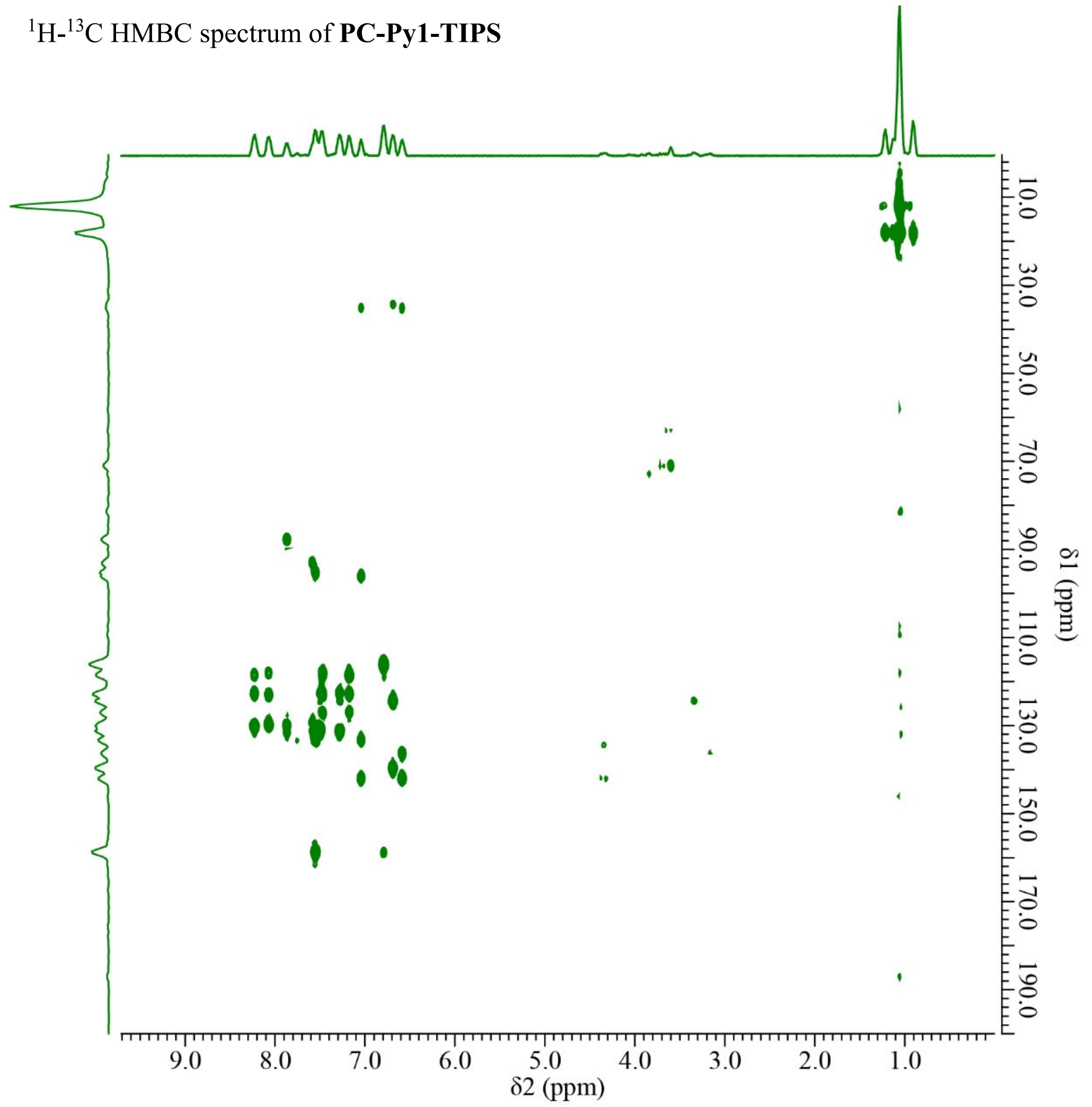

$^{1}$H NMR spectrum of **PC-Py1**

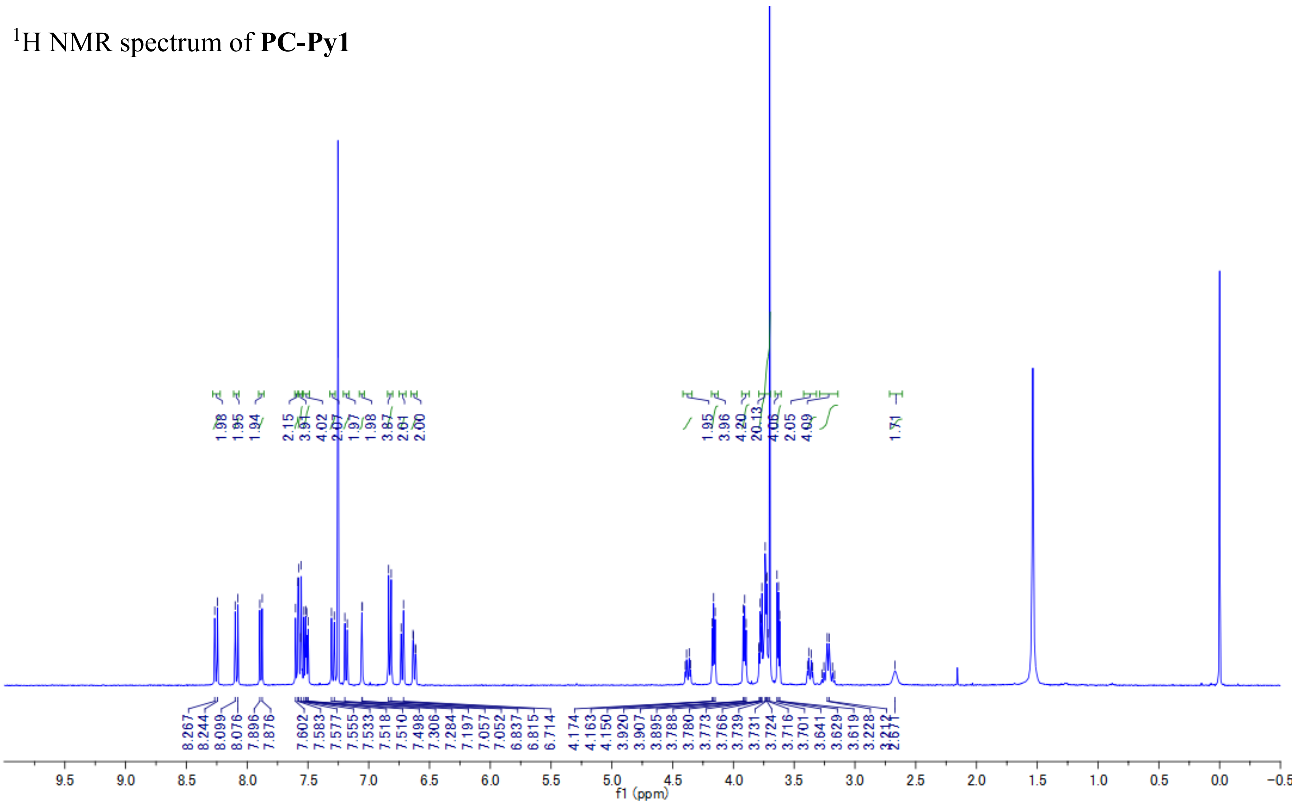


$^{13}$C NMR spectrum of **PC-Py1**

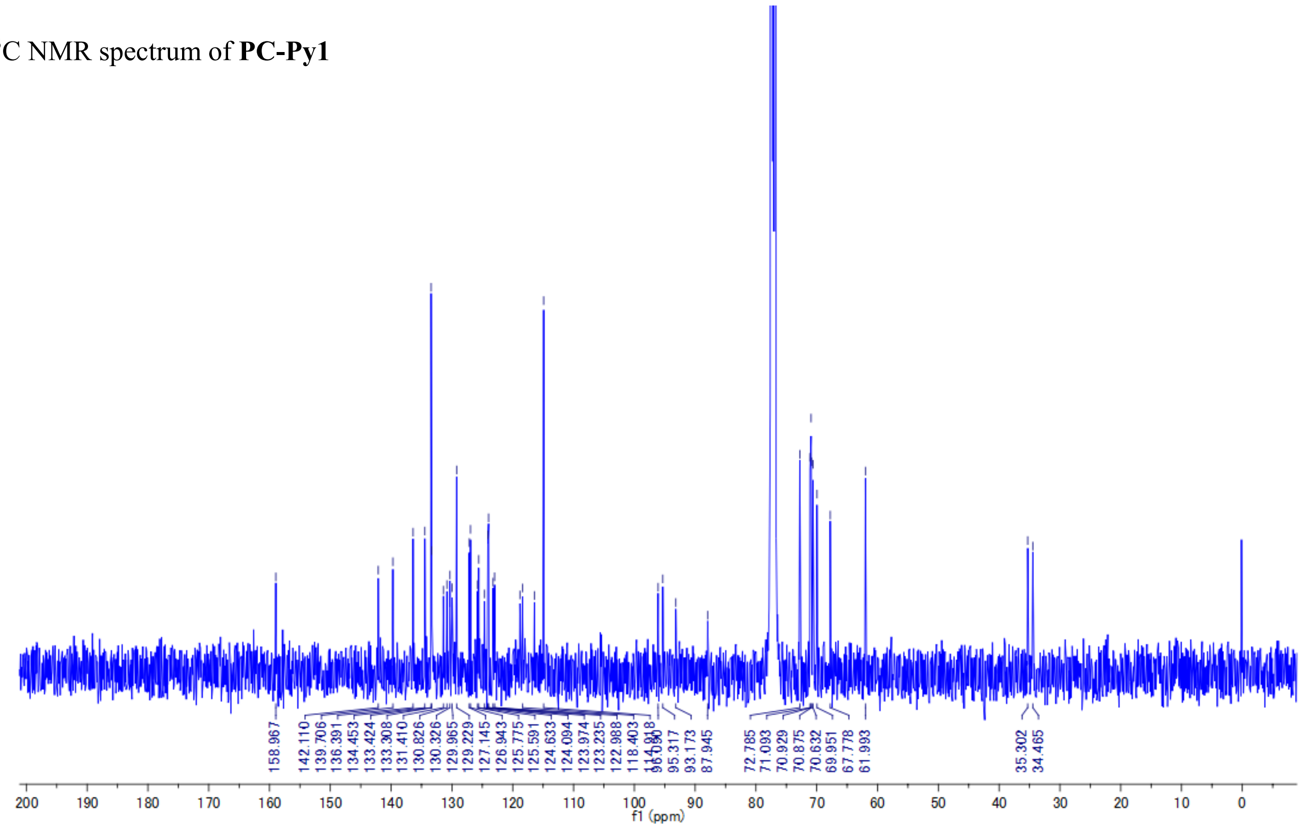

$^1$H NMR spectrum of **Py**

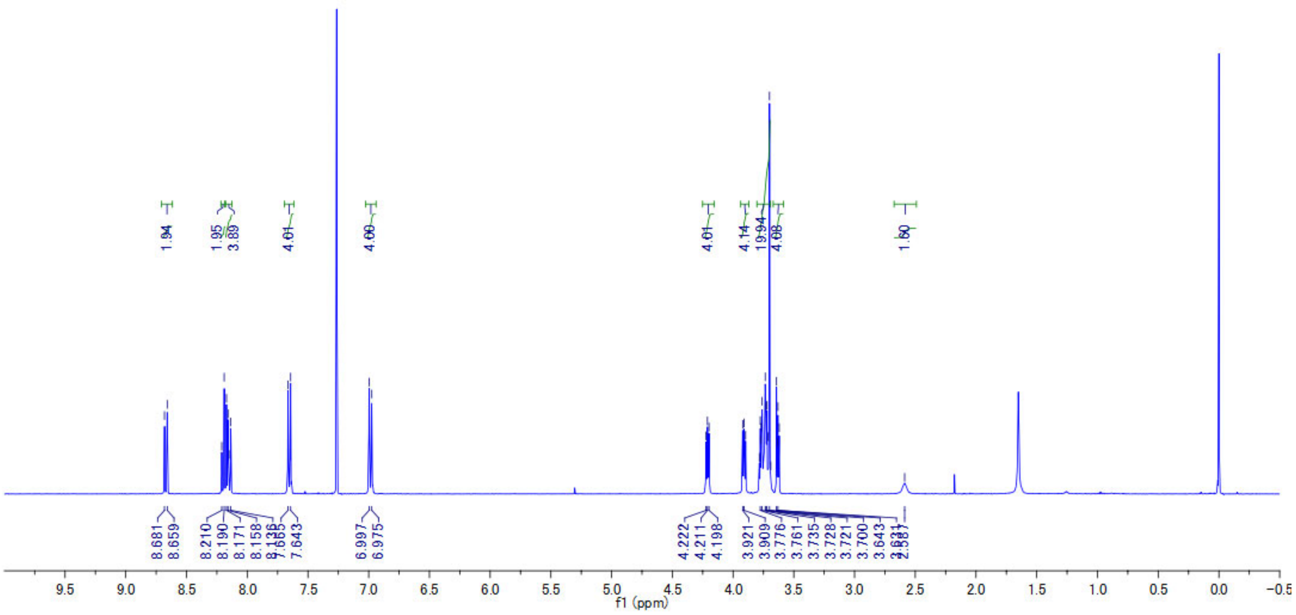


$^{13}$C NMR spectrum of **Py**

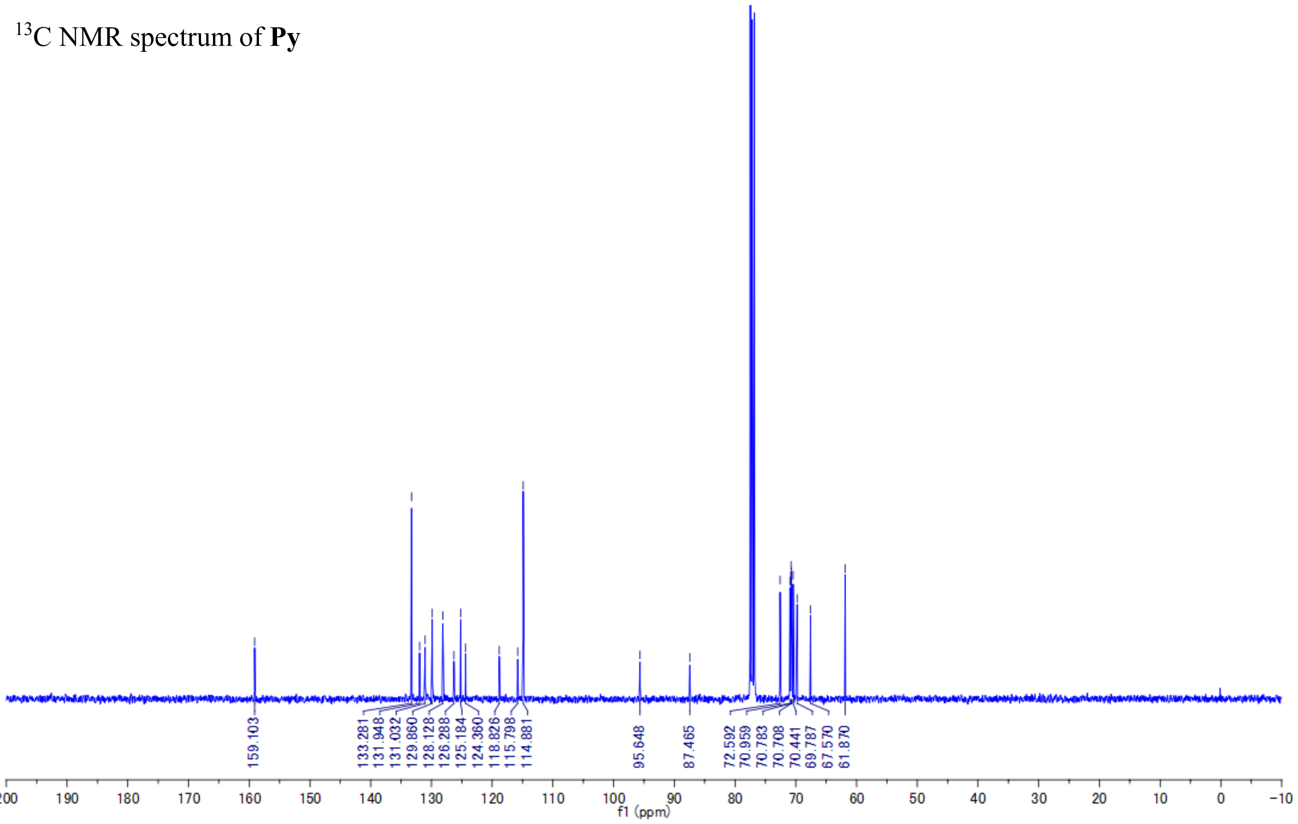

$^{1}H$-$^{13}C$ HMBC spectrum of **Py**

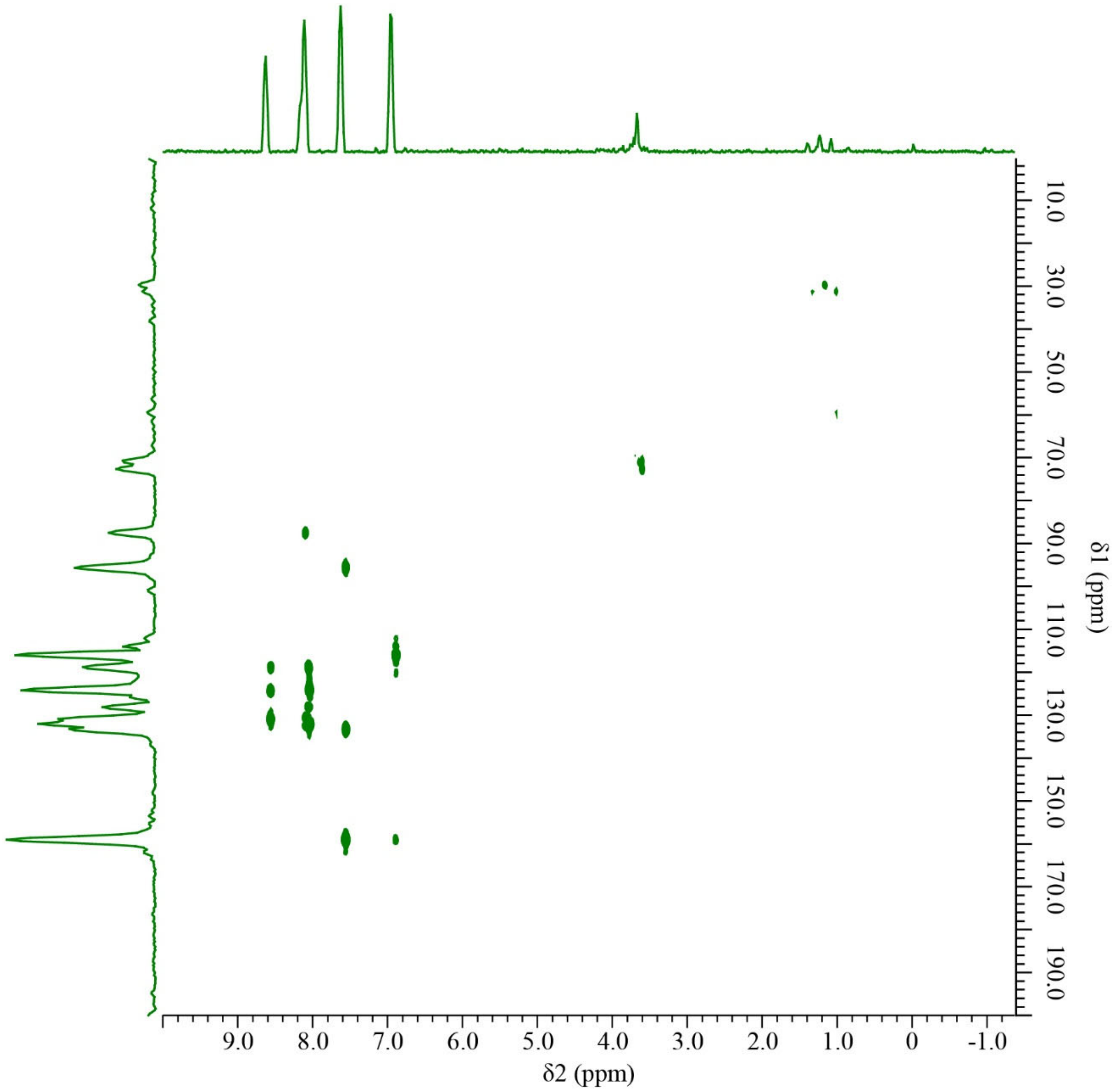

$^{1}$H NMR spectrum of compound **7**

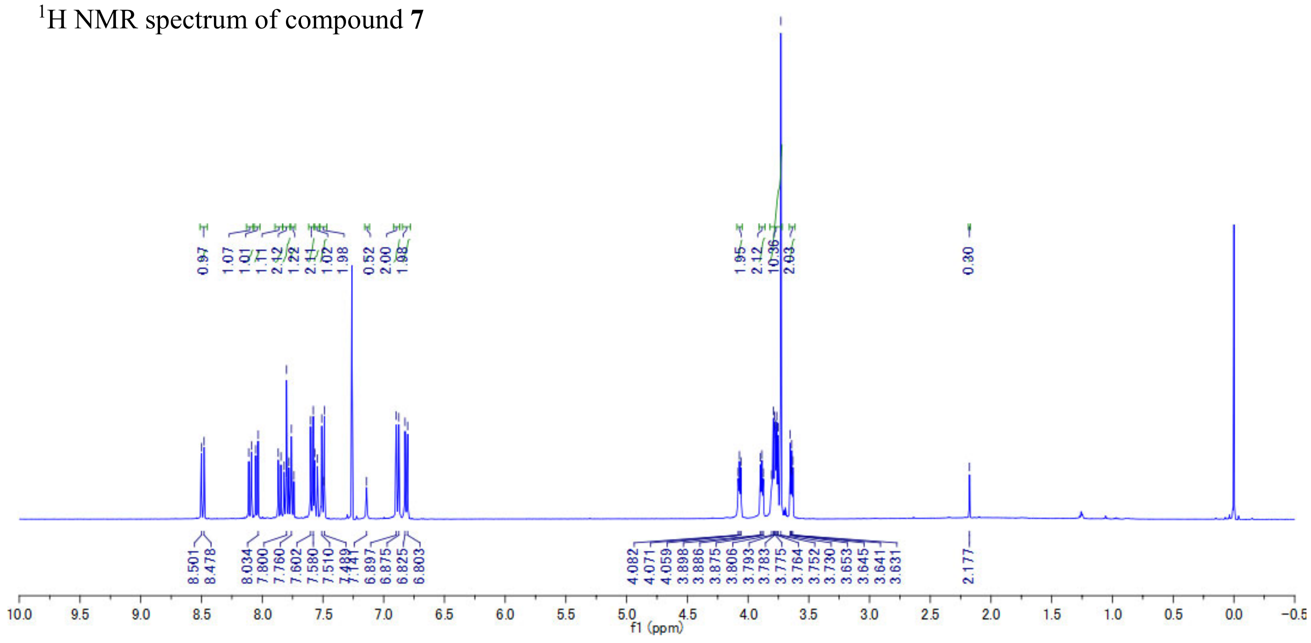

$^{13}$C NMR spectrum of compound **7**

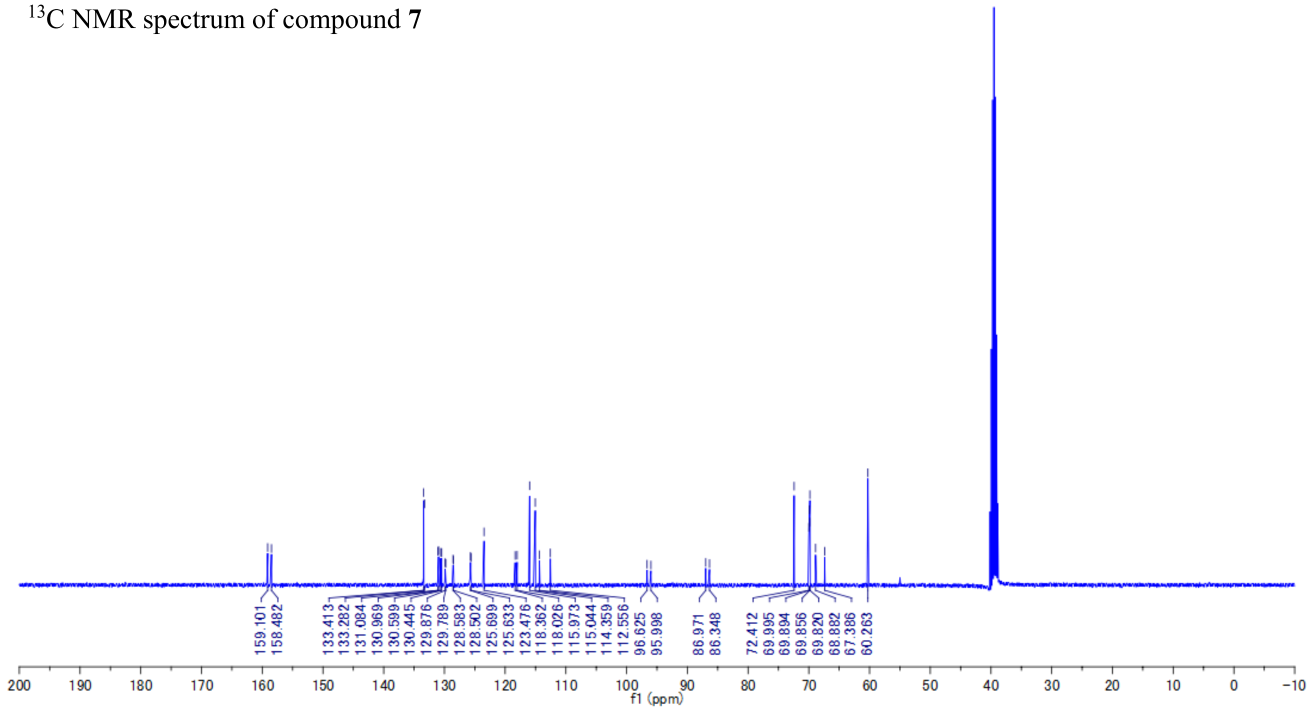

$^1H$ NMR spectrum of **PC-Py2**

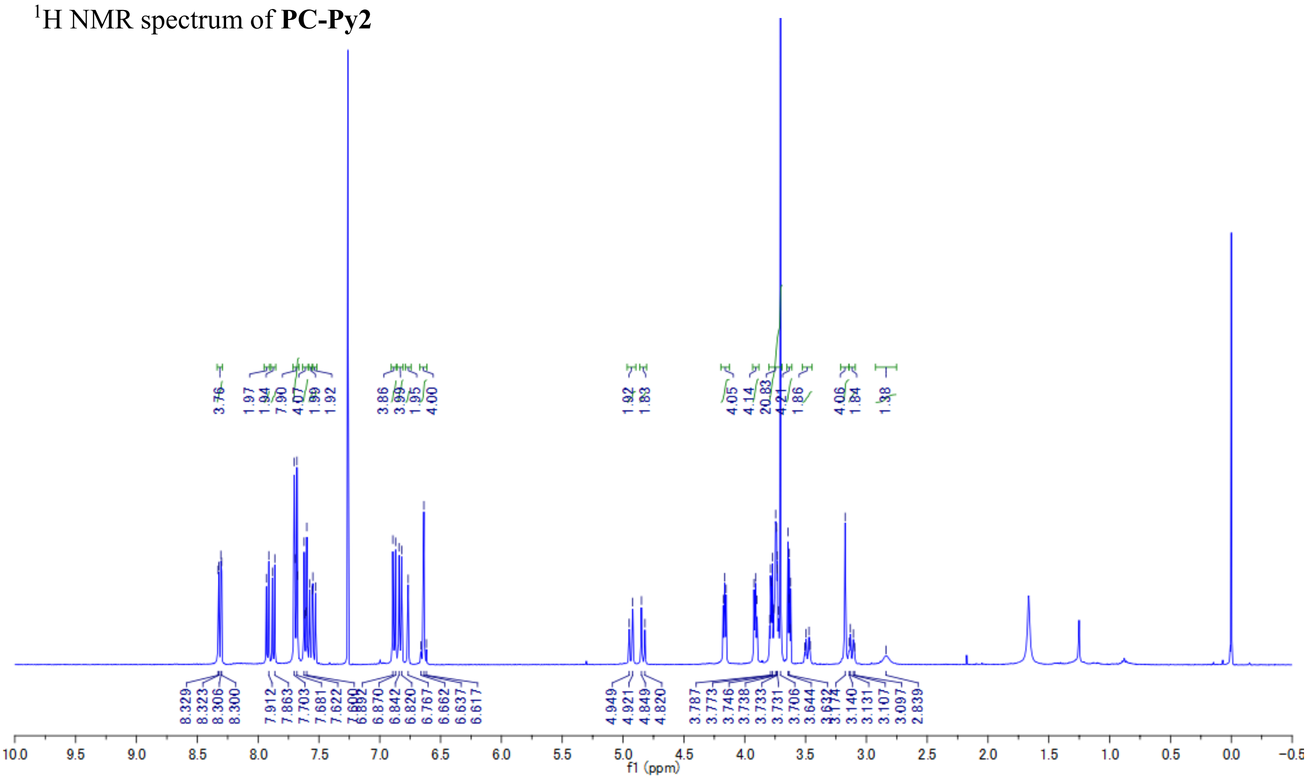

$^{13}C$ NMR spectrum of **PC-Py2**

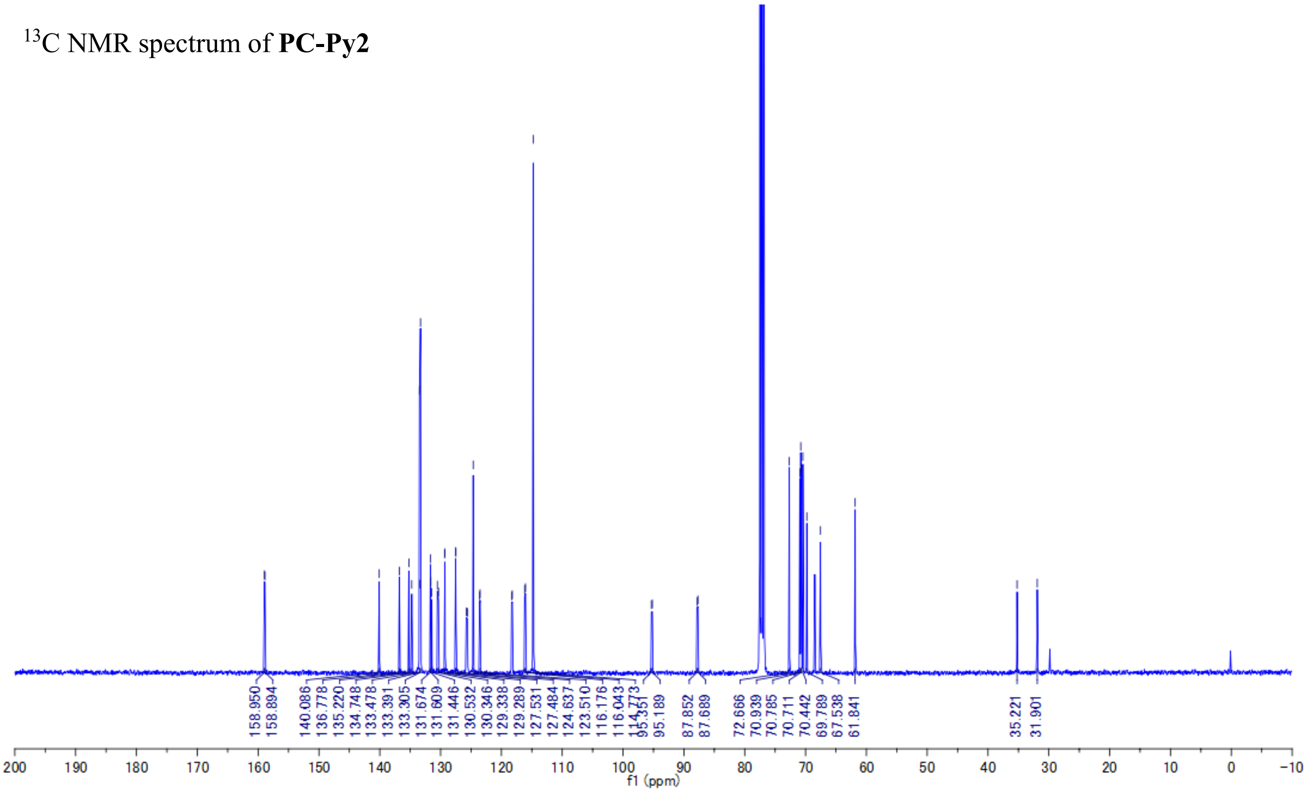

$^{1}$H NMR spectrum of compound **8**

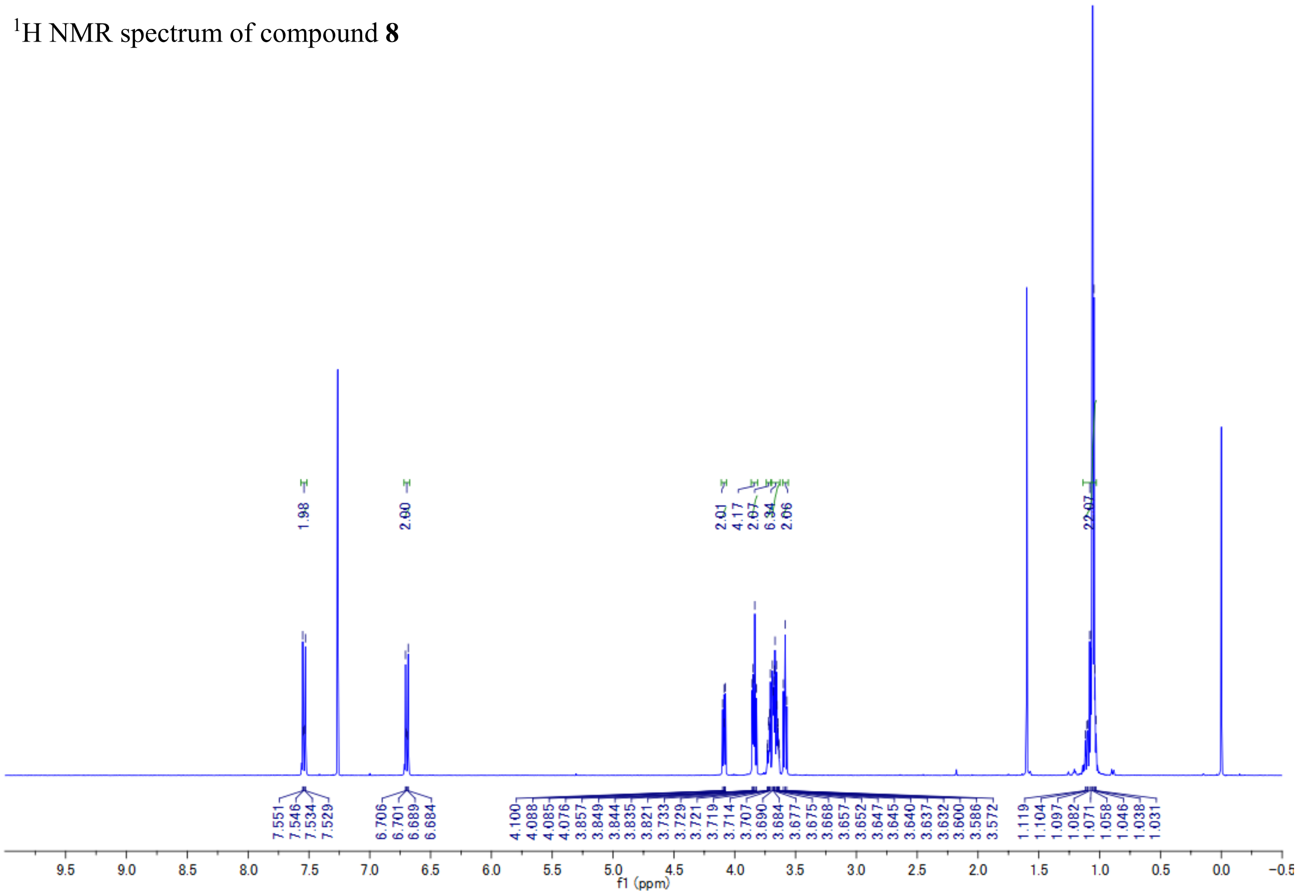

$^{13}$C NMR spectrum of compound **8**

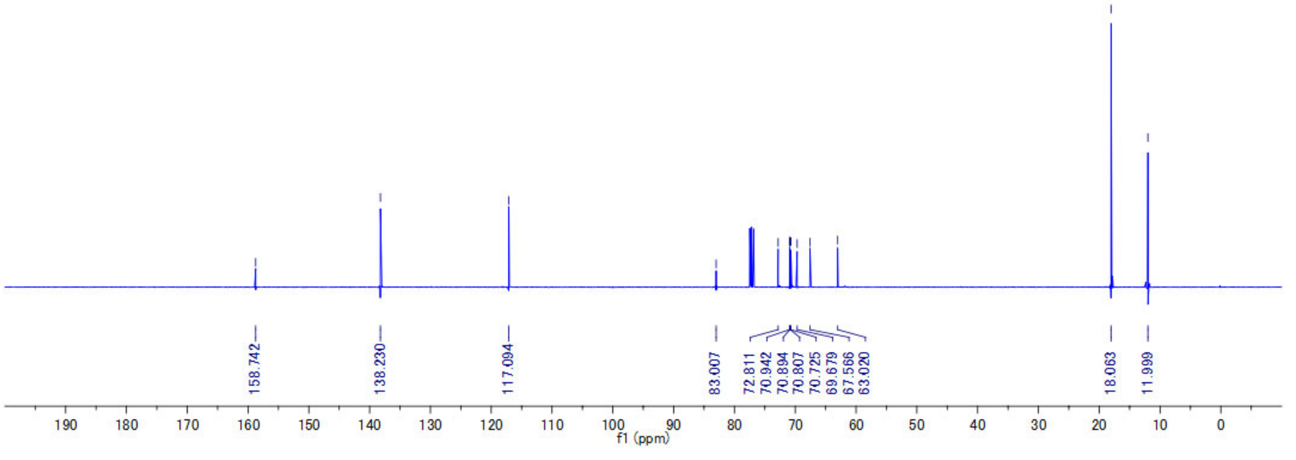

$^{1}$H NMR spectrum of compound **9**

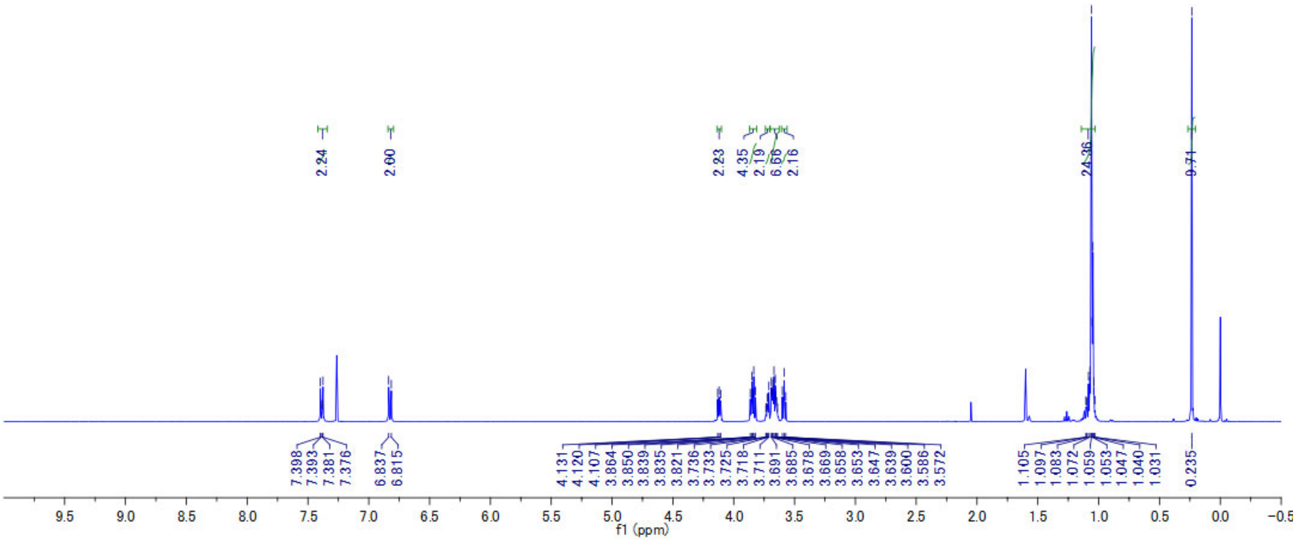


$^{13}$C NMR spectrum of compound **9**

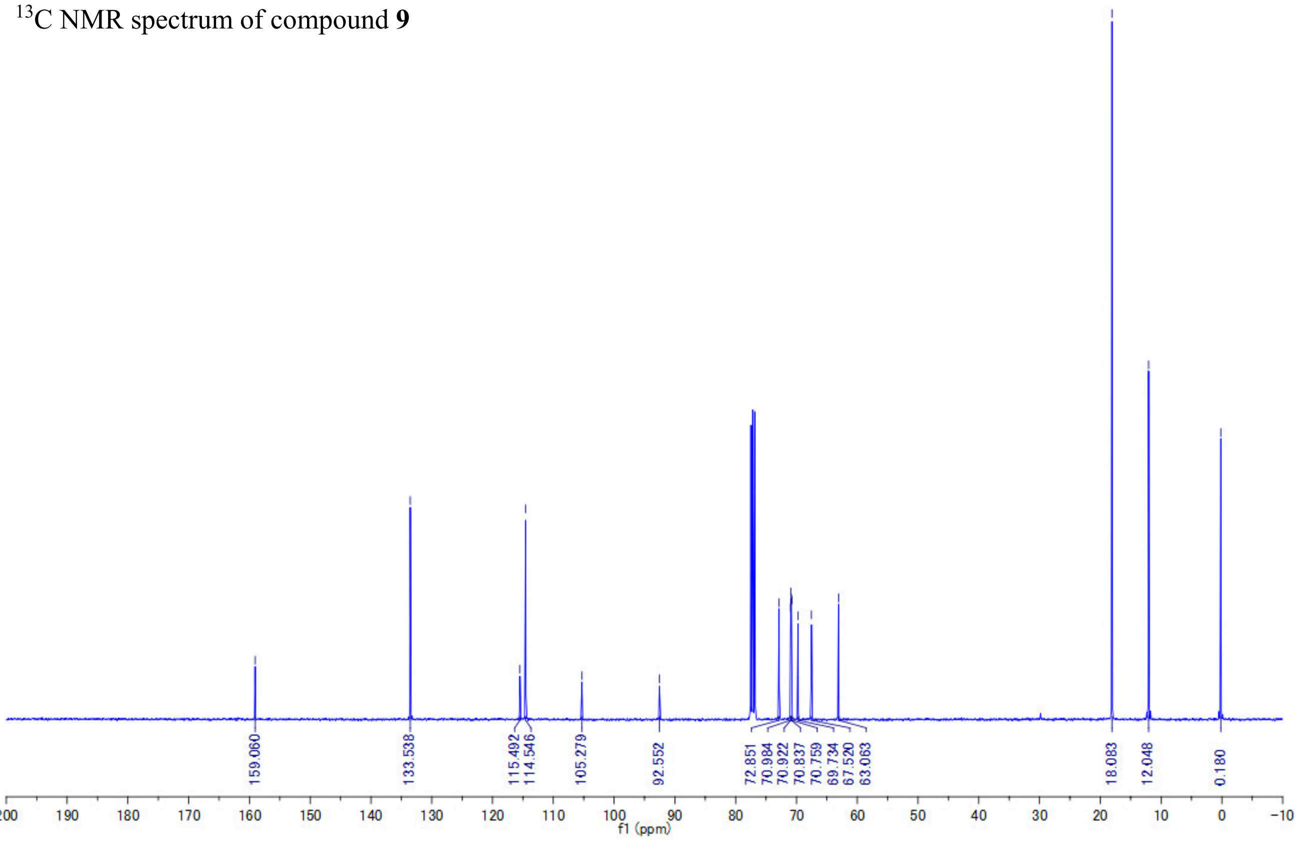

$^{1}$H NMR spectrum of compound **10**

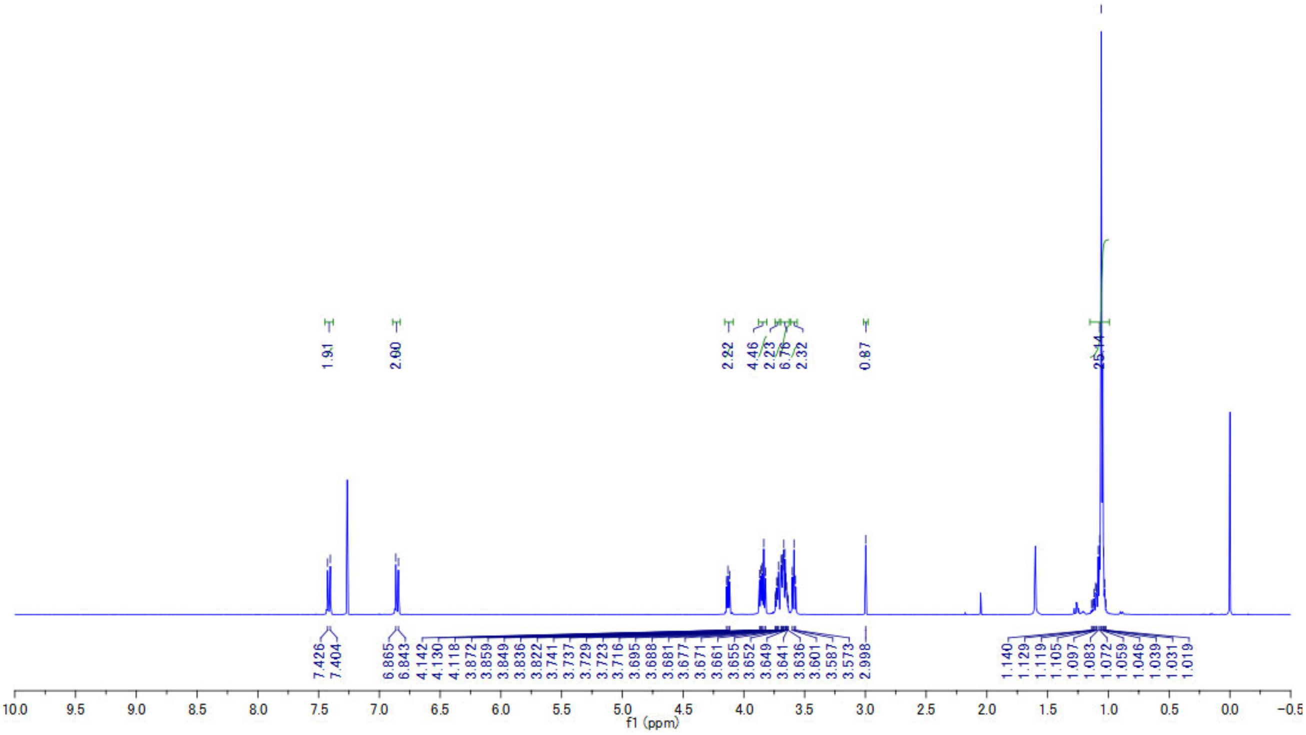

$^{13}$C NMR spectrum of compound **10**

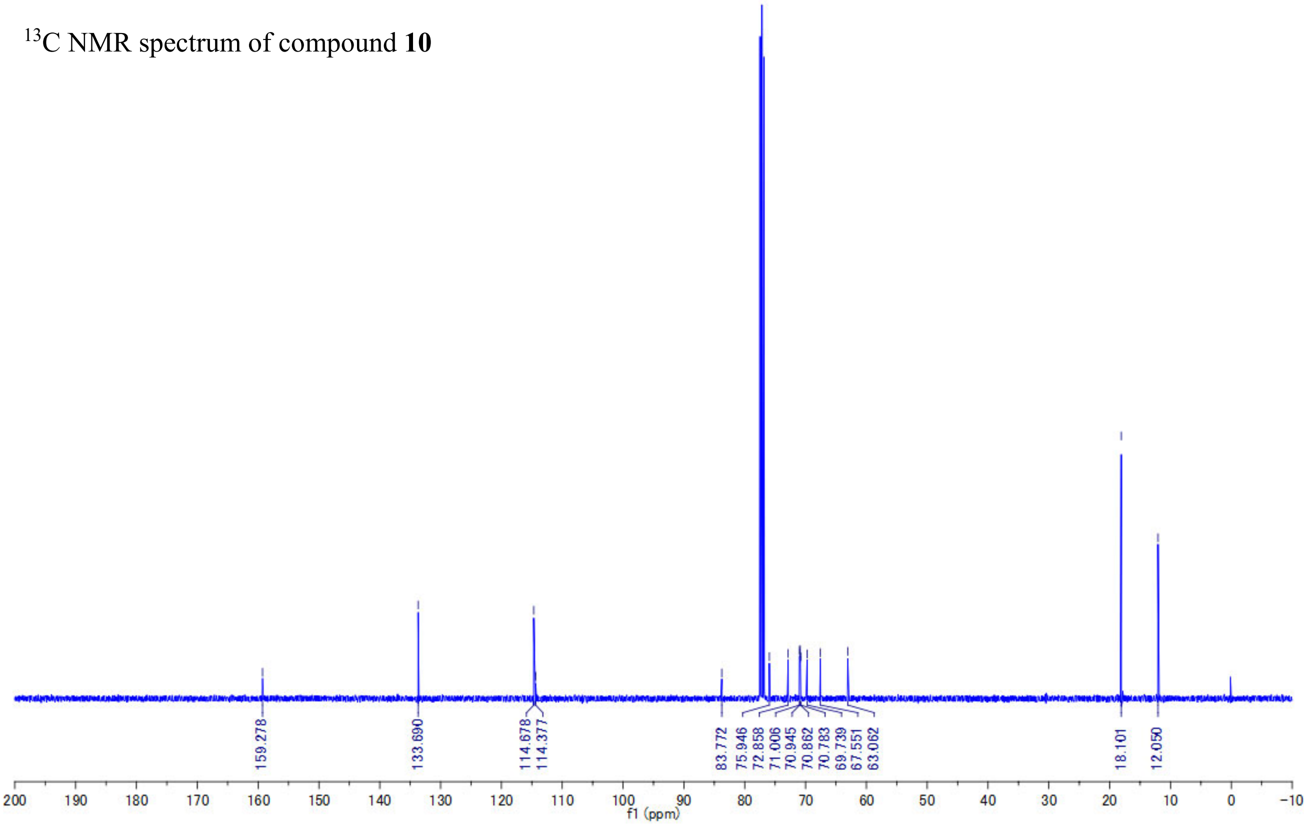

$^{1}$H NMR spectrum of compound **11**

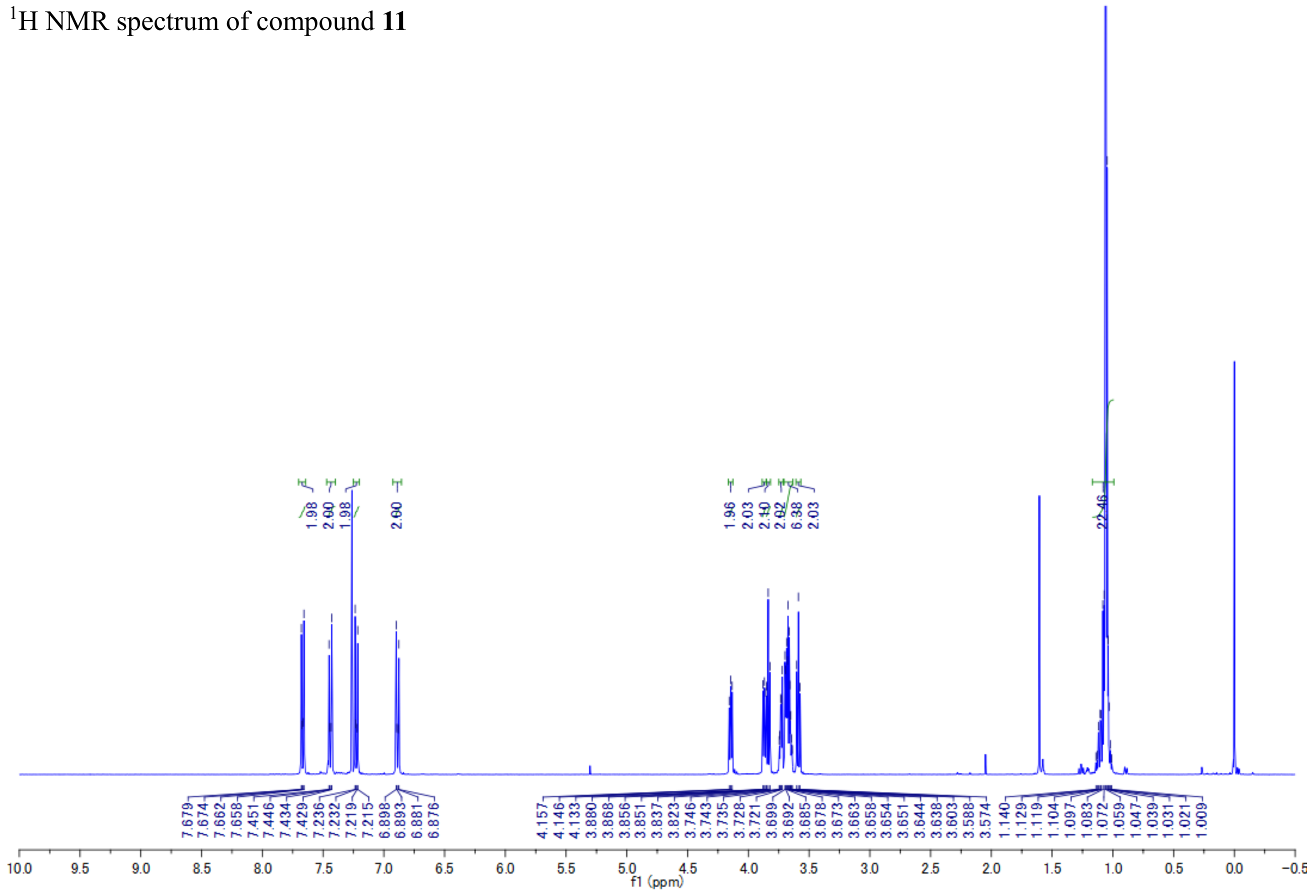

$^{13}$C NMR spectrum of compound **11**

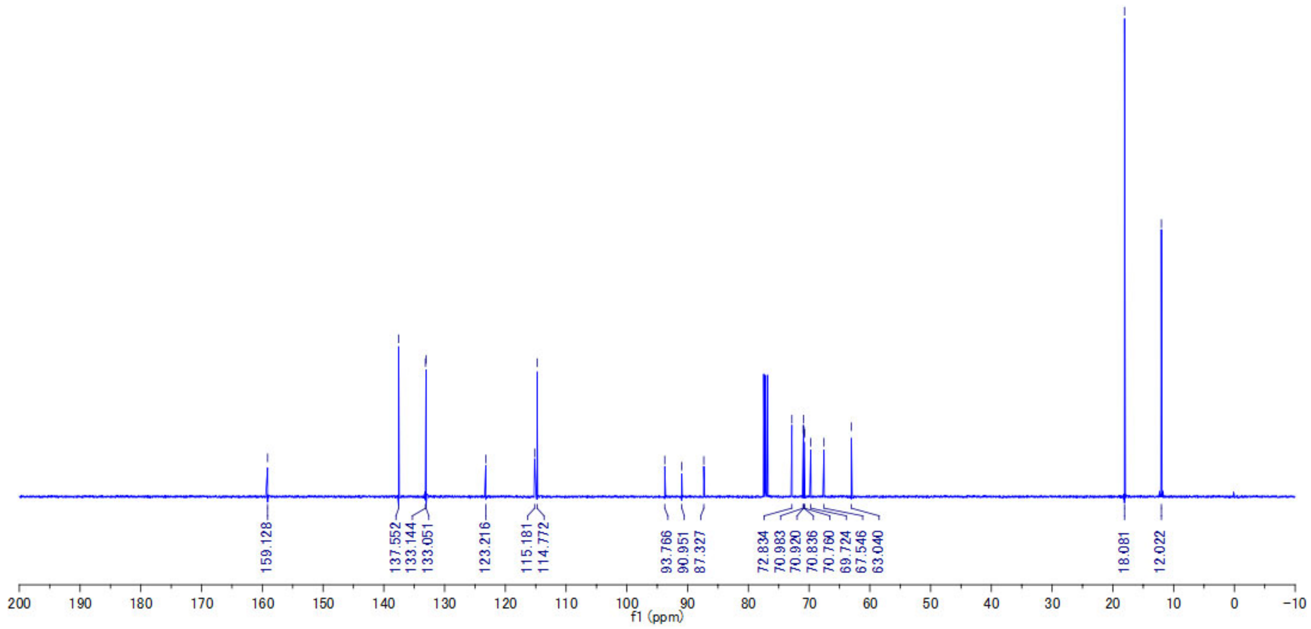

$^{1}$H NMR spectrum of compound **12**

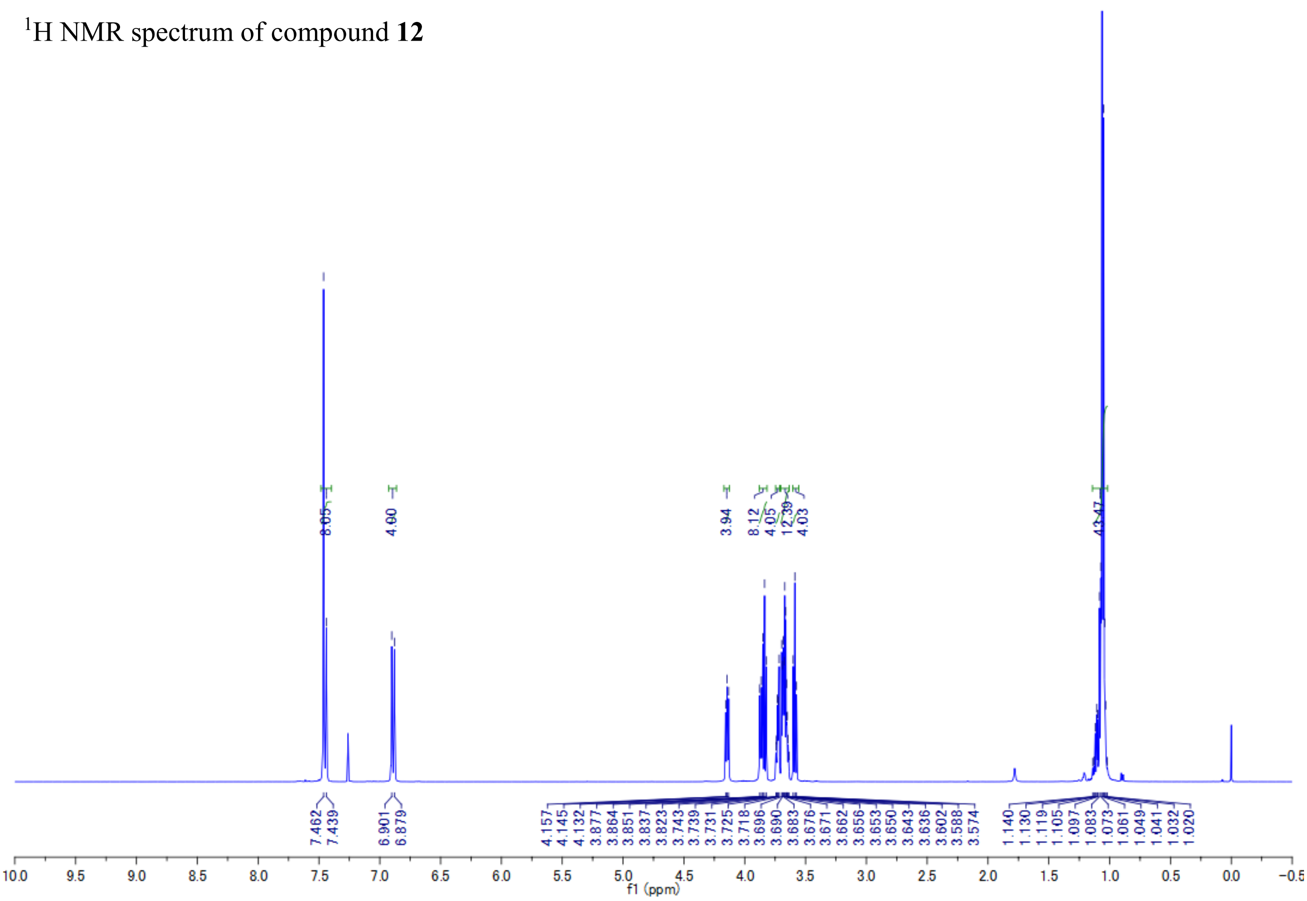


$^{13}$C NMR spectrum of compound **12**

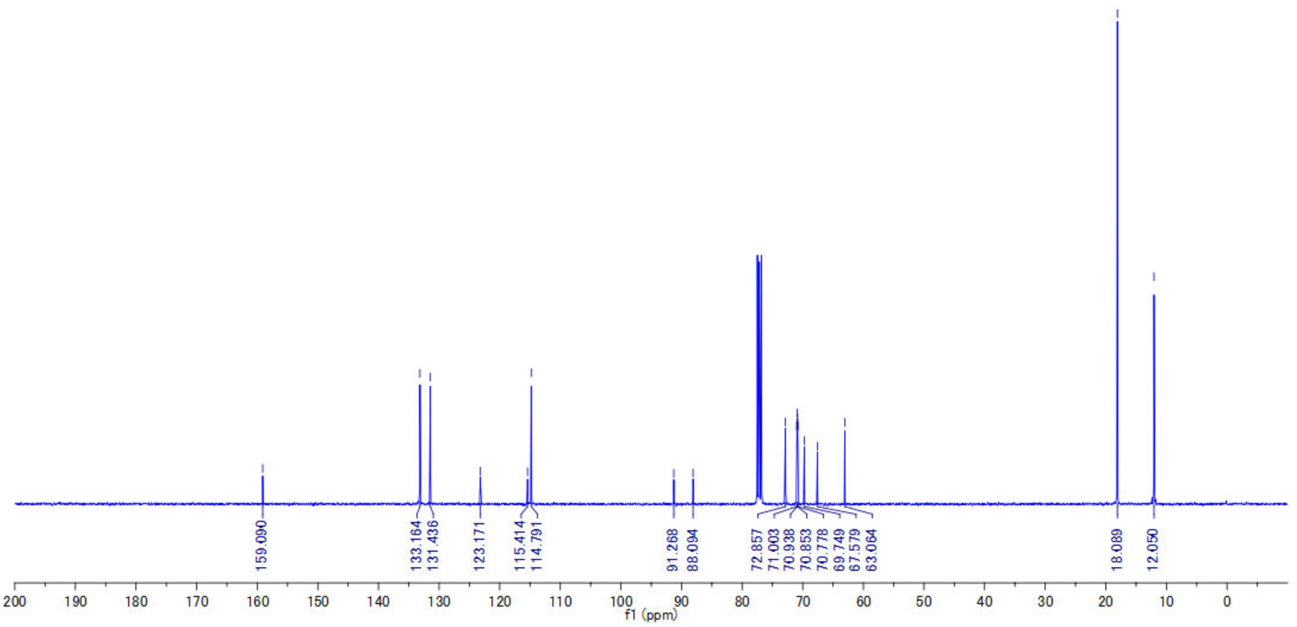

$^{1}$H NMR spectrum of compound **13**

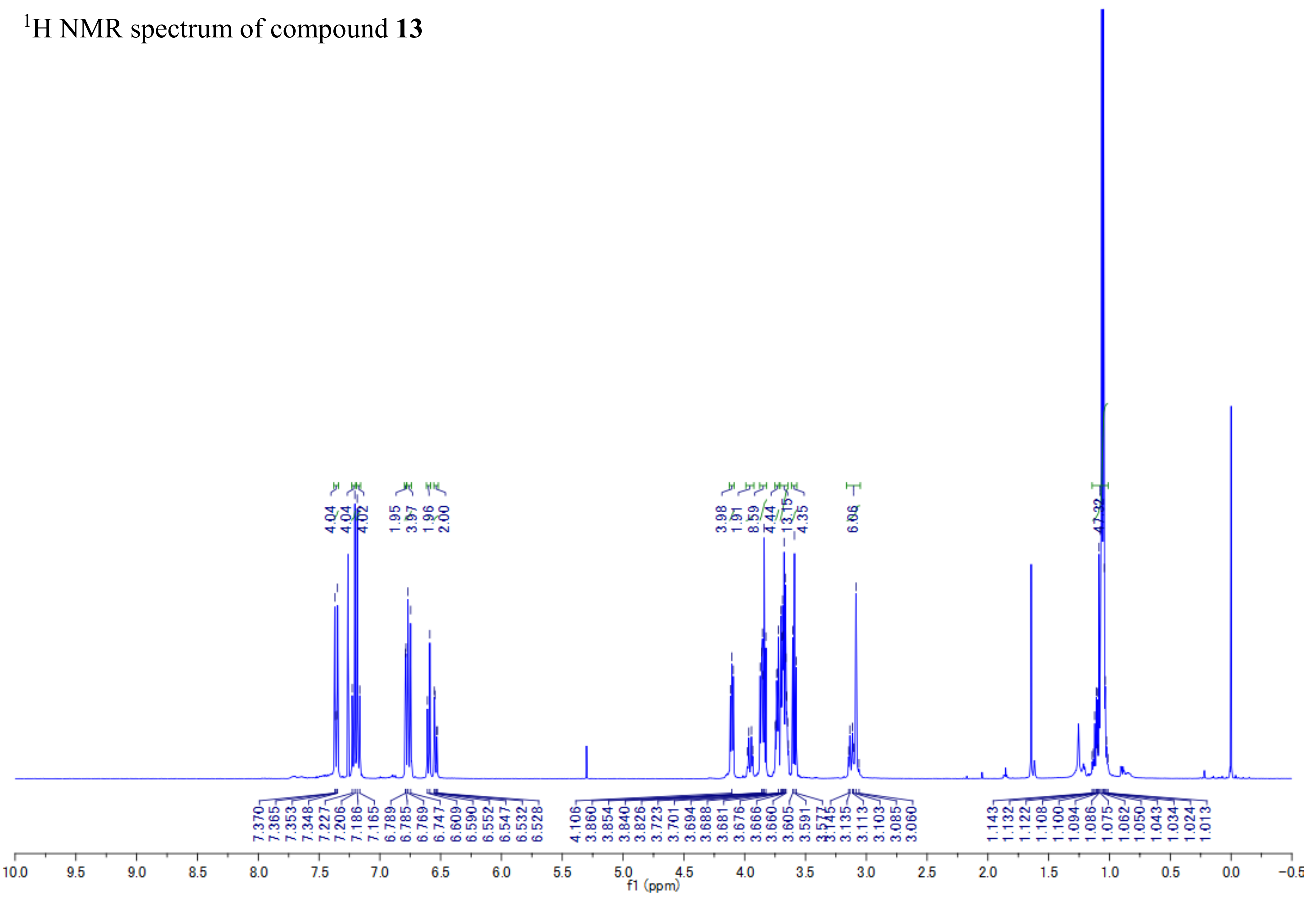

$^{13}$C NMR spectrum of compound **13**

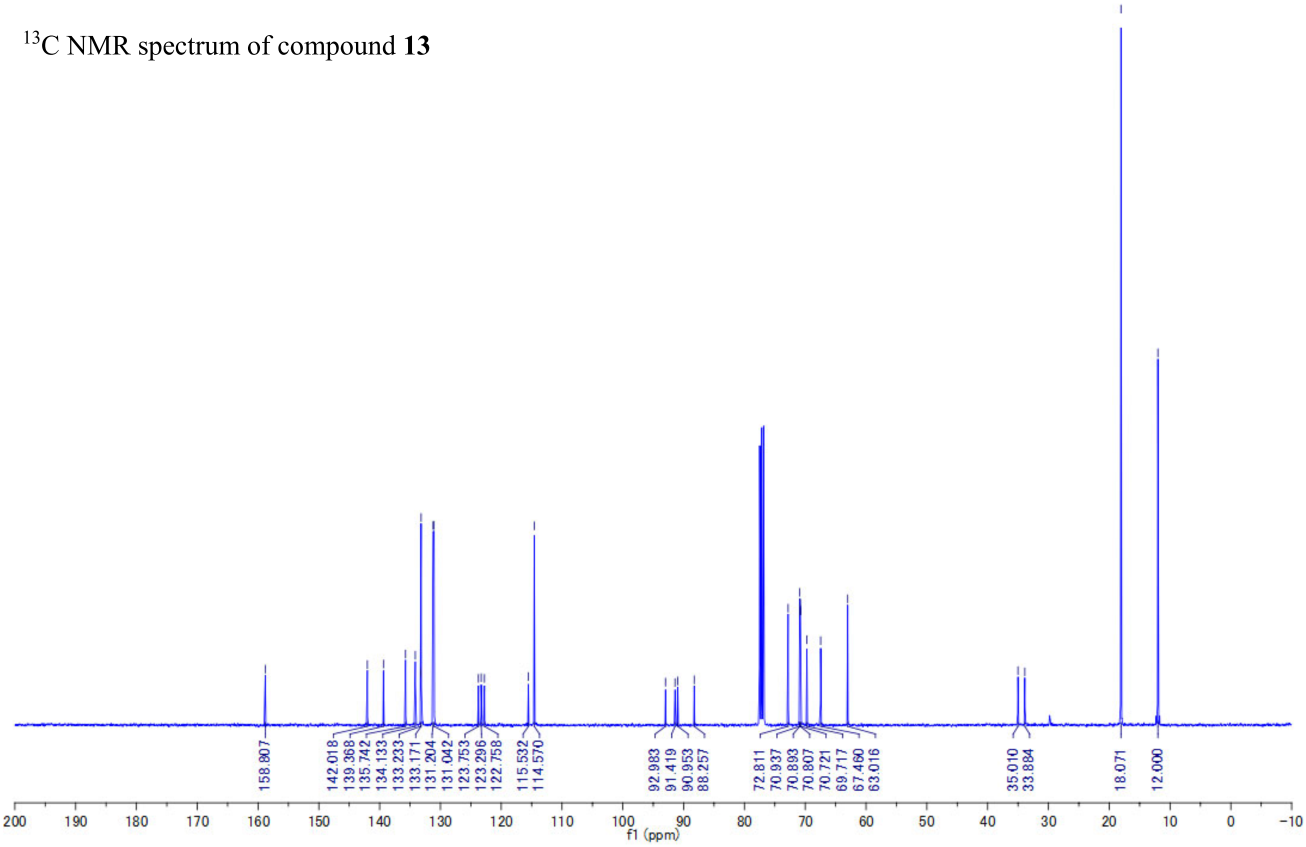

$^{1}$H NMR spectrum of **PC-Be**

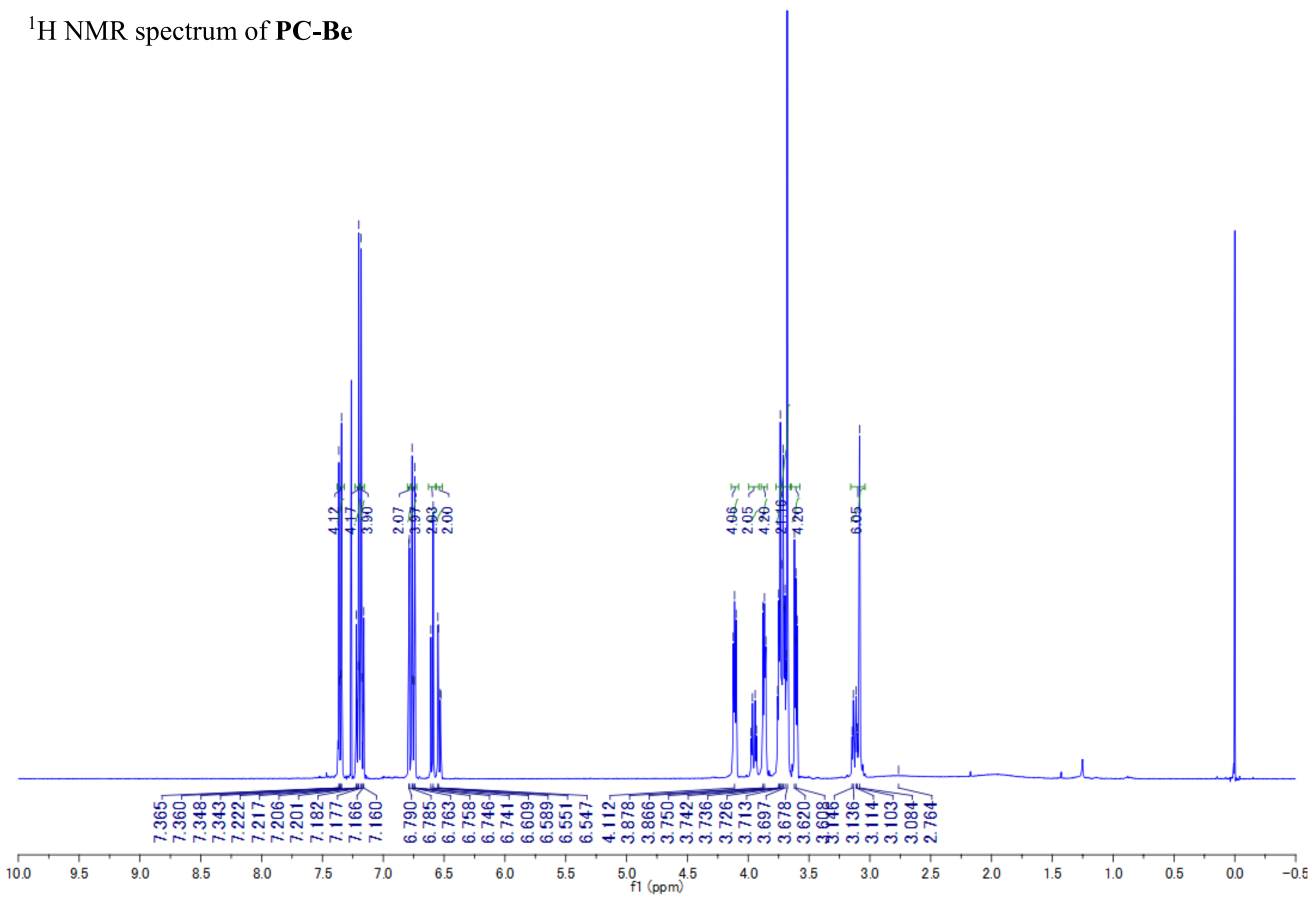

$^{13}$C NMR spectrum of **PC-Be**

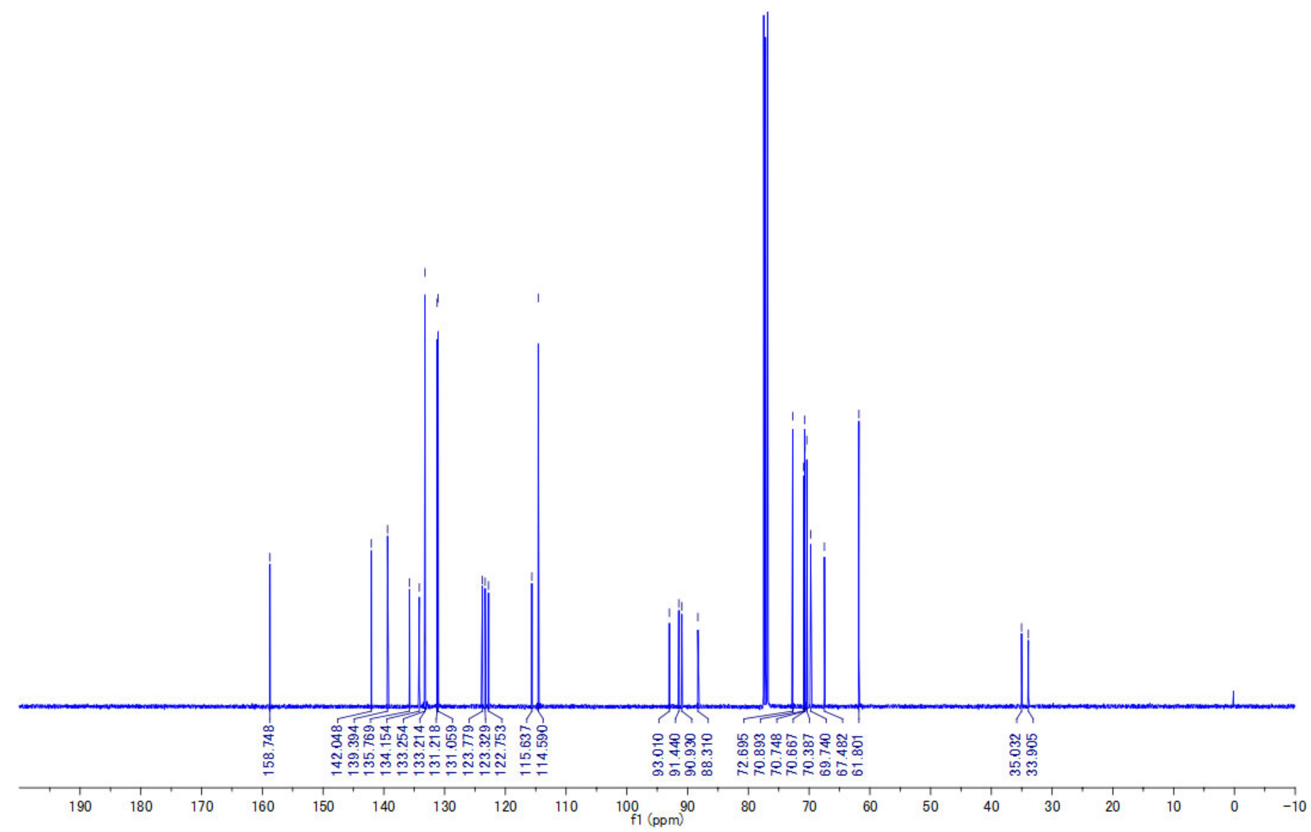

$^{1}$H NMR spectrum of **Be**

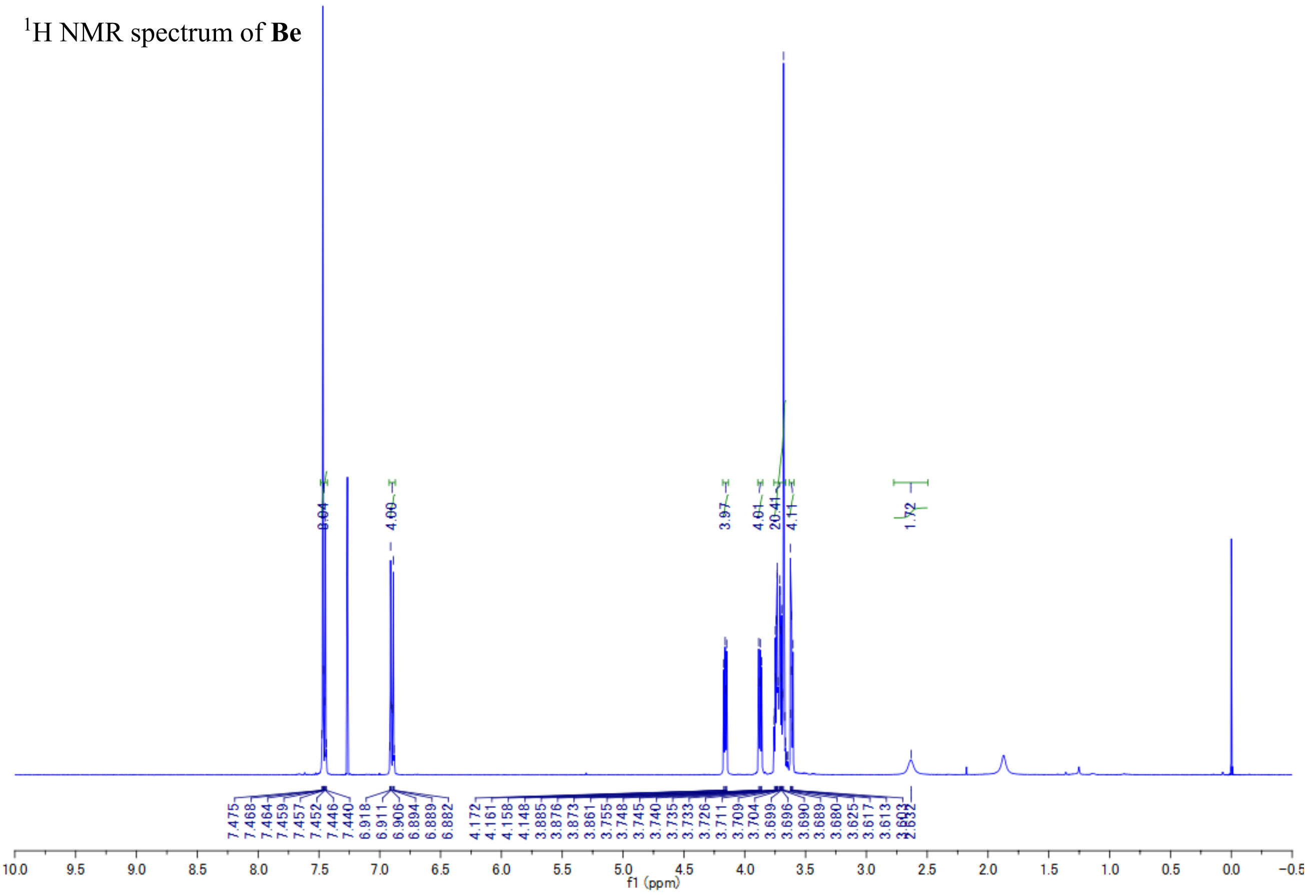

$^{13}$C NMR spectrum of **Be**

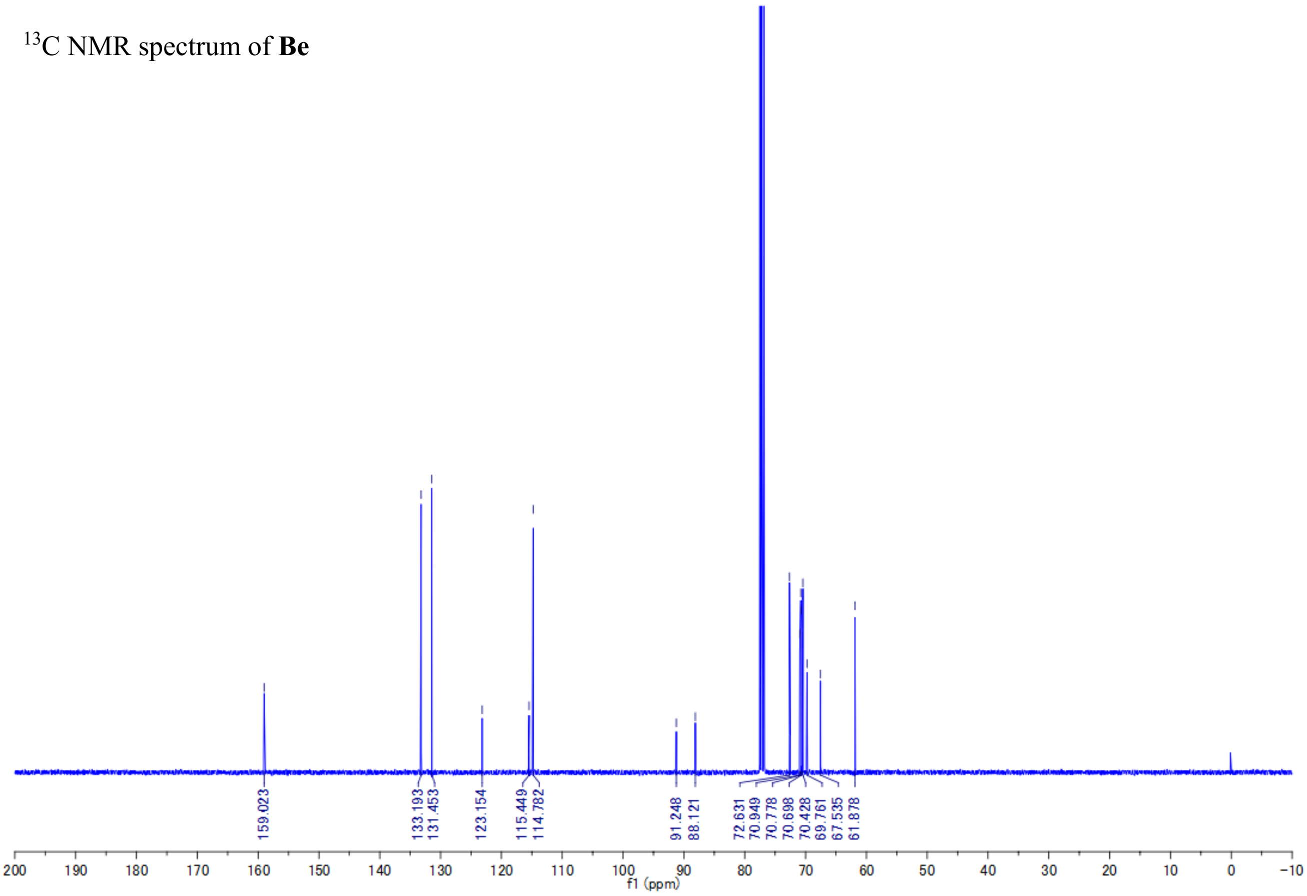

## Supporting references


[S1] S. P. Jagtap, D. M. Collard, “2D Multilayered π-stacked conjugated polymers based on a U-turn pseudo-geminal [2.2]paracyclophane scaffold” *Polym. Chem.* **2012**, *3*, 463−471.

[S2] Y. Sagara, H. Traeger, J. Li, Y. Okado, S. Schrettl, N. Tamaoki, C. Weder, “Mechanically Responsive Luminescent Polymers Based on Supramolecular Cyclophane Mechanophores” *J. Am. Chem. Soc.* **2021**, *143*, 5519−5525.

[S3] E. Janett, Y. Bernardinelli, D. Müller, C. G. Bochet, “Synthesis of FMRFaNV, a Photoreleasable Caged Transmitter Designed to Study Neuron-Glia Interactions in the Central Nervous System” *Bioconjug. Chem.* **2015**, *26*, 2408−2418.

[S4] Y. Sagara, C. Weder, N. Tamaoki, “Asymmetric Cyclophanes Permit Access to Supercooled Nematic Liquid Crystals with Stimulus-Responsive Luminescence” *Chem. Mater.* **2017**, *29*, 6145−6152.

[S5] E. A. Truesdale, D. J. Cram, “Macro Rings. 49. Use of Transannular Reactions to Add Bridges to [2.2]Paracyclophane” *J. Org. Chem.* **1980**, *45*, 3974−3981.”
